# Supplementary material for: Randomised controlled trial of a behaviour change physiotherapy intervention to increase physical activity following hip and knee replacement: the PEP-TALK trial
Source: BMJ Open. 2022 May 31;12(5):e061373. doi: 10.1136/bmjopen-2022-061373 (PMC9157340; doi:10.1136/bmjopen-2022-061373)
Supplement: Supplementary data [file bmjopen-2022-061373supp001.pdf]

Supplementary File 1: PEP-TALK programme intervention outline and development

Background

Total hip (THR) and knee replacement (TKR) are two highly successful orthopaedic procedures which reduce pain for people with osteoarthritis (1-2). Over 206,000 THRs and TKRs were performed in the UK in 2018 (1). Approximately 90% of patients report significant improvements in pain and physical function after three to 12 months (2-3). However medical co-morbidities are common in this population. These include hypertension (56%) (4) and cardiovascular disease (20%) (5), diabetes (16%) (5) and multi-joint pain (57%) (4). Twenty-seven percent of people who undergo joint replacement have three or four comorbidities (5). These have a significant negative impact on both health-related quality of life and societal burden (6-7).

Historically, it has been assumed that people are more active following TKR and THR through the amelioration of their joint pain (8). However physical activity, for most patients, remains the same from pre- to post-operatively, and in some instances declines (8-9). Physical activity can significantly reduce the symptoms associated with common comorbidities (10). Participating in regular physical activity can decrease the risk of cardiovascular disease by 52% (11), diabetes by 65% (12) and some cancers by 40% (13). It can reduce all-cause mortality by 33% and cardiovascular mortality by 35% (14). Supporting people to be more physically active can improve patient health and decrease economic burden on health services.

A systematic review identified several barriers and facilitators associated with physical activity following TKR and THR (9). From this, four key mechanisms of action were identified for targeting. These were:

(1) Psychoeducation (knowledge/information) to increase self-efficacy.
(2) Reducing fear-avoidant behaviours in response to unhelpful beliefs about activity jeopardising recovery or damaging the implant.
(3) Providing opportunities for personal enjoyment of the physical activity.
(4) Enabling social contact, peer-support and advice from previous patients (encouraging positive coping behaviours).

Systematic reviews of behaviour change interventions have identified that those with a theoretical basis are more effective than those without (15-16). The Social Cognitive Theory (SCT) (17) has been commonly used to understand physical activity behaviour in older adults. The theory targets self-efficacy, goals, outcome expectations and socio-structural factors. Bandura (17) hypothesises that behaviour (physical activity level) is influenced by bi-directional relationships with personal factors (cognitive, emotional and physical) and environment. The cognitive behavioural approach uses techniques to identify and target unhelpful thoughts and behaviours in order to produce adaptive thoughts, behaviours, emotions and physiological responses.

Using the SCT framework, we reviewed evidence on the effectiveness of behaviour change techniques for older adults to improve physical activity. These were then compared to the systematic review regarding patients' perspectives post-TKR/THR (9) to for the four key SCT targets outlined below.

1. *Self-Efficacy: A person's belief in their own ability to perform a behaviour*

General self-efficacy: Quantitative and qualitative systematic reviews examining barriers and facilitators for older adults to increase physical activity have identified specific beliefs which could reduce an individual's general self-efficacy (9, 18-21). These include: stigma, body image (20) and ageing stereotypes (19). Unhelpful beliefs can be identified and explored using cognitive behavioural techniques to increase self-efficacy. The evidence also identified tools to increase general self-efficacy

which include the credibility of instructors and the information/physical activity tasks they provide (19-20, 22).

Self-efficacy to cope with barriers: Barrier identification and problem-solving are two key behaviour change techniques previously identified from the literature. Barriers can be socio-structural such as lack of access/convenience of facilities (20). Whilst these types of barriers cannot be changed by the PEP-TALK intervention, we can facilitate problem-solving strategies to help overcome such barriers.

The intervention programme will be a group-based rolling programme consisting of people in different stages of their behaviour change process. Peers may suggest ideas to other members in addition to ideas from instructors (20). Barriers may also be cognitive beliefs such as a fear of increasing physical activity in case of damaging the implant (9). These beliefs can be targeted with cognitive behavioural strategies.

Task efficacy: Previous literature has consistently reported that if someone has struggled with performing physical activity in the past, they will understandably have poor self-efficacy for performing physical activity tasks in the future (9, 23-24). We will target this by encouraging supportive environments to try exercises with physiotherapists (22), vicariously learning from other patients following THR or TKR (23) and tailored exercises to meet their individual needs (19). This should theoretically increase self-efficacy and the likelihood of greater physical activity engagement (17).

Somatic and emotional states influence self-efficacy (17). Experiencing stress/tension (emotional), fatigue and pain (somatic) can be interpreted by individuals as an indication that they cannot or should not be active. This consequently lowers their self-efficacy. This will be targeted with psychoeducation regarding relationships between mood and pain to physical activity. Conversely positive mood often increases self-efficacy. French et al (23) identified rewards contingent on attempts to perform the behaviour to be a key behaviour change technique for older adults in increasing physical activity. In our intervention, we will ensure participants are praised or rewarded for attempting to achieve their behavioural goal.

2. *Goals*

The SCT suggests that identifying proximal and distal goals are key to behaviour change (17). While this may be the case for younger adults, in older adults and individuals following THR or TKR specifically, goal-setting has consistently shown not to be a useful technique and not acceptable (9, 22-23). French et al (23) proposes two explanations regarding this change. Firstly, with age, cognitive process of executive functioning (planning, attentional capacity, inhibition of responses or novel actions) decreases to reduce abilities to self-regulate with goal-setting. Secondly, at this life stage, achieving set goals and normative comparison is not as pertinent as it is in earlier life. Therefore, we shall not include goal-setting in this intervention.

3. *Outcome Expectation*

While the motivation for this intervention may be to increase physical activity for improved health, evidence suggests that health improvement is not the salient outcome for older adults following THR or TKR. This population appear more interested in the social aspect and the enjoyment through physical activity (9). The Socioemotional Selectivity Theory (25) is a life-span theory of motivation which suggests that as people age, motivation is influenced more by positive, emotionally meaningful goals and activities and less so by normatively defined goals of health. This is extended by Devereux-Fitzgerald's (22) model of the interplay of factors of acceptability to physical activity interventions for older adults. They identified that interventions which provide the most enjoyment and meaningful value (e.g. social interactions) are the most acceptable (22). Our intervention aims to identify what is meaningful and valuable to participants by consistently asking them to reflect on open questions such as "what do you want to gain from attending this group? What are you enjoying most?" then tailoring

why and how to perform physical activity to meet these needs. We will also consider these factors when discussing maintenance and continuation of increased physical activity, identifying activities which are fun and enjoyable for each person. This can be aided by ideas generated from group members who may be at different stages of the behaviour change process.

4. *Socio-Structural Factors*

Although socio-structural factors are key to the SCT, these are aspects which we cannot change from an intervention perspective. However, we can identify modifiable factors and use problem-solving techniques to overcome barriers or find alternatives options. For example, a patient explains there is no safe pavement to walk along from their house to the shops and consequently the patient always drives. The group could offer local knowledge solutions, perhaps there is a nearby bus which can take the patient into a part of the town with good walkways. If the patient does not want to catch the bus then this belief could be explored to further understand the perceived barrier (lack of knowledge of the bus routes, perceived financial cost). This technique was identified as a key behaviour change technique for older adults in increasing physical activity (23).

In summary, while there are four key constructs in the SCT, we anticipate that self-efficacy is the key construct to target for change. A key barrier, specific to this population, to improve self-efficacy could be targeting the personal beliefs regarding fear of damaging the implant or re-injury (9). We prioritise targeting self-efficacy and fear avoidance as they are two key constructs that will change as a result of our behaviour change techniques to mediate and improve physical activity within this population.

Intervention development

The SCT provides an in-depth psychological model of why people do or do not perform behaviours. These psychological models of behaviour have been successfully synthesised into a pragmatic framework called the Capability, Opportunity, Motivation – Behaviour (COM-B) model (26). To produce the most effective behaviour change intervention, the evidence has been mapped on biopsychosocial determinants of physical activity levels post-THR/TKR from the SCT onto the COM-B model for behaviour change (*as presented in figure below*). This activity is summarised in the table below.

Capability Opportunity Motivation model of Behaviour (COM-B; Michie et al, 2014)

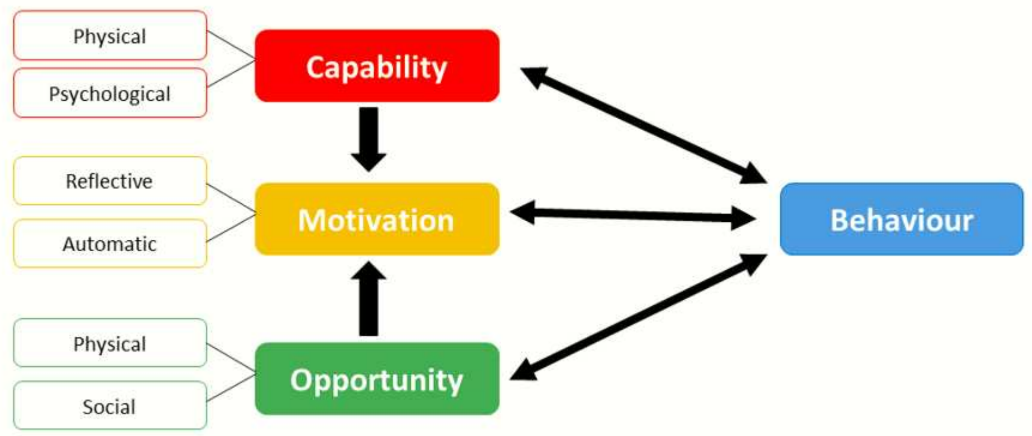

Figure 1: The COM-B model of behaviour change (Michie et al, 2014)

Mapping of the COM-B domains against the PEP-TALK SCT targets.

COM-B Model Component	Domain	Activity
Capability	Physical capability	Physiotherapeutic rehabilitation to increase the patient's capability to perform physical activities i.e. specific exercises to reduce stiffness and pain
	Psychological capability	Using cognitive behavioural techniques to increase self and task efficacy beliefs.
Opportunity	Physical opportunity	Identifying and developing problem solving techniques to overcome physical barriers to physical activity i.e. walking to a bus stop further away from the house.
	Social opportunity	Fostering solutions of how to perform physical activities in a social context i.e. communal gardening.
Motivation	Reflective	Using the PEP-TALK discussions to consciously weigh up the individual's pros and cons to performing more physical activity.
	Automatic	Developing active participation from the PEP-TALK participants to encourage linking physical activity into their daily life routine behaviours. Repetition of physically active behaviours can then become linked to everyday activities and will hopefully form into healthy habits which consistently remind, prompt and foster long-term motivation to increase physical activity.

A large proportion of the research into behaviour change techniques to increase physical activity in older adults is based on short-term (less than 12-month follow-up) data. By combining this well-developed model of intervention development, with the SCT model, and specific cognitive behavioural techniques which we have used successfully in previous interventions to increase physical activity (27-28), we hope to produce a sustained behaviour change.

Acceptability of the intervention

The evidence repeatedly recommends listening to what participants want from the intervention (20, 22-23). We aim to learn from participants what their motivations are and what will make the intervention acceptable (22).

We aim to integrate the four analytical themes from the systematic review (9) into the intervention development:

(1) Psychoeducation
(2) Reducing fear-avoidant behaviours in response to unhelpful beliefs i.e. "physical activity will damage my joint replacement"
(3) Providing opportunities for personal enjoyment of the physical activity.
(4) Enabling social contact, peer-support and advice from previous patients

To enhance the acceptability of the intervention, the social enjoyment of the group will be encouraged for making friends, as this is highly valued in older adults. Another aspect is the individual variation in the intervention exercises. This will be overcome by providing one-to-one attention, going at the participant's own pace and making the credibility of the physiotherapist and the intervention content explicit to meet the expectations and needs of older adults.

Hypothesised mediation pathway

From the literature and from our previous models of behaviour change to increase physical activity combined with physiotherapy interventions (27-28), we have developed a model of mediation. We propose that our intervention will increase physical activity levels by increasing self-efficacy and reducing fear avoidance. The pathway of mediation is outlined in the figure below. We are not specifically targeting mental health or pain experience with our intervention, but we are sensitive to monitor if increasing physical activity has a positive effect on these variables.

Proposed pathway of mediation for the PEP-TALK programme

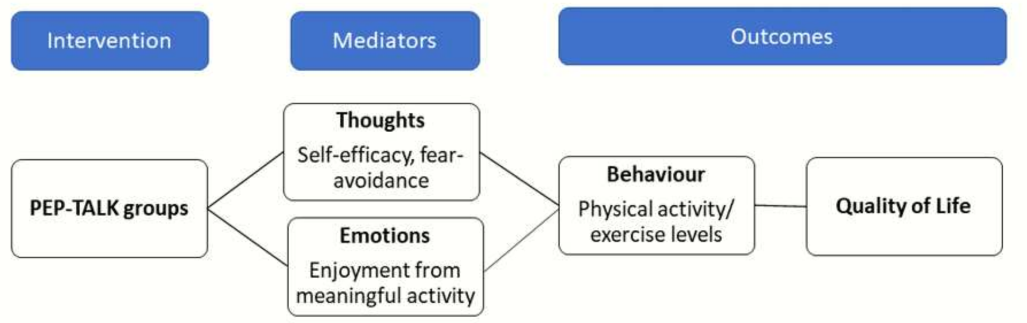

Figure 2: Proposed mediation pathway for PEP-TALK

The PEP-TALK intervention

The PEP-TALK behaviour change group will be delivered face-to-face by one physiotherapist to a group for 30 minutes. Immediately after finishing the 'talking' session the participants will begin their THR/TKR rehabilitation exercises for another 30 minutes. During the exercise session the physiotherapist will continue to talk to the participants. Asking them what they are thinking/feeling when they perform the exercises; encouraging them to reflect on their experience of pain if they encounter this. Using reflective questions to help the participants solve any barriers they encounter whilst performing the exercises. These informal encounters are used to put the theory discussed in the 'talking' group into real life practice.

At the beginning of the PRP-TALK course, intervention participants receive a printed workbook which includes information summarising the techniques, sharing examples and includes homework tasks. The homework tasks are essential for participants to practice translating the behaviour change techniques discussed in the groups, into their real lives, Reflecting on their experiences, thoughts, feelings and behaviours.

The PEP-TALK intervention, in total, lasts for one hour. The control participants only attend the THR/TKR rehabilitation exercise class, which lasts 30 minutes. The control THR/TKR exercise class includes the same physical exercises as prescribed in the intervention group's exercise class but without any of the behaviour change discussion.

Methods of Delivery

The PEP-TALK sessions will be delivered by a physiotherapist trained in the PEP-TALK intervention. The training consists of the PEP-TALK manual outlining the theories of behaviour change, principles of the cognitive behavioural approach, the identified barriers and facilitators to physical activity and exercises. Following this, physiotherapists will attend a one-day training session delivered by a member of the PEP-TALK programme development team (BF, ZH, TS). In this, physiotherapists will discuss the theoretical underpinning of the programme and be provided with case studies and

examples of how the PEP-TALK intervention is designed to be prescribed, and discussion on potential threats to fidelity. We will role play some patient-physiotherapist interactions to provide practical experiences of the intervention in a supportive environment. The trainers will assess how well physiotherapists follow the intervention and will acknowledge any deviations to correct practice.

The PEP-TALK intervention is delivered immediately prior to an exercise group. By timing the interventions with the group discussion first, participants will immediately action and re-enforce the encouragement for physical activity participations through exercising. We have stipulated a maximum PEP-Talk group size of 12 participants to prevent participants from becoming lost in the group and to parallel the standard usual care group size.

A group rather than a one-to-one approach has the advantage of enabling collaborative and vicarious learning, which can improve self-efficacy regarding their goal behaviour (i.e. increased physical activity), whilst also providing lower unit-costs of delivery (29). The principles underpinning this derive from Bandura et al's (17) SCT regarding vicarious learning where learning is proposed to not be acquired through direct experience but by observing other people's actions and consequences (modelling). Secondly, the principles of social cognitive development theory (30) are adopted where knowledge is acquired through guided collaboration with people who already have the knowledge. Collaborative learning with 'peers' and expert people (facilitators) helps bridge distance between an individual's level of skill and their potential, the 'zone of proximal development' (30).

Participants and physiotherapists will be encouraged to develop a positive therapeutic alliance where the physiotherapist will generate an environment of trust and belief around the individual challenges the patient has and to support them to overcome these for sustained physical activity adoption. Evidence has highlighted the beneficial impact of a positive therapeutic alliance on outcomes within physiotherapy practice (31). Due to the nature of identifying individual's helpful and unhelpful thoughts, barriers and facilitators and strategies, the intervention has flexibility in the intention to support this approach. Therefore, whilst the intervention described below has key set-elements which form the content of sessions, there will be opportunity for individuals to express meaningful thoughts and experiences to them, thereby personalising the intervention.

Where Delivered

The PEP-TALK behaviour change group and subsequent exercise sessions will be delivered in an out-patient physiotherapy gym environment. Participants will be sat in a circle to facilitate dialogue. Following the 'talking' intervention, participants begin their THR/TKR exercise session. They will perform exercises in exercise stations, monitored by a trained physiotherapist.

The PEP-TALK behaviour change programme consists of six sessions (A-F) delivered as a rolling programme. Once a new participant has been randomised they can join the groups in any session: A, B, C, D, E or F. Consequently, in every session delivered there will be a mixture of participants who have attended 5,4,3,2,1 or 0 previous PEP-TALK sessions. This necessitates a large amount of repetition of the aims and techniques in every session to ensure all members of the group understand the core behaviour change messages. The rolling programme also enables groups to run continuously, minimising a participant's waiting time to join a group.

A treatment log will be completed by the physiotherapists to record the component of what is discussed across the participants group in each of the session.

Group session will be re-enforced with a participant workbook. This provides participants with salient information from each session, and provides them with exercise progressions, an exercise diary, a guide and space to complete homework tasks/record.

Content of PEP-TALK Sessions

Each of the six PEP-TALK sessions (A - F) will follow this structure:

(1) agenda setting – what will be covered in the session
(2) today's session – covering topics which have been demonstrated to impact on physical activity following joint replacement (content listed below)
(3) conclusion – provision of homework and summarising topics covered today and what will be covered in the next session
(4) break - before commencing exercises group session

There is a degree of overlap between sessions to aid reinforcement of ideas and beliefs. This overlap is largely on identification of barriers and discussion of progress for individuals to share. The principles around the six sessions are presented below:

1. <u>"Being Physically Active": Individual's meaning of physical activity and barriers and problem-solving</u>
   a. Exploring what physical activity means to each participant. For example: active living, transport, sports and exercise. Consideration by participants of what proportion of their lives are engaged with each aspect of physical activity and what the harms and benefits are of being inactive and active. Participants consider what potential barriers exists to activity and whether they want to address these barriers.

2. <u>"Gradually increasing physical activity": Under/Over-Activity, Pacing, Graded Activities</u>
   a. In this session individuals will be taught the principles of pacing and graded-activity. Discussion will be centred on an example e.g. cleaning the car and how pacing and graded-activity could be implemented. The concept of determining a 'baseline' of activity will be established. Individuals will be asked to consider what challenges they have to implementing a graded-activity programme in everyday activities. To facilitate this, individuals will be asked to consider another activity and work through how that activity may be paced in the following week.

3. <u>"Should I be doing this?" : Fear-avoidance</u>
   a. This session will focus on education on avoidance of activity and why individuals avoid activities in relation to their recovery and protection of a joint replacement. Consideration will be focused on thoughts which could be challenged particularly in relation to functional tasks such as washing and dressing, walking, sports or home activities. Individuals will consider how fear avoidance is a circular behaviour in relation to 'thoughts', 'feelings', 'actions', 'results' which can reinforce health beliefs around activity avoidance but acknowledging that such a cycle is a normal response given their previous pain. Discussion will be made for individuals to consider how they may overcome these beliefs.

4. <u>"Physical activity benefits" : Emotion and Sleep, Exercise, Social links</u>
   a. Exploration on the benefits of physical activity on emotional health and sleep will form the basis of this session. Individuals will be asked to consider how being less depressed, stressed and sleep deprived and happier with greater social contact can affect their lives. They will consider how these factors inhibit their ability to be more physically active. Discussion will be made on how worry may relate to pain and what strategies they must address this. Individuals will also think about challenging beliefs around failure to be able to complete certain activities and what their own fears are regarding being more or less active.

5. <u>"Can I change how I think?": Worry, Distraction, Unhelpful Thoughts</u>

a. Fears and worries about jeopardizing recovery and long-term joint health will be explored in this session. Individuals will identify and challenge beliefs around physical activity and harm or damage which are unhelpful thoughts. They will explore a 'vicious cycle' notion where unhelpful thinking leads to feeling low, leading to feeling unmotivated, leading to reduced physical activity leading to atrophy which reinforces the unhelpful thought. Individuals will be asked to consider 'answer back thoughts' and strategies to address such unhelpful thoughts and distractions.

6. "Staying active and having fun" : Social and Rewarding
    a. The benefits of physical activity as a reward will be explored in this session. They will be asked to consider what activities they do alone, and which could be done with others, to increase social contact and increase motivation and pleasure from participating in an activity. Individuals will consider potential barriers and strategies to promote and adopt such an approach to everyday activities' which interest them.

Homework Activities

Participants will be supported with skills developed in the group, to work at home on challenges, barriers and facilitators to physical activity behaviour. The 'home-work' after each session will include pacing and behaviour modification, goal-setting to the individual's health and social needs, and techniques to challenge fear avoidant behaviours.

Follow-up Telephone Calls

Three follow-up telephone calls (maximum 20-minute duration) will be undertaken at two, four and six weeks following the last group session. Follow-up telephone calls are an important element of the behaviour change intervention. They will review participant's goals, identifying any barriers to the completion of these goals, and review any 'helpful' and 'unhelpful' thoughts or feelings towards physical activity which may have arisen since the last consultation. Each telephone call will close with the development of longer-term physical activity plans and promotion of empowerment towards physical activity participation using these behavioural principles instilled during the group intervention.

Adherence and Fidelity

The PEP-TALK team phone the physiotherapist delivering the intervention group after their first session has been delivered. The aim of this call is to address any problems the physiotherapist may have encountered and for the PEP-TALK team to offer solutions and tips. After the third session has been delivered, a member of the PEP-TALK team visit the site and observe a PEP-TALK behaviour change and exercise session to perform a quality assessment (QA). If there are quality concerns, then the site will receive additional training and another QA visit will be undertaken.

At a participant level, compliance to the PEP-TALK intervention will be arbitrarily met with participants required to attend 70% of the behaviour-change and exercise groups and 66% of the telephone calls.

Access to the Intervention

The PEP-TALK intervention manual and work-book will be available on completion of the trial. This can be accessed through the corresponding author.

Conclusions

The development and content of the PEP-TALK intervention has been presented. This addresses key modifiable risk factors to physical inactivity following hip and knee replacement. The effectiveness of

this intervention will now be assessed in the multi-centre, pragmatic, randomised controlled trial (PEP-TALK Trial).

SUPPLEMENTARY FILE 1: REFERENCES

1. National Joint Registry. 2018. Reports Online 2018. National Joint Registry UK. Accessed: 22 July 2019. Available at: http://www.njrcentre.org.uk/njrcentre/Healthcare-providers/Accessing-the-data/ReportsOnline
2. Dailiana ZH, Papakostidou I, Varitimidis S, Liaropoulos L, Zintzaras E, Karachalios T, Michelinakis E, Malizos KN. Patient-reported quality of life after primary major joint arthroplasty: a prospective comparison of hip and knee arthroplasty. BMC Musculoskelet Disord. 2015;16:366.
3. Papakostidou I, Dailiana ZH, Papapolychroniou T, Liaropoulos L, Zintzaras E, Karachalios TS, Malizos KN. Factors affecting the quality of life after total knee arthroplasties: a prospective study. BMC Musculoskelet Disord. 2012;13:116.
4. Inacio MC, Pratt NL, Roughead EE, Graves SE. Comparing co-morbidities in total joint arthroplasty patients using the RxRisk-V, Elixhauser, and Charlson Measures: a cross-sectional evaluation. BMC Musculoskelet Disord. 2015;16:385.
5. Peter WF, Dekker J, Tilbury C, Tordoir RL, Verdegaal SH, Onstenk R, Bénard MR, Vehmeijer SB, Fiocco M, Vermeulen HM, van der Linden-van der Zwaag HM, Nelissen RG, Vliet Vlieland TP. The association between comorbidities and pain, physical function and quality of life following hip and knee arthroplasty. Rheumatol Int. 2015;35:1233-41.
6. Martinez-Cano JP, Herrera-Escobar JP, Arango Gutierrez AS, Sanchez Vergel A, Martinez-Rondanelli A. Prospective quality of life assessment after hip and knee arthroplasty: short- and mid-term follow-up results. Arthroplast Today. 2016;3:125-130.
7. Sampson UK, Fowkes FG, McDermott MM, Criqui MH, Aboyans V, Norman PE, Forouzanfar MH, Naghavi M, Song Y, Harrell FE Jr, Denenberg JO, Mensah GA, Ezzati M, Murray C. Smith E, Hoy DG, Cross M, Vos T, Naghavi M, Buchbinder R, Woolf AD, March L. PICO. The global burden of other musculoskeletal disorders: estimates from the Global Burden of Disease 2010 study. Ann Rheum Dis. 2014;73:1462-9.
8. Withers TM, Lister S, Sackley C, Clark A, Smith TO. Is there a difference in physical activity levels in patients before and up to one year after unilateral total hip replacement? A systematic review and meta-analysis. Clin Rehabil. 2017;31:639-650.
9. Smith TO, Latham S, Maskrey V, Blyth A. Patients' perceptions of physical activity before and after joint replacement: a systematic review with meta-ethnographic analysis. Postgrad Med J. 2015;91:483-91.
10. Global Burden of Disease Study 2013 Collaborators. Global, regional, and national incidence, prevalence, and years lived with disability for 301 acute and chronic diseases and injuries in 188 countries, 1990–2013: a systematic analysis for the Global Burden of Disease Study 2013. Lancet. 2015;386:743–800
11. Lee CD, Folsom AR, Blair SN. Physical activity and stroke risk: a meta-analysis. Stroke. 2003;34:2475-81.
12. Laaksonen DE, Lindström J, Lakka TA, Eriksson JG, Niskanen L, Wikström K, et al; Finnish diabetes prevention study. Physical activity in the prevention of type 2 diabetes: the Finnish diabetes prevention study. Diabetes. 2005;54:158-65.
13. Lee IM. Physical activity and cancer prevention--data from epidemiologic studies. Med Sci Sports Exerc. 2003;35:1823-7.
14. Nocon M, Hiemann T, Müller-Riemenschneider F, Thalau F, Roll S, Willich SN. Association of physical activity with all-cause and cardiovascular mortality: a systematic review and meta-analysis. Eur J Cardiovasc Prev Rehabil. 2008;15:239-46.

15. Abraham C and Michie, S. A taxonomy of behaviour change techniques used in interventions. Health Psychology, 2008;27:379-87.
16. Chase J. A. Interventions to increase physical activity among older adults: a meta-analysis. The Gerontologist, 2015;55:706-18.
17. Bandura A. Social foundations of thought and action: A social cognitive theory. Prentice-Hall, Inc, 1986.
18. Baranowski T, Anderson C and Carmack C. Mediating variable framework in physical activity intervetnions: How are we doing? how might we do better? Am J Prevent Med. 1998;15;266-97.
19. Arnautovska U, O'Callaghan F, Hamilton K. Applying the Integrated Behavior Change Model to Understanding Physical Activity Among Older Adults: A Qualitative Study. J Sport Exerc Psychol. 2017;39:43-55.
20. Olanrewaju O, Kelly S, Cowan A, Brayne C, Lafortune L. Physical activity in community dwelling older people: a systematic review of reviews of interventions and context. PLoS One. 2016;11:e0168614.
21. Withers TM, Lister S, Sackley C, Clark A, Smith TO. Is there a difference in physical activity levels in patients before and up to one year after unilateral total hip replacement? A systematic review and meta-analysis. Clin Rehabil. 2017;31:639-50.
22. Devereux-Fitzgerald A, Powell R, Dewhurst A, French DP. The acceptability of physical activity interventions to older adults: A systematic review and meta-synthesis. Soc Sci Med. 2016;158:14-23.
23. French DP, Olander EK, Chisholm A, Mc Sharry J. Which behaviour change techniques are most effective at increasing older adults' self-efficacy and physical activity behaviour? A systematic review. Ann Behav Med. 2014;48:225-34.
24. Pekmezi DW, Neighbors CJ, Lee CS, Gans KM, Bock BC, Morrow KM, Marquez B, Dunsiger S, Marcus BH. A culturally adapted physical activity intervention for Latinas: a randomized controlled trial. Am J Prev Med. 2009;37:495-500.
25. Carstensen LL. Motivation for social contact across the life span: a theory of socioemotional selectivity. Nebr Symp Motiv. 1992;40:209-54.
26. Michie S, Atkins L, West R. The Behaviour Change Wheel: A guide to designing Interventions. Silverback Publishing. 2014
27. Lamb SE, Lall R, Hansen Z, Castelnuovo E, Withers EJ, Nichols V, Griffiths F, Potter R, Szczepura A, Underwood M; BeST trial group. A multicentred randomised controlled trial of a primary care-based cognitive behavioural programme for low back pain. The Back Skills Training (BeST) trial. Health Technol Assess. 2010;14:1-253.
28. Lamb 2015. Better outcomes for older people with spinal trouble. Accessed: 24 May 2018. Available at: http://www.isrctn.com/ISRCTN12698674
29. Critchley DJ, Ratcliffe J, Noonan S, Jones RH, Hurley MV. Effectiveness and cost-effectiveness of three types of physiotherapy used to reduce chronic low back pain disability: a pragmatic randomized trial with economic evaluation. Spine 2007;32:1474–81.
30. Vygotsky L. Interaction Between. Learning and Development. In Gauvain and Cole (eds) Readings on the Development of Children. New York: Scientific. American Books. pp. 34-40, 1978.
31. Paulo H. Ferreira, Manuela L. Ferreira, Christopher G. Maher, Kathryn M. Refshauge, Jane Latimer, Roger D. Adams; The therapeutic alliance between clinicians and patients predicts outcome in chronic low back pain. Phys Ther 2013;93:470–8.

Supplementary File 2: Additional results

Pre-Specified Definition of Compliance

Compliance was defined in three nested levels for both randomised groups. These are:

Strict Compliance (as defined in the original Protocol):
Usual Care group
• Attends at least 4 out of 6 physiotherapy sessions

Experimental Intervention group
• Attends at least 4 out of 6 group intervention sessions with a minimum of 3 participants per session
• Received 2 out of 3 follow-up telephone calls

Compliance:
Usual Care group
• Attends at least 4 out of 6 physiotherapy sessions

Experimental Intervention group
• Attends at least 4 out of 6 group intervention sessions with a minimum of 3 participants per session

Attendance:
Usual Care group
• Attends at least 1 out of 6 physiotherapy sessions

Experimental Intervention group

- Attends at least 4 out of 6 group intervention sessions.

Additional Results

A summary of withdrawals is provided in **Supplementary Table 1**.

The primary analysis is performed assuming the data is missing at random (MAR). To assess the MAR assumption, varying scores of the UCLA Activity Score for all time points were imputed where data is missing and these “complete” datasets were reanalysed, using the same mixed effects as used in the primary analysis. For each missing data point, the median value of the group that participant belongs to is imputed and the imputed dataset analysed. The analysis is repeated on a population that has the 60th quantile imputed for one group’s missing values and the 40th quantile for the other, then again using the 70th and 30th quantiles, up to 90th and 10th quantiles. The process was repeated but flipping the groups. In total nine sensitivity analyses were performed and the results displayed graphically in **Supplementary Figure 5**. This method used simple imputation of these quantiles, therefore the estimates of the variance will be effected, and so will all p-values and Confidence Intervals reported. **Supplementary Figure 5** shows that there would need to be an implausibility large departure from the missing at random assumption to see a statistically significant result in either direction with a result only being yielded if the 10th and 90th percentiles are imputed into each treatment group. This suggests the result from the primary analysis is robust to missing data and adds support to the findings from the primary analysis.

A sensitivity analysis on the per-protocol population has been performed to assess the internal validity of the trial's primary results. The analysis is based on the same mixed effects analysis model as used for the primary outcome but for the Per-Protocol population as described in the Statistical Analysis Plan.[35] To be considered per-protocol participants must have data on the UCLA Activity Score at 12 months, cannot be "Non-Compliant", cannot be part of the COVID-19 group (as these participants did not complete their intervention per-protocol), did not crossover randomised treatments and did not have any Important protocol deviations reported. Results from this analysis are reported in **Supplementary Table 3**. The per-protocol analysis reinforces the main trial result findings, there is no between group difference.

An analysis on the primary outcome using a reduced version of the primary analysis model, only using person as a random effect has been performed. The results are presented in **Supplementary Table 4**. The results from the reduced model in **Supplementary Table 4** are extremely similar the primary analysis results. The Akaike Information Criterion (AIC) for the primary analysis model was 1,372.47 whereas the AIC for the reduced model was 1,370.84 suggesting a marginally better model fit with centre removed.

All subgroup analyses are on the primary outcome only. Subgroup analyses of the two clinical stratifying variables (type of operation and (THR or TKR), Charlson Comorbidity Index Score (1–3 or ≥ 4)) were performed as well as a subgroup analysis on COVID-19 status (Pre-COVID-19 or COVID-19). These used an extended primary analysis model including an interaction term between treatment and each stratifying variable/COVID-19 status to define the subgroups. These analyses are exploratory, and results should be interpreted with due caution. The results will be presented in a **Supplementary Figure 6**.

Supplementary Figure 1 gives a plot of complication type.

Descriptive statistics for the Generalized Self-Efficacy Scale, Tampa Scale for Kinesiophobia, Hospital Anxiety and Depression Score, EQ-5D-5L Index, EQ-VAS and Numerical Rating Scale for Pain are given by COVID-19 status in **Supplementary Table 5**, no formal analysis is performed. The presentation of these results was pre-specified in the analysis plan and aid in assessing the impact of the COVID-19 pandemic on the trial participants. Results indicate potentially higher levels of anxiety, depression and kinesiophobia at six-months in the COVID-19 population, these apparent differences were not sustained to the 12-month follow-up. Observed self-efficacy scores were lower in the COVID-19 group across all follow-up time points. Other measures did not indicate any noticeable between group difference. These results should be interpreted with great caution due to small sample size, non-random groups, and the exploratory nature of the results.

Supplementary Table 1: Withdrawals summary

	Usual (n=13)	Experimental (n=24)	Total (n=37)
**Treatment Non-Compliance Reason**			
Complete withdrawal from the study and use of data	2	2	4
Withdrawal from intervention and completion of questionnaires	4	11	15
Withdrawal from intervention only	7	11	18
**Withdrawal Time Point**			
6 Months	12	17	29
12 Months	1	7	8

N - number of participants

Supplementary Table 2: Questionnaire returns by treatment group

Time Point	Usual	Experimental	Cumulative missing data	Total with data
Baseline	85 (100.0)	139 (100.0)	0 (0.0)	224 (100.0)
6 Months	69 (81.2)	117 (84.2)	38 (17.0)	186 (83.0)
12 Months	70 (82.4)	112 (80.6)	42 (18.8)	182 (81.2)

All data frequency and (%)

Supplementary Table 3: UCLA Activity Score per-protocol results

Time Point	Usual	Experimental	Mean Difference	
	n, Mean (SD)	n, Mean (SD)	Unadjusted	Adjusted (95% CI)
Baseline	n=46, 3.76 (1.51)	n=54, 3.67 (1.65)	-0.09	
6 Months	n=44, 4.91 (1.44)	n=50, 5.18 (1.86)	0.27	0.43 (-0.23,1.08)
12 Months	n=46, 5.04 (1.59)	n=54, 4.83 (1.79)	-0.21	-0.17 (-0.81,0.48)

CI - confidence intervals; N – number of participants; SD – standard deviation

Supplementary Table 4: UCLA Activity Score reduced model (no recruiting centre random effect) results

Time Point	Usual	Experimental	Mean Difference	
	n, Mean (SD)	n, Mean (SD)	Unadjusted	Adjusted (95% CI)
Baseline	n=85, 3.62 (1.52)	n=138, 3.57 (1.57)	-0.06	
6 Months	n=69, 4.77 (1.52)	n=117, 4.97 (1.68)	0.20	0.28 (-0.21,0.76)
12 Months	n=70, 4.87 (1.61)	n=111, 4.84 (1.91)	-0.03	-0.03 (-0.52,0.46)

CI - confidence intervals; N – number of participants; SD – standard deviation

Supplementary Table 5: Descriptive results for selected secondary outcomes by COVID-19 status

	Pre-COVID-19	COVID-19
	**n, Mean (SD)**	**n, Mean (SD)**
**Generalized Self-Efficacy Scale**		
Baseline	n=153, 31.82 (5.49)	n=69, 30.90 (5.24)
6 Months	n=112, 33.04 (5.22)	n=44, 31.50 (5.29)
12 Months	n=112, 32.83 (6.27)	n=50, 30.74 (6.13)
**Tampa Scale for Kinesiophobia**		
Baseline	n=153, 40.09 (7.81)	n=68, 39.38 (7.20)
6 Months	n=103, 34.86 (7.79)	n=44, 35.82 (6.62)
12 Months	n=103, 35.57 (8.30)	n=44, 35.80 (6.50)
**Hospital Anxiety and Depression Scale (Overall)**		
Baseline	n=154, 11.99 (6.38)	n=69, 12.83 (7.46)
6 Months	n=110, 8.65 (6.20)	n=46, 9.39 (6.89)
12 Months	n=113, 9.46 (6.95)	n=47, 9.38 (6.60)
**Hospital Anxiety and Depression Scale (Anxiety)**		
Baseline	n=154, 6.19 (3.84)	n=69, 6.71 (4.24)
6 Months	n=112, 4.79 (3.55)	n=46, 5.33 (4.16)
12 Months	n=113, 5.11 (3.75)	n=48, 5.40 (3.95)
**Hospital Anxiety and Depression Scale (Depression)**		
Baseline	n=155, 5.83 (3.40)	n=69, 6.12 (3.95)
6 Months	n=113, 3.89 (3.31)	n=47, 4.09 (3.66)
12 Months	n=115, 4.30 (3.97)	n=48, 4.23 (3.44)
**EQ-5D-5L Index**		
Baseline	n=155, 0.40 (0.24)	n=69, 0.38 (0.28)
6 Months	n=129, 0.68 (0.25)	n=56, 0.69 (0.23)
12 Months	n=128, 0.67 (0.26)	n=55, 0.68 (0.29)
**EQ-VAS**		
Baseline	n=155, 62.34 (21.77)	n=69, 57.55 (23.07)
6 Months	n=130, 71.84 (20.74)	n=55, 75.02 (16.28)
12 Months	n=124, 73.19 (19.85)	n=55, 71.82 (17.62)
**Numerical Rating Scale for Pain**		
Baseline	n=155, 7.09 (1.87)	n=69, 7.10 (1.82)
6 Months	n=115, 3.55 (2.72)	n=47, 3.28 (2.59)
12 Months	n=112, 3.68 (2.88)	n=51, 3.47 (2.87)

N – number of participants; SD – standard deviation

Supplementary Figure 1: Complication type by randomised group

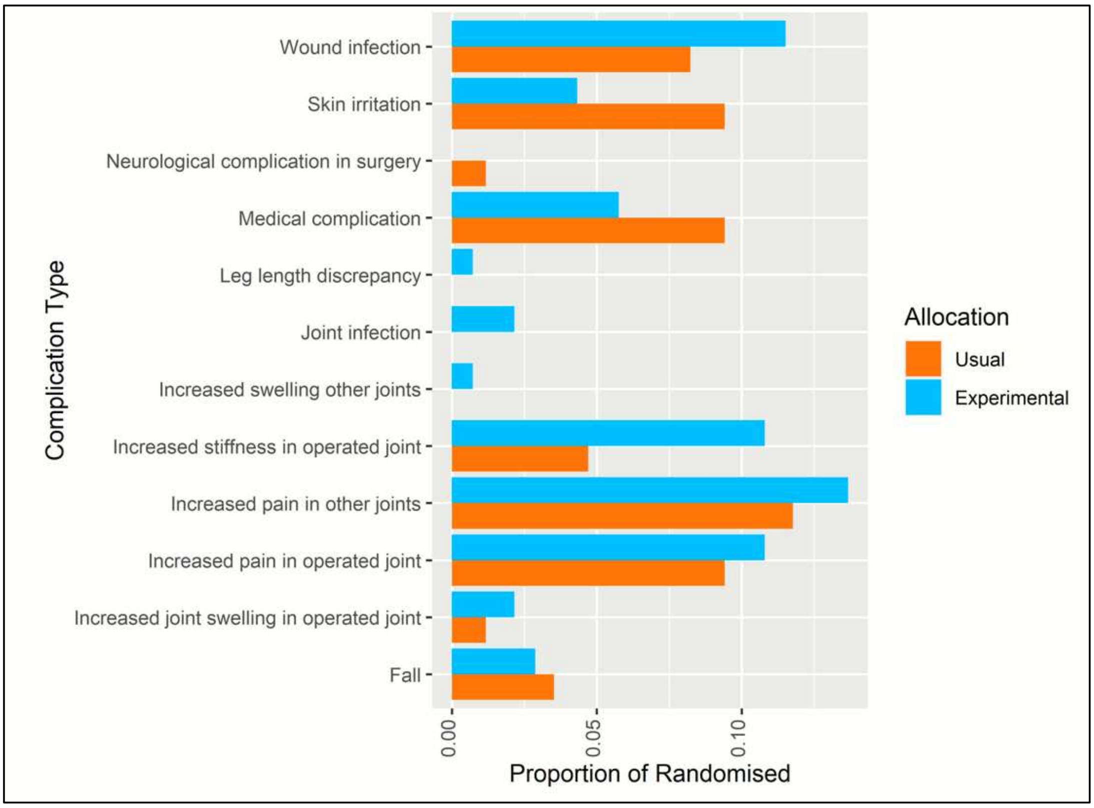

Supplementary Figure 2: Overall compliance by (a) raw frequencies and (b) percentage of randomised group

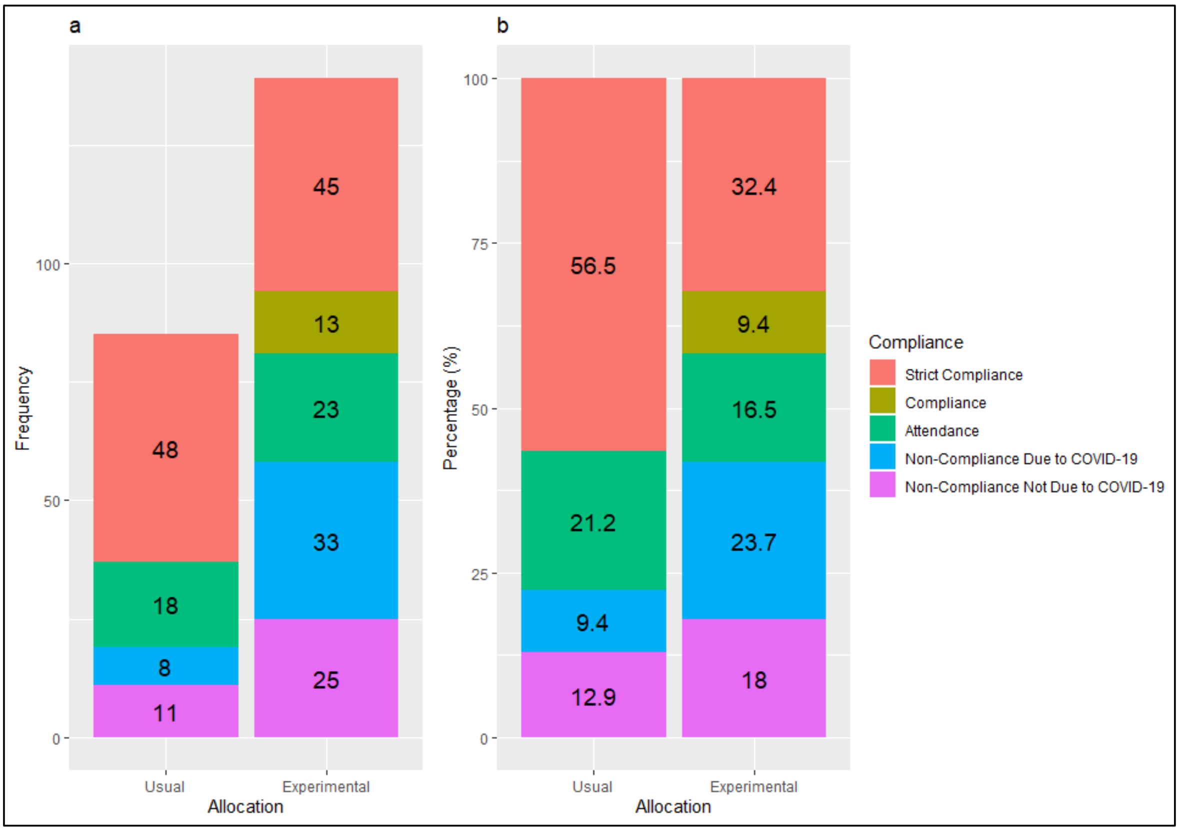

Supplementary Figure 3: Experimental intervention group sizes over time, including change from a randomisation ratio of 1:1 to 2:1

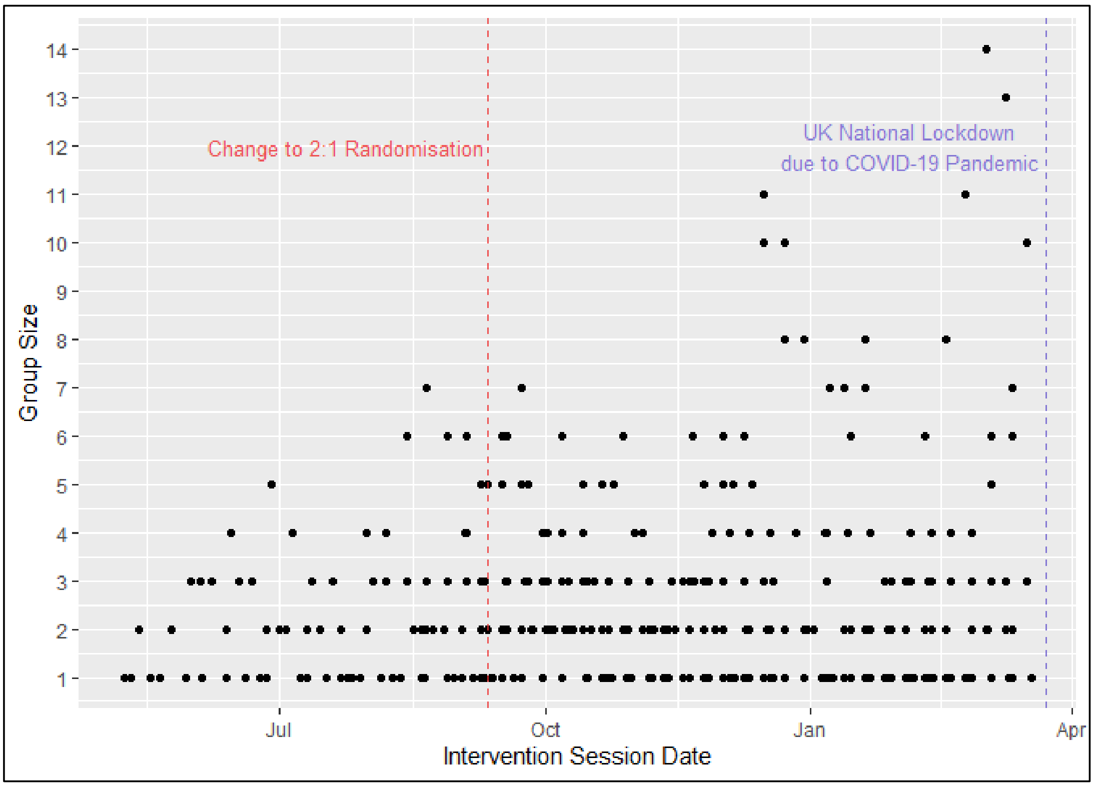

Supplementary Figure 4: Experimental intervention group compliance by COVID-19 group

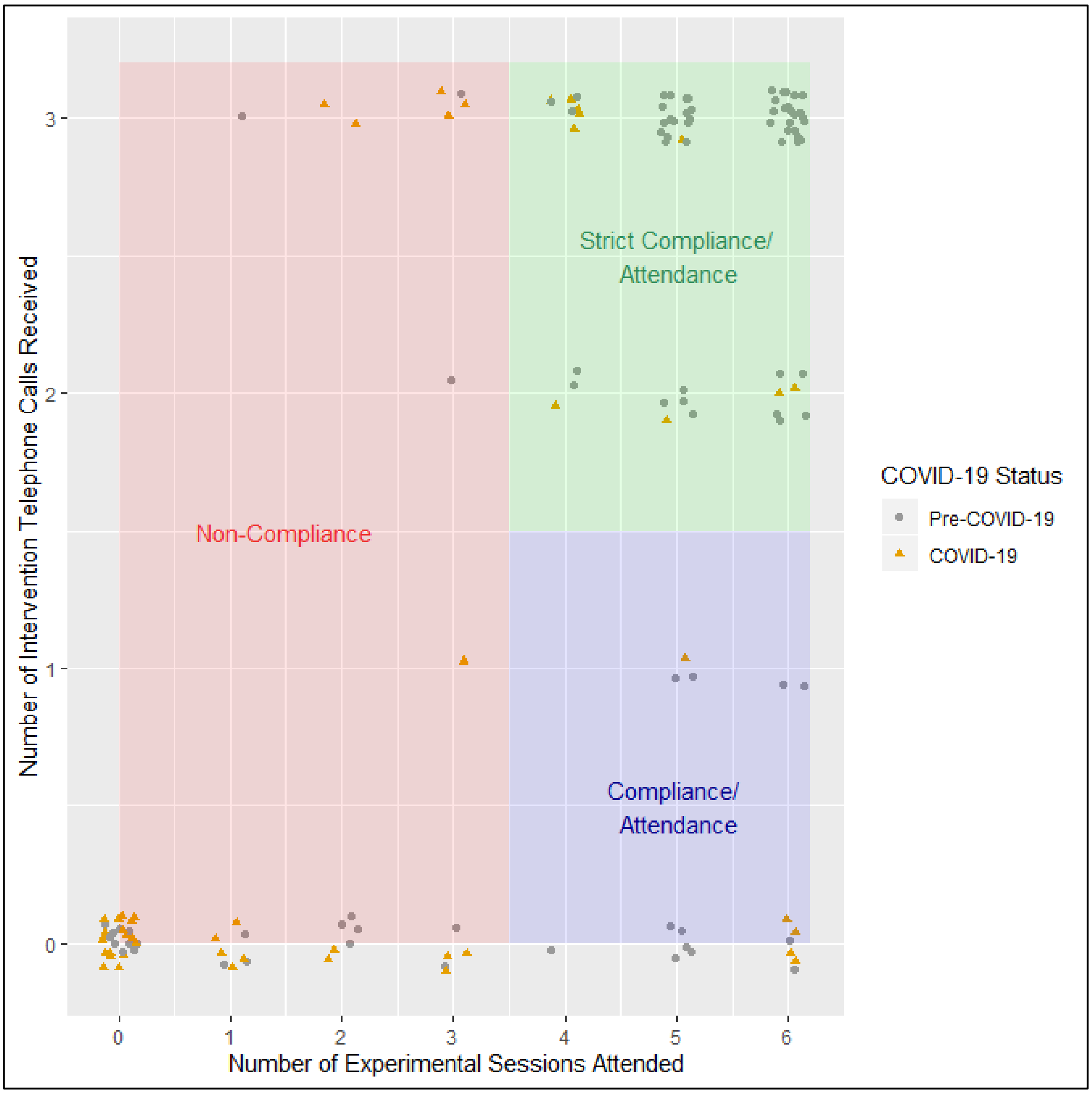

Supplementary Figure 5: 12 month adjusted mean difference UCLA Activity Score for varying imputed quantiles for missing data

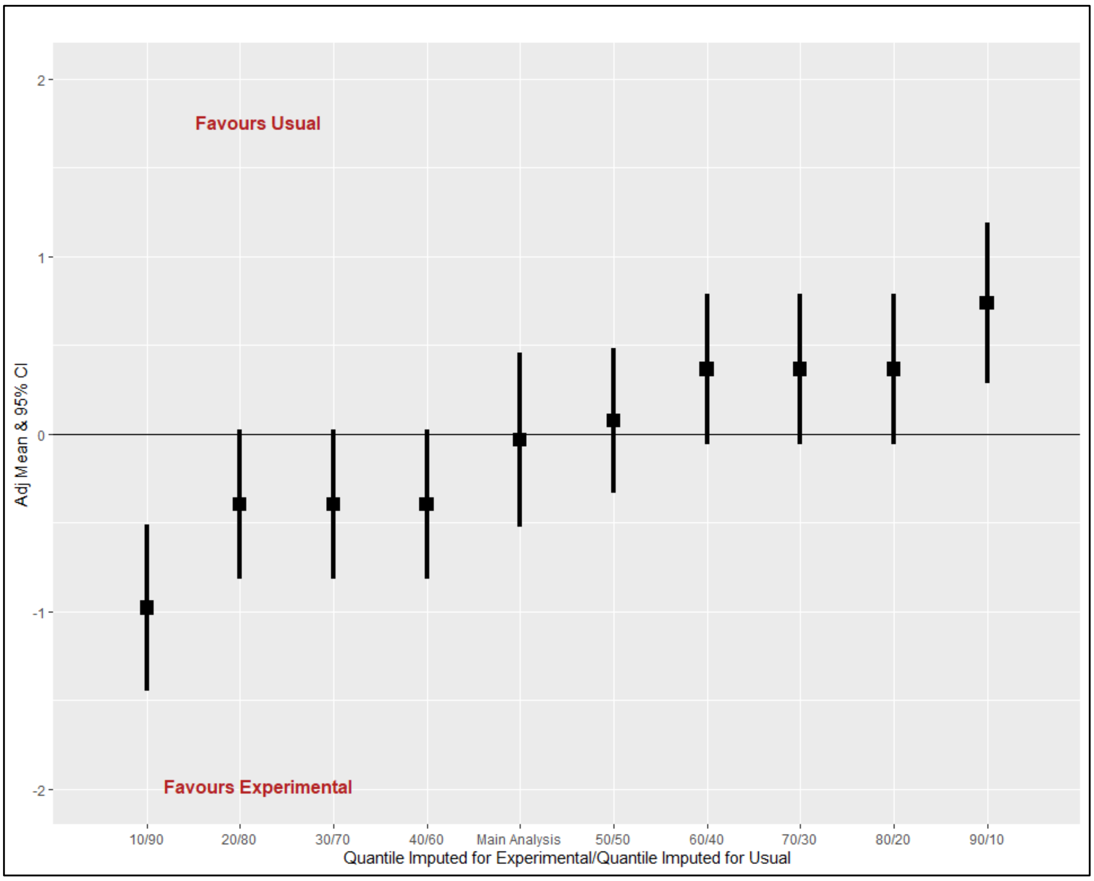

Supplementary Figure 6: Subgroup analyses results

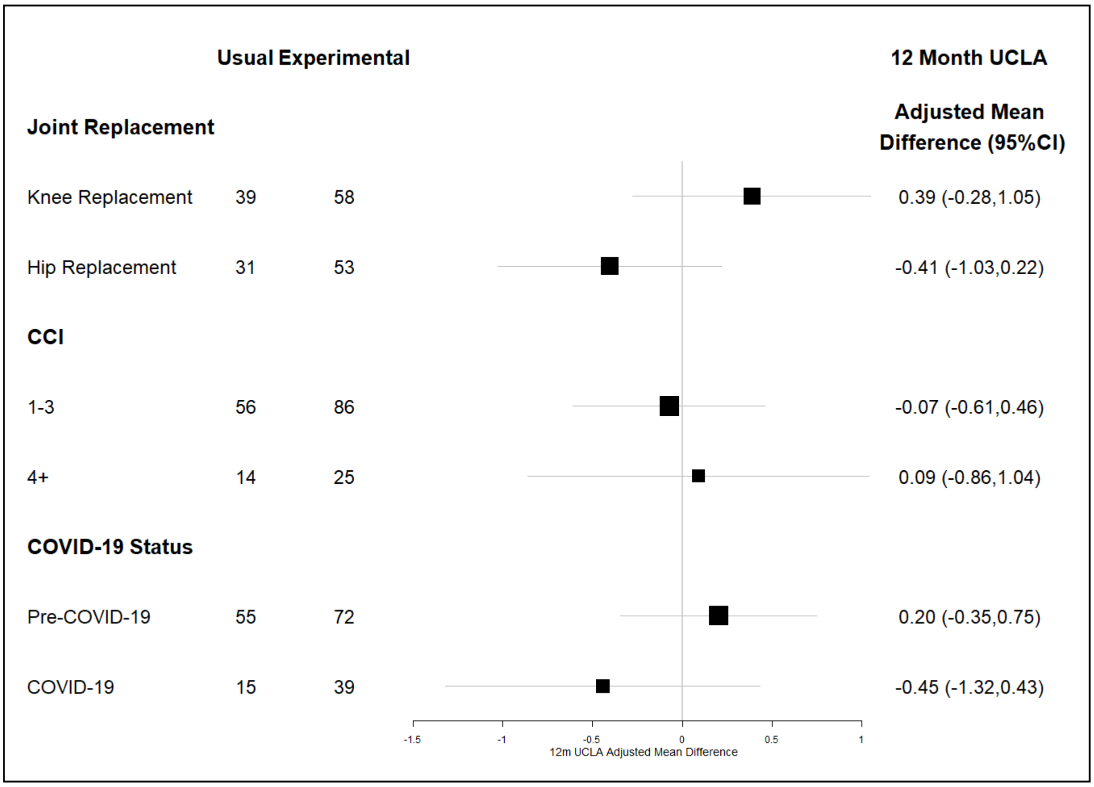

CCI – Charlson Comorbidity Index; CI – Confidence Intervals; UCLA – University for Los Angeles Activity Score
