# Supplementary material for: Randomised controlled trial of a behaviour change physiotherapy intervention to increase physical activity following hip and knee replacement: the PEP-TALK trial
Source: BMJ Open. 2022 May 31;12(5):e061373. doi: 10.1136/bmjopen-2022-061373 (PMC9157340; doi:10.1136/bmjopen-2022-061373)
Supplement: Author's manuscript [file bmjopen-2022-061373.draft_revisions.pdf]

BMJ Open is committed to open peer review. As part of this commitment we make the peer review history of every article we publish publicly available.

When an article is published we post the peer reviewers' comments and the authors' responses online. We also post the versions of the paper that were used during peer review. These are the versions that the peer review comments apply to.

The versions of the paper that follow are the versions that were submitted during the peer review process. They are not the versions of record or the final published versions. They should not be cited or distributed as the published version of this manuscript.

BMJ Open is an open access journal and the full, final, typeset and author-corrected version of record of the manuscript is available on our site with no access controls, subscription charges or pay-per-view fees (http://bmjopen.bmj.com).

If you have any questions on BMJ Open's open peer review process please email info.bmjopen@bmj.com

BMJ Open

Behaviour change physiotherapy intervention to increase physical activity following hip and knee replacement: the PEP-TALK randomised controlled trial

Journal:	*BMJ Open*
Manuscript ID	bmjopen-2022-061373
Article Type:	Original research
Date Submitted by the Author:	25-Jan-2022
Complete List of Authors:	Smith, Toby O.; University of Oxford, Nuffield Department of Orthopaedics, Rheumatology and Musculoskeletal Sciences; University of East Anglia,<br>Parsons, Scott; University of Oxford, Nuffield Department of Orthopaedics, Rheumatology and Musculoskeletal Disorders<br>Ooms, Alexander; University of Oxford, Nuffield Department of Orthopaedics, Rheumatology and Musculoskeletal Disorders<br>Dutton, Susan; University of Oxford, CSM<br>Fordham, Beth; University of Oxford, NDORMS<br>Garrett, Angela; University of Oxford, Nuffield Department of Orthopaedics, Rheumatology and Musculoskeletal Sciences<br>Hing, Caroline; St George's University Hospitals NHS Foundation Trust<br>Lamb, Sarah; University of Exeter, College of Medicine and Health; University of Oxford, NDORMS
Keywords:	Rheumatology < INTERNAL MEDICINE, Adult orthopaedics < ORTHOPAEDIC & TRAUMA SURGERY, Hip < ORTHOPAEDIC & TRAUMA SURGERY, Knee < ORTHOPAEDIC & TRAUMA SURGERY

SCHOLARONE™
Manuscripts

I, the Submitting Author has the right to grant and does grant on behalf of all authors of the Work (as defined in the below author licence), an exclusive licence and/or a non-exclusive licence for contributions from authors who are: i) UK Crown employees; ii) where BMJ has agreed a CC-BY licence shall apply, and/or iii) in accordance with the terms applicable for US Federal Government officers or employees acting as part of their official duties; on a worldwide, perpetual, irrevocable, royalty-free basis to BMJ Publishing Group Ltd ("BMJ") its licensees and where the relevant Journal is co-owned by BMJ to the co-owners of the Journal, to publish the Work in this journal and any other BMJ products and to exploit all rights, as set out in our licence.

The Submitting Author accepts and understands that any supply made under these terms is made by BMJ to the Submitting Author unless you are acting as an employee on behalf of your employer or a postgraduate student of an affiliated institution which is paying any applicable article publishing charge ("APC") for Open Access articles. Where the Submitting Author wishes to make the Work available on an Open Access basis (and intends to pay the relevant APC), the terms of reuse of such Open Access shall be governed by a Creative Commons licence – details of these licences and which Creative Commons licence will apply to this Work are set out in our licence referred to above.

Other than as permitted in any relevant BMJ Author's Self Archiving Policies, I confirm this Work has not been accepted for publication elsewhere, is not being considered for publication elsewhere and does not duplicate material already published. I confirm all authors consent to publication of this Work and authorise the granting of this licence.

For peer review only

BMJ OPEN

TITLE PAGE

Title: Behaviour change physiotherapy intervention to increase physical activity following hip and knee replacement: the PEP-TALK randomised controlled trial

Authors: Smith TO,[1,2] Parsons SR,[1] Ooms AG,[1,3] Dutton SJ,[1,3] Fordham B,[1] Garrett A,[1] Hing CB,[4] Lamb SE[1,5] on behalf of the PEP-TALK Trial Collaborators.

Affiliations

1. Nuffield Department of Rheumatology, Orthopaedics and Musculoskeletal Sciences, University of Oxford, Botnar Research Centre, Windmill Road, Oxford, UK
2. Faculty of Medicine and Health Sciences, University of East Anglia, Norwich, UK
3. Oxford Clinical Trials Research Unit (OCTRU), Centre for Statistics in Medicine (CSM), University of Oxford, Windmill Road, Oxford, UK
4. Department of Trauma and Orthopaedics, St George's Hospital, St George's University Hospitals NHS Foundation Trust, Tooting, London, UK
5. College of Medicine and Health Sciences, University of Exeter, Exeter, UK

Corresponding Author: Dr Toby Smith, School of Health Sciences, Queen's Building, University of East Anglia, Norwich, UK. Email: toby.smith@uea.ac.uk

Word Count (currently): Abstract: 299 (limit 300); Main Manuscript: 4330

For peer review only

ABSTRACT

OBJECTIVE: To test the effectiveness of a behaviour change physiotherapy intervention to increase physical activity compared with usual rehabilitation after Total Hip Replacement (THR) or Total Knee Replacement (TKR).

DESIGN: Multicentre, pragmatic, two-arm, open randomised controlled superiority trial

SETTING National Health Service providers in nine English hospitals.

PARTICIPANTS: 224 individuals aged ≥18 years, undergoing a primary THR or TKR deemed "moderately inactive" or "inactive".

INTERVENTION: Participants received either six, 30-minute, weekly, group-based exercise sessions (usual care), or the same six-weekly, group-based, exercise sessions each preceded by a 30-minute cognitive behaviour discussion group aimed at challenging barriers to physical inactivity following surgery (experimental).

RANDOMISATION & BLINDING: Initial 75 particiapants were randomised 1:1 before changing the allocation ratio to 2:1 (experimental:usual care). Allocation was based on minimisation, stratifying on comorbidities, operation type and hospital. There was no blinding.

MAIN OUTCOME MEASURES: Primary: UCLA Activity Score at 12 months. Secondary: six and 12 month assessed function, pain, self-efficacy, kinesiophobia, psychological distress and quality of life.

RESULTS: Of the 1254 participants assessed for eligibility, 224 were included (139 experimental:85 usual care). Mean age was 68.4 years (standard deviation: 8.7), 63% were female, 52% underwent TKR. There was no between-group difference in UCLA score (mean difference: -0.03 (95% CI: -0.52 to 0.45, p=0.89)). There were no differences observed in any of the secondary outcomes at six or 12 months. There were no important adverse events in either group. The COVID-19 pandemic contributed to the reduced intended sample size (target 260) and reduced intervention compliance.

CONCLUSIONS: There is no evidence to suggest attending usual care physiotherapy sessions plus a group-based behaviour change intervention differs to attending usual care physiotherapy alone. As the trial could not reach its intended sample size, nor a proportion of participants receive their intended rehabilitation, this should be interpreted with caution.

TRIAL REGISTRATION: ISRCTN29770908

Keywords: arthroplasty; osteoarthritis; rehabilitation; physical activity; exercise; cognitive behavioural

STRENGTHS AND LIMITATIONS OF THIS STUDY

- The multicentre recruitment approach enhanced external validity across population characteristics in England.

For peer review only

- Functional, behavioural and psychological outcomes were collected to ensure a global participant assessment.
- It was challenging to ensure there were acceptable numbers of people in the group-based intervention.
- All 12-month follow-up data were collected during the COVID-19 pandemic potentially impacting on typical recovery and psychological outcomes.
- The COVID-19 pandemic meant we were unable to reach our anticipated sample size or deliver the intervention as planned.

INTRODUCTION

Total Hip Replacement (THR) and Total Knee Replacement (TKR) are two highly successful orthopaedic procedures which reduce pain for people with osteoarthritis.[1,2] Over 200,000 THRs and TKRs were performed in the United Kingdom (UK) in 2019 pre-pandemic.[1] Approximately 90% of patients are typically satisfied following THR and TKR,[2] with significant improvements in pain and physical function after three to 12 months.[2,3]

Historically, it has been assumed that people become more active following THR or TKR through the amelioration of joint pain.[4] However, current literature suggests physical activity, at best, remains the same from pre- to post-operatively, and in some instances declines.[4,5]

People following THR and TKR have reported a number of challenges which make engaging in physical activity difficult, most notably psychosocial barriers and fear avoidance beliefs.[6] Such barriers include receiving insufficient and inconsistent information on being more physically active, fear of damaging joint replacements and causing pain, and not being able to goal-set or problem-solve physical activities within individual's lifestyles.[6] Whilst previous international guidance has knowledged the importance of physical activity on health and wellbeing, people following THR and TKR have reported difficulty in being active.[6] There is limited support or guidance currently offered on how to overcome these problems post-operatively.[6]

Not being physically active after joint replacement can have a major negative impact on a person's health and a burden on the National Health Service (NHS). Medical co-morbidities are common in this population. These include hypertension (56%),[7] cardiovascular disease (20%),[8] diabetes (16%)[8] and multi-joint pain (57%).[7] Approximately 27% of people who undergo joint replacement have three or four comorbidities.[8] Medical comorbidities have a significant negative impact on both health-related quality of life (HRQoL) and result in a societal burden.[9,10] Participating in regular physical activity can decrease the risk of cardiovascular disease by 52%,[11] diabetes by 65%,[12] and some cancers by 40%.[13] It is associated with a reduction in all-cause mortality by 33% and cardiovascular mortality by 35%.[14]

Current rehabilitation following THR and TKR in the UK, as advocated by the National Institute for Health and Care Excellence (NICE), centres around regaining joint movement, strength and gait re-education.[15] There is currently no evidence informing patients or healthcare professionals on how to increase physical activity specifically following THR and TKR. Following joint replacement, people have specific psychological needs and challenges which differ to the non-joint replacement population.[6] Therefore, a specific intervention tailored to this population's health beliefs, including

For peer review only

fear avoidance regarding implant survival, dislocation and increased knowledge on the impact of physical inactivity on other comorbidities, is required. Previous research has demonstrated that behaviour-change interventions can effectively increase physical activity across the lifespan.[16-20] Given this, it was hypothesised that such an intervention could be beneficial for this population. Accordingly, the purpose of this trial was to answer the research question "following a primary THR or TKR, does a group exercise and behaviour-change intervention targeted to increase physical activity participation increase HRQoL and clinical outcomes over the initial 12 post-operative month compared to group exercise alone?"

METHODS

Study design

A full protocol has been published previously.[21]

This was a two-arm, open, pragmatic, parallel, multi-centre, randomised controlled superiority trial. The study flow chart is presented as **Figure 1**. Participants were recruited from eight UK NHS hospital trusts by the clinical team once they had been listed for THR or TKR. Interventions were delivered in physiotherapy departments within these NHS facilities.

We recruited adults who were due to undergo primary unilateral THR or TKR where the indication for surgery was degenerative joint pathology (not trauma). Potential participants were classified as 'moderately inactive' or 'inactive' using the General Practice Physical Activity Questionnaire (GPPAQ)[22] and have a Charlson Comorbidity Index (CCI) of ≥1 point.[23,24] We excluded people who were cognitively impaired, defined as an Abbreviated Mental Test Score (AMTS)[25] of <8; whose usual place of residence was a care home; were unable to read and/or comprehend English; and had no access to a working telephone.

Study treatments

Usual NHS surgical and in-patient care was received by both control and intervention groups. On hospital discharge, all participants attended six-weekly, 30-minute, group-based exercise classes within each hospital trust's physiotherapy department. These groups commenced within four weeks post-operation. The principles regarding prescription of group exercises to increase range of motion, strength and gait pattern were consistent. Whilst the rehabilitation of THR and TKR focuses on overall lower limb function, all participants following a THR focused on hip exercises, whereas those following a TKR focused on knee exercises. One physiotherapist (with or without a second physiotherapist or therapy assistant) ran each session.

The programme and rationale for the experimental intervention are presented in detail in **Supplementary File 1**. In brief, participants randomised to the experimental group received the same six-weekly, group-based, 30-minute, exercise session as the usual care group. The only difference between the two groups was the addition of a 30-minute, group-based, behaviour change intervention prior to the routine 30 minutes of exercise, and three telephone-follow-up calls two, four and six weeks after the last group-based session. In the group-based sessions, participants were facilitated (as a group) to develop skills to overcome challenges to physical activity behaviour, supplemented through a workbook. This encouraged reflective activities such as recording physical, emotional and cognitive barriers and facilitators to physical activity. One physiotherapist (with or

For peer review only - http://bmjopen.bmj.com/site/about/guidelines.xhtml

without a second physiotherapist or therapy assistant) ran each session. During the follow-up telephone calls, participant's goals were reviewed, any barriers to the completion of these goals were identified, and the physiotherapist reviewed any 'unhelpful' and 'helpful' thoughts or feelings towards physical activity which may have arisen since the last consultation, and closed with the development of longer-term physical activity goal-setting. A treatment log was completed by the physiotherapists to record the components of what was discussed across the group in each session and each telephone call.

Each member delivering the experimental intervention attended a one-day training session which taught the components and format of the intervention. To ensure compliance with the treatment protocol, we made regular visits for quality assurance.

Data collection

At the time of enrolment, site health professionals checked eligibility and recorded demographic characteristics. We obtained baseline scores for outcome questionnaires before randomisation. Data collected at baseline included: gender, age, height and weight, CCI, self-reported presence and location of multi-site joint pain, co-morbidities determined from the medical notes, AMTS, employment status and occupation (when appropriate).

Participants were followed-up at six and 12 months after randomisation.

The primary outcome was the University of California Los Angeles (UCLA) Activity Score[26] (scored 0 to 10; higher scores indicate greater physical activity) at 12 months. Secondary outcomes at six and 12 months after randomisation were measured using the Lower Extremity Functional Scale (LEFS)[27] (scored 0 to 80, higher scores indicating less functional disability), Oxford Hip Score (OHS)[28] or Oxford Knee Score (OKS)[29] (scored 0 to 48, higher scores indicating less disease-specific function), Numerical Rating Scale (NRS) for pain (scored 0 to 10, higher scores indicating greater pain perception), the Generalized Self-Efficacy Scale (GSES)[30] (scored 10 to 40, higher scores indicating greater self-efficacy), the Tampa Scale for Kinesiophobia[31] (scored 17 to 68, higher scores indicating greater fear of motion), the Hospital Anxiety and Depression Scale (HADS)[32] (scored 0 to 21, higher scores indicating greater anxiety and depression), and the EQ-5D-5L[33] (scored 0 to 1, higher scores indicating greater HRQoL). Participants provided a retrospective assessment of complications at each six-month follow-up period. Health resource ultilisation data was collected but is not presented in this paper.

For each participant in the experimental intervention arm, the number of trial exercise sessions attended and group size of each session was recorded. The number of telephone contacts made after the end of the sessions and adherence with intervention protocols was also collected. There were no changes to the outcomes during the trial.

Randomisation and masking

Random allocation was 1:1 originally. Randomisation was performed using a centralised computer randomisation program provided by the Oxford Clinical Trials Research Unit (OCTRU). Research nurses and physiotherapists at recruiting centres enrolled participants and assigned participants by accessing the online OCTRU randomisation program. Randomisation was undertaken using a minimisation algorithm, stratified by: hospital site; type of joint replacement (THR or TKR); CCI of one to three versus ≥4.[23,24] It had a probabilistic element introduced to ensure unpredictability of treatment assignment. To facilitate larger class sizes, we modified the randomisation ratio to 2:1 in favour of the experimental intervention after 75 randomisations. Full rationale for the change can be found in the published protocol.[21]

For peer review only

Masking participants or the teams providing interventions was not possible.

Sample size

Originally, 250 participants (125 per arm) were required to detect a standardised effect size of 0.4 with 80% power and 5% (two-sided) significance, and allowing for 20% loss to follow-up. These calculations were based on the primary outcome, UCLA Activity Score at 12 months, assuming a baseline standard deviation of 2.5 and a between-group difference of one.[34] The minimally clinically important difference (MCID) was reported as a within-person difference of 0.92 points.[34]

The target sample size was increased to 260 to account for the change in randomisation ratio.[21]

Statistical methods

There was no planned interim analyses or pre-defined stopping rules. Full analysis details are in the published statistical analysis plan.[35]

Main analyses were performed on the intention-to-treat population. If a participant had observed data on any of the time points, they were included. Linear mixed effects models were used for the primary and continuous secondary outcomes. These models adjusted for person within centre as random effects, and CCI score and corresponding baseline measure (as continuous outcomes), type of operation, time (six or 12 months) and treatment as fixed effects. A treatment by time interaction was included to allow time specific treatment effect estimates to be calculated. Adjusted mean differences with corresponding 95% confidence intervals (CI) and p-values were presented. The number of participants with at least one complication was analysed using logistic regression adjusting for minimisation factors and treatment. Total number of complications has been analysed using a Poisson regression. The same factors as used in the logistic regression are used in the Poisson regression.

Supporting analyses to the primary outcome included an area under the curve (AUC) analysis and complier average causal effect (CACE) analyses for all three pre-defined levels of compliance (Strict Compliance, Compliance, Attendance).[35] Full definitions of the three compliance levels are given in **Supplementary File 2.** The AUC analysis was performed using the same model as used for the primary analysis except including baseline UCLA Activity Score in the "time" fixed effect allowing time point specific treatment effects to be calculated for baseline, six and 12 months. The CACE analysis has been performed through 10000 bootstrapped samples. Adjusted linear regression was used for the 12-month UCLA Activity Score; adjusting for randomised treatment, baseline UCLA Activity Score, recruiting site, CCI (continuous), and joint replacement was used to obtain ITT estimates. The pathway from treatment allocation to compliance (rate of potential compliers in the usual care group) was also estimated using adjusted linear regression: compliance indicators was analysed adjusting for the same variables. CACE estimates were obtained by taking the ratio of the ITT estimate and potential complier rate. Standard errors, confidence intervals and p-values were calculated using the bootstrapped samples.

Other analyses examining the missing data assumptions, the per-protocol population, using a reduced model, treatment effects within pre-defined clinical subgroups and exploratory descriptive statistics for selected secondary outcomes by COVID-19.

For peer review only

Study monitoring

A Trial Steering Committee (TSC) and Data Safety Monitoring Committee (DSMC) were appointed to independently review data on safety, protocol adherence and trial recruitment.

Patient and public involvement

Patient involvement began during protocol development and continued throughout the trial. A patient-member attended TSC meetings. The same patient-member was a co-investigator. He provided insights into the trial conduct, particularly on data collection processes and helped interpret the findings to inform the trial's dissemination phase. Participants who expressed an interest in receiving information on the trial findings were provided with this.

RESULTS

Recruitment and participant flow

Recruitment occurred between 12 April 2019 to 27 March 2020. The CONSORT[36] flow chart is presented as **Figure 1.** In total, 230 participants were randomised. Six were randomised in error, resulting in an analysable population of 224 participants (85 usual care; 139 experimental).

Due to the COVID-19 pandemic, 47 participants that had consented to take part in the study could not be randomised and the trial was stopped 30 participants short of its planned sample size. All elective THRs and TKRs were cancelled as part of the UK national COVID-19 lockdown (23rd March 2020). Group-based physiotherapy classes within the participating hospital outpatient settings (a mechanism this trial relied on for both treatment groups) were also halted. Consequently, it was not feasible to continue the trial for the final 30 planned participants.

Retention

The retention of participants is presented in **Figure 1**. There were 37 withdrawals (13 usual care; 24 experimental). **Supplementary Table 1** gives a summary of type of withdrawals by level of withdrawal and treatment group. The return of primary outcome data is presented in **Supplementary Table 2**. This illustrates that for the primary, ITT, analysis of the UCLA Activity Score there were 223 (99.6%) participants to supply a UCLA Activity Score at baseline (85 usual care; 138 experimental), 186 (83.0%) responses at six months (69 usual care; 117 experimental) and 181 (80.8%) responses at 12 months (70 usual care; 111 experimental).

Participant characteristics

Baseline characteristics are presented by randomised treatment group in **Table 1**. The mean participant age was 68.4 years (standard deviation (SD): 8.7), 62.9% were female with 52.2% undergoing TKR. Seventy-four percent of the cohort had a CCI of one to three (mean 2.9 (SD: 1.3)). Mean BMI was 30.9kg/m^2 (SD: 5.7). The mean duration of symptoms prior to surgery was 46.9 months (SD: 50.9) with 73.2% presenting with an American Society of Anesthesiology (ASA) grade of two at surgery. As **Table 1** demonstrates, the two groups were comparable with the experimental group presenting with a slightly higher proportion of females (64.7% vs. 60.0%), longer duration of symptoms

(mean: 48.8 months vs. 43.8 months) and fewer inactive participants (79.1% vs. 83.5%) compared to the usual care group.

Table 1: Baseline characteristics by randomised group

	**Usual (n=85)**	**Experimental (n=139)**	**Total (n=224)**
Age, years	n=85, 68.5 (8.8)	n=139, 68.3 (8.6)	n=224, 68.4 (8.7)
UCLA Activity Score, 1-10	n=85, 3.6 (1.5)	n=138, 3.6 (1.6)	n=223, 3.6 (1.5)
**Joint Replacement**			
Hip replacement	40 (47.1)	67 (48.2)	107 (47.8)
Knee replacement	45 (52.9)	72 (51.8)	117 (52.2)
**CCI, Dichotomised**			
1-3	64 (75.3)	102 (73.4)	166 (74.1)
4+	21 (24.7)	37 (26.6)	58 (25.9)
CCI, Continuous	n=85, 2.8 (1.3)	n=139, 3.0 (1.3)	n=224, 2.9 (1.3)
**Sex**			
Female	51 (60.0)	90 (64.7)	141 (62.9)
Male	34 (40.0)	49 (35.3)	83 (37.1)
**BMI, Categories**			
Healthy Weight	15 (17.6)	25 (18.0)	40 (17.9)
Overweight	22 (25.9)	45 (32.4)	67 (29.9)
Obese	42 (49.4)	60 (43.2)	102 (45.5)
Morbidly Obese	6 (7.1)	9 (6.5)	15 (6.7)
BMI, kg/m2	n=85, 31.1 (5.9)	n=139, 30.7 (5.6)	n=224, 30.9 (5.7)
**Joint Pain in the Past 7 Days**			
Yes	85 (100.0)	138 (99.3)	223 (99.6)
No	0 (0.0)	1 (0.7)	1 (0.4)
**GPPAQ Level**			
Active	0 (0.0)	0 (0.0)	0 (0.0)
Moderately Active	2 (2.4)	1 (0.7)	3 (1.3)
Moderately Inactive	12 (14.1)	28 (20.1)	40 (17.9)
Inactive	71 (83.5)	110 (79.1)	181 (80.8)
AMTS	n=85, 9.6 (0.6)	n=139, 9.6 (0.6)	n=224, 9.6 (0.6)
EQ-5D-5L Score	n=85, 0.4 (0.2)	n=139, 0.4 (0.3)	n=224, 0.4 (0.2)
EQ-VAS, 0-100	n=85, 61.3 (20.0)	n=139, 60.6 (23.6)	n=224, 60.9 (22.2)
Numeric Pain, 0-10	n=85, 6.9 (1.9)	n=139, 7.2 (1.8)	n=224, 7.1 (1.9)
Symptom Duration, Months	n=85, 43.8 (48.8)	n=138, 48.8 (52.2)	n=223, 46.9 (50.9)
**ASA Classification**			
1	4 (4.7)	12 (8.6)	16 (7.1)
2	61 (71.8)	103 (74.1)	164 (73.2)
3	20 (23.5)	22 (15.8)	42 (18.8)
4	0 (0.0)	2 (1.4)	2 (0.9)

Data are mean (SD-standard deviation) or n (%). +Stratification factor used in randomisation. UCLA=University of California, Los Angeles, CCI=Charlson Comorbidity Index, BMI=Body Mass Index, GPPAQ=General Practice Physical Activity Questionnaire, AMTS=Abbreviated Mental Test Score, EQ-5D-5L=Health-related quality of life assessed by EuroQol 5-level EQ-5D, EQ-VAS=EuroQol Visual Analogue Scale.

Main analyses

The results of the analysis for the primary outcome measure are presented in **Table 2** and **Figure 2**. There was no evidence to support rejecting the null hypothesis that there was no difference between attending group-based exercise plus a group-based behaviour change intervention and attending group-based exercise alone on the UCLA Activity Score at 12 months post-randomisation, at the 5% significance level (mean difference: -0.03; 95% CI: -0.52 to 0.45; p=0.89). However, as the trial could not reach its intended final sample size due to the COVID-19 pandemic, this result should be interpreted with caution. The interpretation of the results did not change on per-protocol analysis or reduced model analysis (**Supplementary Table 3; Supplementary Table 4**).

Table 2: UCLA Activity Score (primary outcome) results

Time Point	Usual	Experimental	Mean Difference		p-value
	n, Mean (SD)	n, Mean (SD)	Unadjusted	Adjusted (95% CI)	
Baseline	n=85, 3.62 (1.52)	n=138, 3.57 (1.57)	-0.06	-	-
6 Months	n=69, 4.77 (1.52)	n=117, 4.97 (1.68)	0.20	0.27 (-0.21,0.76)	0.27
**12 Months (Primary Outcome)**	**n=70, 4.87 (1.61)**	**n=111, 4.84 (1.91)**	**-0.03**	**-0.03 (-0.52,0.45)**	**0.89**
Area under the curve over 12 months	4.81 (0.29)	4.89 (0.28)	-	0.09 (-0.47,0.64)	0.88
CACE: Strict Compliance	-	n=46	-	-0.24 (-1.45,0.96)	0.69
CACE: Compliance	-	n=58	-	-0.20 (-1.19,0.79)	0.69
CACE: Attendance	-	n=81	-	-0.16 (-0.90,0.59)	0.68

N - number of participants; SD – standard deviation; CACE – complier average causal effect.
For the AUC analysis, the standard deviations presented are the standard errors for these estimates calculated using the delta method. CACE analysis based on 10000 bootstrapped samples.

Three Complier Average Causal Effect (CACE) estimation were performed on the 12-month UCLA Activity Score, one for each definition of compliance (Strict Compliance, Compliance and Attendance). **Table 2** presents the CACE estimates for the three levels of compliance. There was no difference in outcome based on these analyses and all effect estimates were within the MCID of 0.92.[34]

The results of all continuous secondary outcomes are presented in **Table 3**. They demonstrate no significant between-group differences for any of the continuous secondary outcomes at any time point. A general pattern of improvement from baseline to six months then levelling off at 12 months with no significant between-group differences observable was seen throughout.

A total of 141 complications were reported from 75 participants, 50 (35.5%) in the usual care group and 91 (64.5%) in the experimental group (**Table 4**; **Supplementary Figure 1**). It is should be noted that 62.1% of participants were randomised to the experimental group so this apparent difference is expected if complication rate was the same across both groups The most common complications were increased pain either in the operated joint or in other joints, wound infections, medical complications and stiffness in the operated joint. Most complications (65.2%) were reported in the first six months post-randomisation. There was no difference in the number of people who had a complication (28 vs. 47; odd ratio (OR): 1.03; 95% CI: 0.56 to 1.89) or total numbers of complications (50 vs. 91; OR: 1.10; 95% CI: 0.77 to 1.56) between the usual care and experimental group respectively. There was one adverse event (fall, usual care) and three serious adverse events (two experimental (cardiac failure, pneumonia), one usual care (suspected deep vein thrombosis)).

For peer review only - http://bmjopen.bmj.com/site/about/guidelines.xhtml

Table 3: Continuous secondary outcome results

Time Point	Usual	Experimental	Mean Difference		p-value
	n, Mean (SD)	n, Mean (SD)	Unadjusted	Adjusted (95% CI)	
**Lower Extremity Functional Scale**					
Baseline	n=82, 23.72 (13.11)	n=130, 24.50 (14.07)	0.78	-	-
6 Months	n=45, 45.40 (19.76)	n=80, 51.44 (17.70)	6.04	2.60 (-3.29,8.50)	0.39
12 Months	n=51, 47.86 (18.97)	n=80, 50.67 (21.40)	2.81	1.26 (-4.61,7.13)	0.67
**Oxford Hip Score**					
Baseline	n=40, 16.05 (6.36)	n=67, 16.78 (7.99)	0.73	-	-
6 Months	n=28, 34.84 (11.73)	n=50, 39.68 (8.93)	4.84	3.86 (-0.92,8.64)	0.11
12 Months	n=27, 36.90 (12.48)	n=48, 39.42 (10.46)	2.52	2.37 (-2.53,7.27)	0.34
**Oxford Knee Score**					
Baseline	n=45, 18.67 (8.51)	n=72, 17.46 (6.99)	-1.21	-	-
6 Months	n=33, 35.20 (7.62)	n=51, 33.45 (9.38)	-1.75	-1.74 (-5.03,1.54)	0.30
12 Months	n=35, 34.90 (8.46)	n=55, 33.54 (9.84)	-1.36	-1.43 (-4.72,1.86)	0.39
**Numerical Rating Scale for Pain**					
Baseline	n=85, 6.87 (1.94)	n=139, 7.23 (1.79)	0.36	-	-
6 Months	n=61, 3.34 (2.59)	n=101, 3.54 (2.74)	0.20	0.19 (-0.64,1.02)	0.66
12 Months	n=61, 4.08 (2.87)	n=102, 3.33 (2.85)	-0.75	-0.75 (-1.59,0.09)	0.08
**Generalized Self-Efficacy Scale**					
Baseline	n=84, 31.31 (5.49)	n=138, 31.67 (5.39)	0.36	-	-
6 Months	n=58, 31.88 (5.18)	n=98, 33.03 (5.30)	1.15	1.15 (-0.30,2.61)	0.12
12 Months	n=61, 32.16 (5.55)	n=101, 32.20 (6.72)	0.03	0.33 (-1.13,1.78)	0.66
**Tampa Scale for Kinesiophobia**					
Baseline	n=85, 40.04 (7.44)	n=136, 39.77 (7.75)	-0.26	-	-
6 Months	n=56, 35.77 (7.74)	n=91, 34.77 (7.29)	-1.00	-0.39 (-2.40,1.61)	0.70
12 Months	n=57, 36.56 (6.91)	n=90, 35.06 (8.27)	-1.51	-0.77 (-2.79,1.24)	0.45
**Hospital Anxiety and Depression Scale (Overall)**					
Baseline	n=85, 11.85 (6.16)	n=138, 12.50 (7.07)	0.65	-	-
6 Months	n=59, 8.97 (6.52)	n=97, 8.81 (6.36)	-0.15	-1.18 (-2.73,0.37)	0.14
12 Months	n=62, 9.02 (6.61)	n=98, 9.70 (6.99)	0.69	0.52 (-1.03,2.06)	0.51
**Hospital Anxiety and Depression Scale (Anxiety)**					
Baseline	n=85, 5.89 (3.78)	n=138, 6.63 (4.07)	0.74	-	-
6 Months	n=60, 4.95 (4.01)	n=98, 4.95 (3.57)	0.00	-0.71 (-1.67,0.25)	0.15
12 Months	n=62, 4.76 (3.73)	n=99, 5.46 (3.84)	0.71	0.36 (-0.60,1.31)	0.46
**Hospital Anxiety and Depression Scale (Depression)**					
Baseline	n=85, 5.95 (3.16)	n=139, 5.89 (3.81)	-0.06	-	-
6 Months	n=61, 4.03 (3.27)	n=99, 3.90 (3.51)	-0.13	-0.25 (-1.13,0.63)	0.58
12 Months	n=62, 4.26 (3.47)	n=101, 4.30 (4.02)	0.04	0.24 (-0.65,1.12)	0.60
**EQ-5D-5L Index**					
Baseline	n=85, 0.40 (0.22)	n=139, 0.39 (0.27)	-0.01	-	-
6 Months	n=68, 0.66 (0.23)	n=117, 0.69 (0.25)	0.03	0.03 (-0.03,0.10)	0.31
12 Months	n=70, 0.67 (0.24)	n=113, 0.67 (0.29)	0.00	0.00 (-0.06,0.07)	0.93
**EQ-VAS**					
Baseline	n=85, 61.33 (20.01)	n=139, 60.58 (23.56)	-0.75	-	-
6 Months	n=68, 70.93 (18.67)	n=117, 73.86 (20.02)	2.94	2.84 (-2.31,7.99)	0.28
12 Months	n=69, 72.51 (17.90)	n=110, 72.94 (19.98)	0.43	1.47 (-3.73,6.68)	0.58

Table 4: Complication results

	Usual N (%)	Experimental N (%)	Odds Ratio (95% CI)	p-value
Number of participants who had a complication	28 (32.94)	47 (33.81)	1.03 (0.56,1.89)	0.94
Total complications	50 (58.82)	91 (65.47)	1.10 (0.77,1.56)	0.61

CI – confidence intervals

Analysis by compliance

Treatment compliance is summarised in **Supplementary Figure 2**. Compliance is reported by categories as defined in the analysis plan.[35] In total, 489 experimental intervention or physiotherapy exercises sessions were held. The sessions ran from 08 May 2019 to 18 March 2020. 162 were experimental sessions and 327 were exercise alone sessions (161 usucal care; 166 experimental). There was one experimental class that was not accompanied by a physiotherapy class.

A major component of the definition of compliance for the experimental group was the group class sizes. The median class size for the intervention classes was two with a range of one to 14. **Supplementary Figure 3** is a plot of the group sizes for all intervention sessions. Any class with three or more participants was considered a “compliant” class. In total, 75 (46.3%) of the 162 intervention sessions had three or more participants. To address the issue of compliance, the randomisation procedure was changed from 1:1 to 2:1. **Supplementary Figure 4** is a breakdown of treatment compliance by participants randomised using either a 1:1 or 2:1 randomisation ratio. In both groups, the number of participants who were non-compliant rose considerably and the number of strict compliers fell after the change from 1:1 to 2:1 randomisation. A confounder to this result is that participants whose intervention was disrupted by COVID-19 were all randomised using a 2:1 ratio. The large increase in non-compliance in that population is seen in **Supplementary Figure 4**.

Impact of COVID-19 on trial findings

The level of disruption to the intervention delivery caused by the COVID-19 pandemic was high. There was a high level of non-compliance, particularly in the experimental group. This apparent between-group difference in non-compliance was because the pre-defined definitions of compliance were stricter in the experimental than the usual care group. To be an “Attender” in the experimental group, one needed to attend four out of six group intervention sessions, to achieve the same level of compliance in the usual care group, only one session was required to be attended. In the usual care group, 66 (77.6%) attended at least one physiotherapy session, a similar proportion, 111 (80%), attended at least one physiotherapy session in the experimental group. Due to the added therapy the experimental group received, the definition for compliance had to be stricter but both groups had a similar proportion who attended at least one session.

The final months of the trial, before all group-based physiotherapy classes within the hospital outpatient setting were halted due to the COVID-19 pandemic, yielded the highest group sizes. **Supplementary Figure 4** summarises the compliance to the experimental group by pre-COVID-19 compared to COVID-19 to estimate the impact of the pandemic on compliance. This is plotted by time in **Supplementary File 3**. Based on this, a large proportion of participants who could not be randomised due to the trial closure would have ended up falling into either the “Compliant” or “Strict Compliant” groups.

Additional analyses

For peer review only

The missing data analysis suggests that the missing at random assumption made in the primary analysis is appropriate (**Supplementary Figure 5**), the per-protocol and reduced model results support the main findings from the trial and there was no evidence of any difference in the exploratory subgroup analysis. The exploratory descriptive statistics by COVID-19 status may suggest participants in the COVID-19 group had poorer psychological outcomes (**Supplementary Table 5**). The results are presented in full in **Supplemental Figure 6**.

DISCUSSION

The findings suggest that following THR or TKR, there is no difference between the addition of a group-based exercise and behaviour change intervention in physical activity and other clinical outcomes during the first post-operative year compared to attending group-based exercise alone. However, the COVID-19 pandemic significantly impacted on this trial whereby the intended sample size was not achieved, and a considerable proportion of participants were unable to receive their intended post-operative rehabilitation. Accordingly, these findings should be interpreted with caution.

The rationale for undertaking this study was the uncertainty over how to increase physical activity following THR and TKR. Whilst several studies have been published over the intervening period acknowledging that physical activity remains low following joint replacement,[37-39] there continues to be uncertainty over how to overcome this. Studies in other populations, most notably older adults, individuals with chronic respiratory disorders and those with chronic rheumatological diseases have provided promise that a behaviour change intervention may improve physical activity.[17-20] However, as previously acknowledged, the specific challenges which individuals face in relation to fear avoidance, beliefs about implant failure, multi-joint pain and other comorbidities[6] may account for why this behaviour change intervention did not demonstrate similar changes. However, the results from this trial have been impacted by the COVID-19 pandemic, principally on intervention delivery and compliance. Given the impact COVID-19 had, there still remains a need to better understand how to increase physical activity following THR or TKR.

Trial participants understood the research objective was to explore the effectiveness of an intervention aimed at increasing physical activity following THR or TKR. However, compliance to the intervention was low throughout the trial. Accordingly, the appetite to increase physical activity remains uncertain. Previous literature has suggested that whilst individuals may be no more physically active after joint replacement,[39,40] clinical outcomes and specifically pain do significantly improve.[41,42] This corresponds with an improvement in HRQoL. Patient satisfaction to outcome and expectations may be met but this is not translated into increased physical activity. Given the wider health benefits which physical activity confers, consideration should be made on how health professionals promote physical activity messages within post-operative recovery programmes so added health gains are maxmised. How this is operationalised following this trial's findings, remains unclear.

Whilst the results indicate no superiority to the addition of a behaviour change intervention to usual physiotherapy rehabilitation after TKR or THR, the findings offer important clinical implications. Firstly, the trial indicates that joint replacement and usual physiotherapy rehabilitation can improve clinical outcomes. Previous literature suggests improvements in pain, function and HRQoL[41,42] for people following THR and TKR. However, the trial also indicates both pre- and post-COVID-19 that there were differences in adherence and compliance to both usual and experimental physiotherapy interventions. Whilst previous literature has highlighted geographical and service-provision differences in

For peer review only

rehabilitation after joint replacement,[43,44] there has been limited evidence to indicate variability in adherence to rehabilitation. This may reflect variation in rehabilitation need. Whilst some patients may need substantial levels of physiotherapy following joint replacement to promote physical function, activity and improvements in HRQoL, these may not be homogeneous within the population.[45] Stratification on rehabilitation need may therefore be warranted. Whilst previous authors have attempted to identify those at most risk of poor outcomes post-operative,[46,47] there remains uncertainty over what physiotherapy intervention is more beneficial for these patients. Further consideration on the optimal rehabilitation programme to promote physical activity for those with the most to gain as opposed to assuming all, as adopted in this trial, may be indicated.

There are several trial strengths and limitations to be considered. A major strength was the pragmatic approach taken to assess effectiveness. The broad eligibility criteria to reflect typical patients who undergo THR and TKR, balanced by the inclusion of only those, who were pre-operatively moderately inactive or inactive, meant the eligibility criteria were constructed to theoretically recruit those who had the most to gain. The multi-site, national recruitment process across NHS health trusts also offered the ability to recruit a diverse cohort in relation to socioeconomic, ethnic and geographical factors. However, a limitation to the design was that several measures which may have characterised such diversity including level of deprivation, educational status, ethnicity and educational background were not collected. This decision was made to offer a more efficient data collection process, not over-burdening participants with extensive demographic data requests. Smith et al[48] previously acknowledged this as a recurrent limitation to musculoskeletal research. Future research should consider the impact of socioeconomic and deprivation factors both on the design of interventions, processes and analysis. A further limitation was the impact of COVID-19. Whilst acknowledged that the trial over-recruited, consenting 277 participants, only 230 were randomised as the pandemic disrupted surgical and rehabilitation delivery. This means the results were underpowered to answer the trial's primary research question. Secondly, 69 individuals who were receiving rehabilitation during this time had their intervention delivery impacted on this change in service provision. Consequently, intervention compliance reduced, impacting on any effect estimate generated from that point onwards. Given this equated to 30% of the cohort, it is proposed this had a significant impact. What is more difficult to estimate is the impact of the COVID-19 social restrictions on outcome. All participants experienced the 2020 social restrictions prior to completing their 12-month questionnaires (first 12-month questionnaire completed 23 March 2020). Whilst previous studies[49,50] indicate that individuals with joint pain substantially reduced their natural physical activity engagement during this time, we did not specifically collect data to ascertain the effects of 'lockdown' on outcomes. The effect of this on 12-month results should therefore be considered.

For peer review only

CONCLUSIONS

The addition of a group-based behaviour change intervention to usual physiotherapy rehabilitation following primary THR and TKR does not offer benefit over usual physiotherapy alone on physical activity and clinical outcomes over the first 12 post-operative months. These findings should be viewed with caution as the COVID-19 pandemic impacted on both the ability of participants to undergo joint replacement and compliance to their rehabilitation. Given the health and social benefits which being active offers older adults, further exploration on methods to increase physical activity for those who are inactive following joint replacement remains important.

DECLARATIONS AND ACKNOWLEDGEMENTS

Authors' Contributors: TS, BF, SJD, SL, AO, CH, AG, SP researched the topic and devised the study. TS, SP, AO, BF, SJD, CH, AG, SL provided the first draft of the manuscript. AO, SD provided statistical analysis and oversight. TS, SP, BF, AO, SJD, CH, AG, SL contributed equally to manuscript preparation. TS acts a guarantor.

Funding Statement: The research is supported by the National Institute for Health Research (NIHR) Research for Patient Benefit grant (PB-PG-1216-20008). The views expressed are those of the author(s) and not necessarily those of the NHS, the NIHR or the Department of Health and Social Care.

Competing Interests Statement: None declared.

Disclaimer: The views expressed are those of the author(s) and not necessarily those of the NHS, the NIHR or the Department of Health and Social Care.

Ethics Approval: Ethical approval was gained from the South Central (Oxford B) Research Ethics Committee (Approval Date: 23Oct2018; Reference Number: 18/SC/0423).

Trial Sponsor: University of Oxford (Clinical Trials and Research Governance Team. Joint Research Office, 1st floor, Boundary Brook House, Churchill Drive, Headington, Oxford OX3 7GB. Email: ctrg@admin.ox.ac.uk. The views expressed are those of the author(s) and not necessarily those of the sponsor.

Provenance and Peer Review: Not commissioned; externally peer reviewed.

PEP-TALK Collaborators: Mr Steve Algar (Banbury, PPI Representative), Dr Zara Hansen (ZH) (University of Oxford), Dr Vicki Barber (Oxford Clinical Trials Research Unit (OCTRU), University of Oxford), Mr Malcolm Hart (OCTRU), University of Oxford), May Ee Png (OCTRU, University of Oxford), Professor Karen Barker (Principal Investigator - Oxford University Hospital NHS Foundation Trust (OUH), Mr Ian Smith (Principal Investigator - Lewisham and Greenwich University Hospitals NHS Foundation Trust), Professor Iain McNamara (Principal Investigator (Norfolk and Norwich University Hospitals NHS Foundation Trust (NNUH) & Spire Norwich), Mr Michael Dunn (Principal Investigator - St George's University Hospital NHS Foundation Trust), Mrs Dawn Lockey (Principal Investigator - City Hospitals Sunderland NHS Foundation Trust), Mr Sonny Driver (Principal Investigator – North Middlesex University Hospital NHS Trust), Mrs Jamila Kassam (Barts Health NHS Trust), Mr Peter Penny (Norwich Spire, Norwich), Mrs Celia Woodhouse, Mrs Tracey Potter and Mrs Helena Daniell (NNUH), Mr Alex Herring and Mrs Yan Cunningham (City Hospitals Sunderland NHS Foundation Trust), Irrum Afzal (South West London Elective Orthopaedic Centre), Mr Maninderpal Matharu (Barts Health NHS Trust) and Mrs Tamsin Hughes, Ms Erin Hannink and Mrs Michelle Moynihan (OUH).

Oversight Committee Membership: TSC Members: Professor David Deehan (Newcastle University), Dr Emma Godfrey (Kings College London), Dr Neil Artz (University of the West of England), Mr Steve Algar (PPI representative). **DSMC Members:** Dr Lindsey Smith (University of the West of England & Weston Area Health NHS Trust), Dr Dipesh Mistry (University of Warwick), Mr Paul Baker (South Tees Hospital NHS Foundation Trust)

Patient consent for publication: Not required.

For peer review only

Data Sharing Statement: Anonymised data will be shared outside the research team when required. Researchers outside the trial team may formally request for a specific data set using a data request form, which is part of the Data Management Plan. Data are available upon reasonable request to TS.

For peer review only

FIGURE AND TABLE LEGENDS

Figure 1: CONSORT Flow-Chart

Figure 2: UCLA Activity Score boxplots

Table 1: Baseline characteristics by randomised group

Table 2: UCLA Activity Score (primary outcome) results

Table 3: Continuous secondary outcome results

Table 4: Complication results

Supplementary File 1: PEP-TALK programme intervention outline and development

Supplementary File 2: Additional results

Supplementary Table 1: Withdrawals summary

Supplementary Table 2: Questionnaire returns by treatment group

Supplementary Table 3: UCLA Activity Score per-protocol results

Supplementary Table 4: UCLA Activity Score reduced model (no recruiting centre random effect) results

Supplementary Table 5: Descriptive results for selected secondary outcomes by COVID-19 status

Supplementary Figure 1: Complication type by randomised group

Supplementary Figure 2: Overall compliance by (a) raw frequencies and (b) percentage of randomised group

Supplementary Figure 3: Experimental intervention group sizes over time, including change from a randomisation ratio of 1:1 to 2:1

Supplementary Figure 4: Experimental intervention group compliance by COVID-19 group

Supplementary Figure 5: 12 month adjusted mean difference UCLA Activity Score for varying imputed quantiles for missing data

Supplementary Figure 6: Subgroup analyses results

For peer review only

REFERENCES

1. National Joint Registry. 18th Annual Report. Accessed 19.12.2020. Available at: NJR 18th Annual Report 2021.pdf (njrcentre.org.uk)

2. Dailiana ZH, Papakostidou I, Varitimidis S, Liaropoulos L, Zintzaras E, Karachalios T, Michelinakis E, Malizos KN. Patient-reported quality of life after primary major joint arthroplasty: a prospective comparison of hip and knee arthroplasty. *BMC Musculoskelet Disord* 2015;16:366.

3. Papakostidou I, Dailiana ZH, Papapolychroniou T, Liaropoulos L, Zintzaras E, Karachalios TS, Malizos KN. Factors affecting the quality of life after total knee arthroplasties: a prospective study. *BMC Musculoskelet Disord* 2012;13:116.

4. Withers TM, Lister S, Sackley C, Clark A, Smith TO. Is there a difference in physical activity levels in patients before and up to one year after unilateral total hip replacement? A systematic review and meta-analysis. *Clin Rehabil* 2017:316;39-50.

5. Smith TO, Hilton G, Mansfield M, Mann CJV, Sackley CM. Does physical activity change following hip and knee arthroplasty? An analysis of data from the Osteoarthritis Initiative. *Physiotherapy* 2018; 104; 80-90.

6. Smith TO, Latham S, Maskrey V, Blyth A. Patients' perceptions of physical activity before and after joint replacement: a systematic review with meta-ethnographic analysis. *Postgrad Med J 2015;91:483-91.*

7. Inacio MC, Pratt NL, Roughead EE, Graves SE. Comparing co-morbidities in total joint arthroplasty patients using the RxRisk-V, Elixhauser, and Charlson Measures: a cross-sectional evaluation. *BMC Musculoskelet Disord* 2015;16:385.

8. Peter WF, Dekker J, Tilbury C, Tordoir RL, Verdegaal SH, Onstenk R, Bénard MR, Vehmeijer SB, Fiocco M, Vermeulen HM, van der Linden-van der Zwaag HM, Nelissen RG, Vliet Vlieland TP. The association between comorbidities and pain, physical function and quality of life following hip and knee arthroplasty. *Rheumatol Int* 2015;35:1233-41.

9. Smith E, Hoy DG, Cross M, Vos T, Naghavi M, Buchbinder R, Woolf AD, March L. PICO. The global burden of other musculoskeletal disorders: estimates from the Global Burden of Disease 2010 study. *Ann Rheum Dis* 2014;73:1462-9.

10. Sampson UK, Fowkes FG, McDermott MM, Criqui MH, Aboyans V, Norman PE, Forouzanfar MH, Naghavi M, Song Y, Harrell FE Jr, Denenberg JO, Mensah GA, Ezzati M, Murray C. Global and regional burden of death and disability from peripheral artery disease: 21 world regions, 1990 to 2010. *Glob Heart* 2014;9:145-58.e21.

11. Lee CD, Folsom AR, Blair SN. Physical activity and stroke risk: a meta-analysis. *Stroke* 2003;34:2475-81.

12. Laaksonen DE, Lindström J, Lakka TA, Eriksson JG, Niskanen L, Wikström K, Aunola S, Keinänen-Kiukaanniemi S, Laakso M, Valle TT, Ilanne-Parikka P, Louheranta A, Hämäläinen H, Rastas M, Salminen V, Cepaitis Z, Hakumäki M, Kaikkonen H, Härkönen P, Sundvall J, Tuomilehto J, Uusitupa M; Finnish diabetes prevention study. Physical activity in the prevention of type 2 diabetes: the Finnish diabetes prevention study. *Diabetes* 2005;54:158-65.

For peer review only

13. Lee IM. Physical activity and cancer prevention--data from epidemiologic studies. *Med Sci Sports Exerc* 2003;35:1823-7.

14. Kersten RF, Stevens M, van Raay JJ, Bulstra SK, van den Akker-Scheek I. Habitual physical activity after total knee replacement. *Phys Ther* 2012;92:1109-16.

15. National Guideline Centre (UK). Evidence review for information needs: Joint replacement (primary): hip, knee and shoulder: Evidence review A. London: National Institute for Health and Care Excellence (UK); 2020.

16. French DP, Olander EK, Chisholm A, Mc Sharry J. Which behaviour change techniques are most effective at increasing older adults' self-efficacy and physical activity behaviour? A systematic review. *Ann Behav Med* 2014;48:225-34.

17. O'Dwyer T, Monaghan A, Moran J, O'Shea F, Wilson F. Behaviour change intervention increases physical activity, spinal mobility and quality of life in adults with ankylosing spondylitis: a randomised trial. *J Physiother* 2017;63:30-9.

18. Burkow TM, Vognild LK, Johnsen E, Bratvold A, Risberg MJ. Promoting exercise training and physical activity in daily life: a feasibility study of a virtual group intervention for behaviour change in COPD. *BMC Med Inform Decis Mak* 2018;18:136.

19. Boulton E, Hawley-Hague H, French DP, Mellone S, Zacchi A, Clemson L, Vereijken B, Todd C. Implementing behaviour change theory and techniques to increase physical activity and prevent functional decline among adults aged 61-70: The PreventIT project. *Prog Cardiovasc Dis* 2019;62:147-56.

20. Samdal GB, Eide GE, Barth T, Williams G, Meland E. Effective behaviour change techniques for physical activity and healthy eating in overweight and obese adults; systematic review and meta-regression analyses. *Int J Behav Nutr Phys Act* 2017;14:42.

21. Smith TO, Parsons S, Fordham B, Ooms A, Dutton S, Hing C, Barber VS, Png ME, Lamb S; PEP-TALK Trial Collaborators. Behaviour change physiotherapy intervention to increase physical activity following hip and knee replacement (PEP-TALK): study protocol for a pragmatic randomised controlled trial. *BMJ Open* 2020;10:e035014.

22. Department of Health. 2013. General Practice Physical Activity Questionnaire. Accessed on 21.02.2017. Available at: https://www.gov.uk/government/publications/general-practice-physical-activity-questionnaire-gppaq

23. Charlson ME, Pompei P, Ales KL, MacKenzie CR. A new method of classifying prognostic comorbidity in longitudinal studies: development and validation. *J Chronic Dis* 1987;40:373-83.

24. Charlson ME, Charlson RE, Briggs W, Hollenberg J. Can disease management target patients most likely to generate high costs? The impact of comorbidity. *J Gen Intern Med* 2007;22:464-9.

25. Hodkinson HM. Evaluation of a mental test score for assessment of mental impairment in the elderly. *Age Ageing* 1972;1: 233–8.

26. Zahiri CA, Schmalzried TP, Szuszczewicz ES, Amstutz HC. Assessing activity in joint replacement patients. *J Arthroplasty* 1998;13:890–5.

For peer review only - http://bmjopen.bmj.com/site/about/guidelines.xhtml

27. Binkley JM, Stratford OW, Lott SA, Riddle DL. The Lower Extremity Functional Scale (LEFS): Scale development, measurement properties, and clinical application. *Phys Ther* 1999;79:371-83.

28. Dawson J, Fitzpatrick R, Carr A, Murray D. Questionnaire on the perceptions of patients about total hip replacement. *J Bone Joint Surg Br.* 1996;78:185-90.

29. Dawson J, Fitzpatrick R, Murray D, Carr A. Questionnaire on the perceptions of patients about total knee replacement. *J Bone Joint Surg Br.* 1998;80:63-9.

30. Schwarzer R, Jerusalem M. Generalized Self-Efficacy scale. In J. Weinman, S. Wright, & M. Johnston, Measures in health psychology: A user's portfolio. Causal and control beliefs 1995, pp. 35-7.

31. Miller RP, Kori S, Todd D. The Tampa Scale: a measure of kinesiophobia. *Clin J Pain* 1991;7:51–2.

32. Zigmond AS, Snaith RP. The hospital anxiety and depression scale. *Acta Psychiatrica Scandinavica* 1983;67:361–70.

33. EuroQol Group E (1990) EuroQol—a new facility for the measurement of health-related quality of life. *Health Policy* 16:199–208.

34. SooHoo NF, Li Z, Chenok KE, Bozic KJ. Responsiveness of patient reported outcome measures in total joint arthroplasty patients. *J Arthroplasty* 2015;30:176-91.

35. Ooms A, Dutton S, Parsons S, Fordham B, Hing C, Lamb SE, Smith TO. Statistical analysis plan for a pragmatic phase III randomised controlled trial examining behaviour change physiotherapy intervention to increase physical activity following hip and knee replacements: the PEP-TALK Trial. *Trials* 2021;22:467.

36. Schulz KF , Altman DG , Moher D , et al. CONSORT 2010 statement: updated guidelines for reporting parallel group randomised trials. *BMC Med* 2010*;8:*18.

37. Fawaz WS, Masri BA. Allowed activities after primary total knee arthroplasty and total hip arthroplasty. *Orthop Clin North Am* 2020;51:441-52.

38. Luna IE, Kehlet H, Wede HR, Hoevsgaard SJ, Aasvang EK. Objectively measured early physical activity after total hip or knee arthroplasty. *J Clin Monit Comput* 2019;33:509-22.

39. Hawke LJ, Shields N, Dowsey MM, Choong PFM, Taylor NF. Physical activity levels after hip and knee joint replacement surgery: an observational study. *Clin Rheumatol* 2019;38:665-74.

40. Witjes S, Gouttebarge V, Kuijer PP, van Geenen RC, Poolman RW, Kerkhoffs GM. Return to sports and physical activity after total and unicondylar knee arthroplasty: a systematic review and meta-analysis. *Sports Med* 2016;46:269-92.

41. Hamel MB, Toth M, Legedza A, Rosen MP. Joint replacement surgery in elderly patients with severe osteoarthritis of the hip or knee: decision making, postoperative recovery, and clinical outcomes. *Arch Intern Med* 2008;168:1430-40.

42. Ethgen O, Bruyère O, Richy F, Dardennes C, Reginster JY. Health-related quality of life in total hip and total knee arthroplasty. A qualitative and systematic review of the literature. *J Bone Joint Surg Am* 2004;86:963-74.

For peer review only

43. Smith TO, Dainty JR, Clark EM, Whitehouse MR, Price AJ, MacGregor AJ. Demographic and geographical variability in physiotherapy provision following hip and knee replacement. An analysis from the National Joint Registry for England, Wales, Northern Ireland and the Isle of Man. *Physiotherapy* 2020;106:1-11.

44. Garriga C, Leal J, Sánchez-Santos MT, Arden N, Price A, Prieto-Alhambra D, Carr A, Rangan A, Cooper C, Peat G, Fitzpatrick R, Barker K, Judge A. Geographical variation in outcomes of primary hip and knee replacement. *JAMA Netw Open* 2019;2:e1914325.

45. Fleischman AN, Crizer MP, Tarabichi M, Smith S, Rothman RH, Lonner JH, Chen AF. 2018 John N. Insall Award: Recovery of knee flexion with unsupervised home exercise is not inferior to outpatient physical therapy after TKA: a randomized trial. *Clin Orthop Relat Res* 2019;477:60-9.

46. Hamilton DF, Beard DJ, Barker KL, Macfarlane GJ, Tuck CE, Stoddart A, Wilton T, Hutchinson JD, Murray GD, Simpson AHRW; TRIO investigators. Targeting rehabilitation to improve outcomes after total knee arthroplasty in patients at risk of poor outcomes: randomised controlled trial. *BMJ* 2020;371:m3576.

47. Barker KL, Room J, Knight R, Dutton S, Toye F, Leal J, Kenealy N, Maia Schlüssel M, Collins G, Beard D, Price AJ, Underwood M, Drummond A, Lamb S; CORKA Trial group. Home-based rehabilitation programme compared with traditional physiotherapy for patients at risk of poor outcome after knee arthroplasty: the CORKA randomised controlled trial. *BMJ Open* 2021;11:e052598.

48. Smith TO, Kamper SJ, Williams CM, Lee H. Reporting of social deprivation in musculoskeletal trials: An analysis of 402 randomised controlled trials. *Musculoskeletal Care* 2021;19:180-5.

49. Smith TO, Belderson P, Dainty JR, et al. Impact of COVID-19 pandemic social restriction measures on people with rheumatic and musculoskeletal diseases in the UK: a mixed-methods study. *BMJ Open* 2021;11:e048772.

50. Brady SM, Fenton SAM, Metsios GS, Bosworth A, Duda JL, Kitas GD, Veldhuijzen van Zanten JJCS. Different types of physical activity are positively associated with indicators of mental health and psychological wellbeing in rheumatoid arthritis during COVID-19. *Rheumatol Int* 2021;41:335-44.

For peer review only

Figure 1: CONSORT Flow-Chart

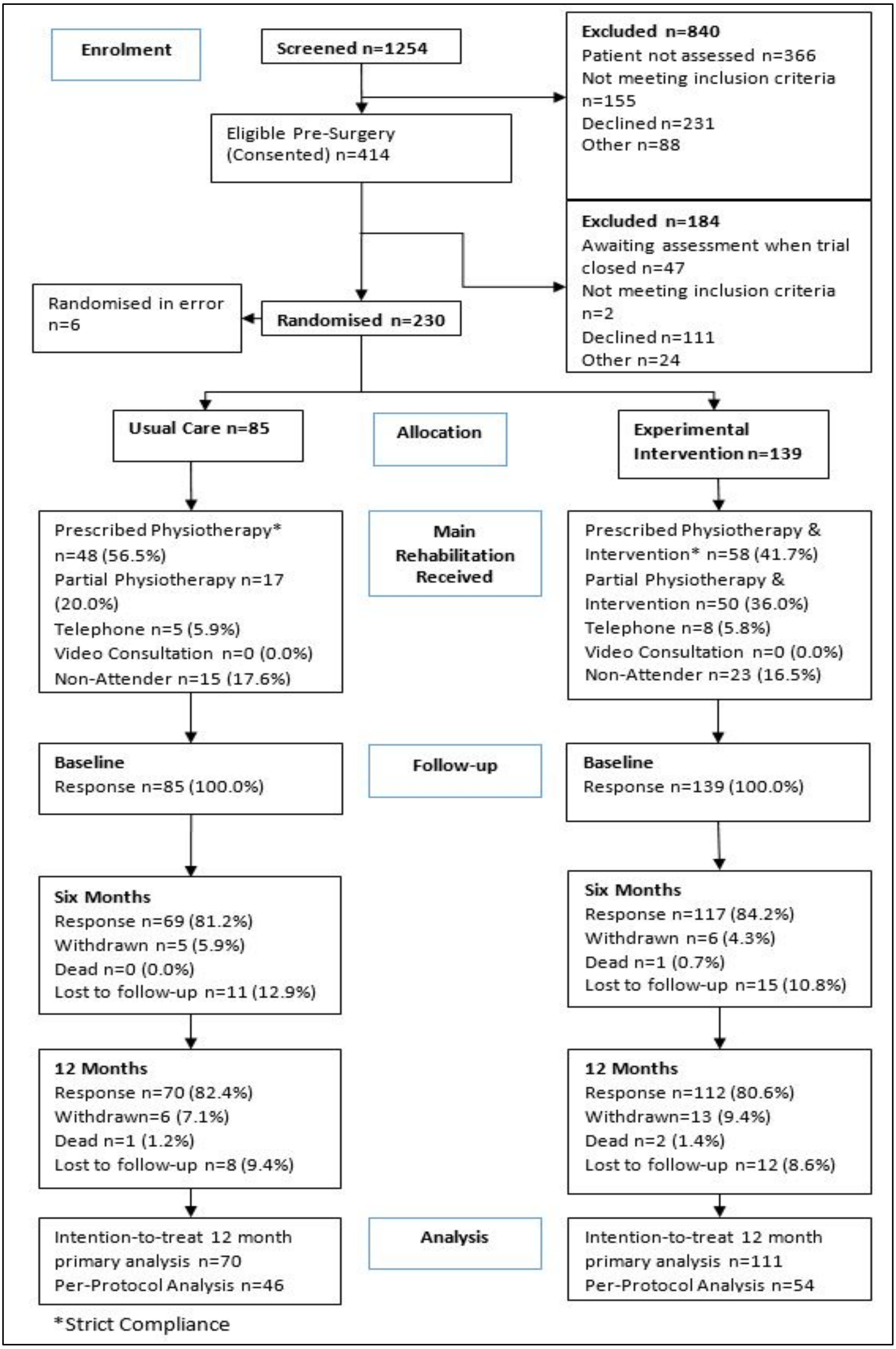

Figure 2: UCLA Activity Score boxplots

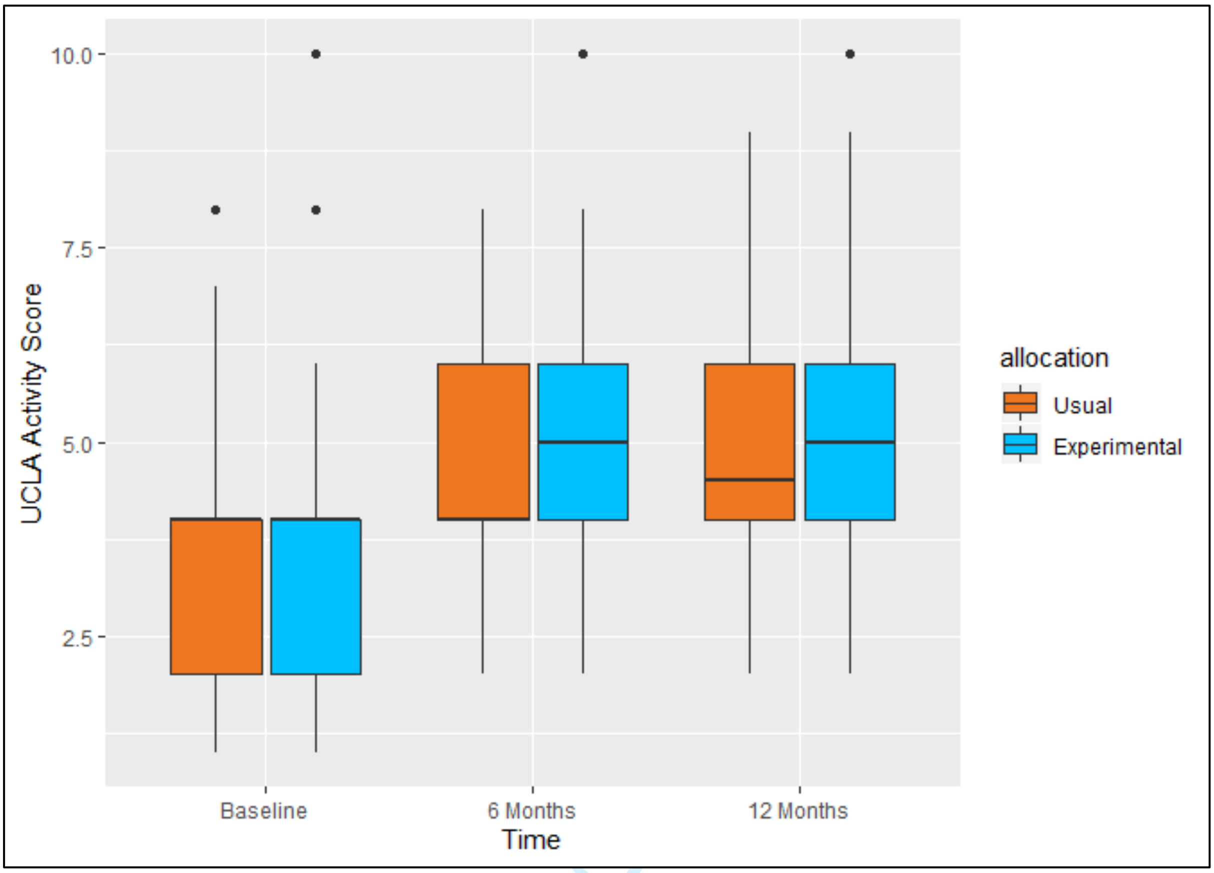

For peer review only

Supplementary File 1: PEP-TALK programme intervention outline and development

Background

Total hip (THR) and knee replacement (TKR) are two highly successful orthopaedic procedures which reduce pain for people with osteoarthritis (1-2). Over 206,000 THRs and TKRs were performed in the UK in 2018 (1). Approximately 90% of patients report significant improvements in pain and physical function after three to 12 months (2-3). However medical co-morbidities are common in this population. These include hypertension (56%) (4) and cardiovascular disease (20%) (5), diabetes (16%) (5) and multi-joint pain (57%) (4). Twenty-seven percent of people who undergo joint replacement have three or four comorbidities (5). These have a significant negative impact on both health-related quality of life and societal burden (6-7).

Historically, it has been assumed that people are more active following TKR and THR through the amelioration of their joint pain (8). However physical activity, for most patients, remains the same from pre- to post-operatively, and in some instances declines (8-9). Physical activity can significantly reduce the symptoms associated with common comorbidities (10). Participating in regular physical activity can decrease the risk of cardiovascular disease by 52% (11), diabetes by 65% (12) and some cancers by 40% (13). It can reduce all-cause mortality by 33% and cardiovascular mortality by 35% (14). Supporting people to be more physically active can improve patient health and decrease economic burden on health services.

A systematic review identified several barriers and facilitators associated with physical activity following TKR and THR (9). From this, four key mechanisms of action were identified for targeting. These were:

(1) Psychoeducation (knowledge/information) to increase self-efficacy.
(2) Reducing fear-avoidant behaviours in response to unhelpful beliefs about activity jeopardising recovery or damaging the implant.
(3) Providing opportunities for personal enjoyment of the physical activity.
(4) Enabling social contact, peer-support and advice from previous patients (encouraging positive coping behaviours).

Systematic reviews of behaviour change interventions have identified that those with a theoretical basis are more effective than those without (15-16). The Social Cognitive Theory (SCT) (17) has been commonly used to understand physical activity behaviour in older adults. The theory targets self-efficacy, goals, outcome expectations and socio-structural factors. Bandura (17) hypothesises that behaviour (physical activity level) is influenced by bi-directional relationships with personal factors (cognitive, emotional and physical) and environment. The cognitive behavioural approach uses techniques to identify and target unhelpful thoughts and behaviours in order to produce adaptive thoughts, behaviours, emotions and physiological responses.

Using the SCT framework, we reviewed evidence on the effectiveness of behaviour change techniques for older adults to improve physical activity. These were then compared to the systematic review regarding patients' perspectives post-TKR/THR (9) to for the four key SCT targets outlined below.

1. *Self-Efficacy: A person's belief in their own ability to perform a behaviour*

<u>General self-efficacy:</u> Quantitative and qualitative systematic reviews examining barriers and facilitators for older adults to increase physical activity have identified specific beliefs which could reduce an individual's general self-efficacy (9, 19-21). These include: stigma, body image (20) and ageing stereotypes (19). Unhelpful beliefs can be identified and explored using cognitive behavioural techniques to increase self-efficacy. The evidence also identified tools to increase general self-efficacy

For peer review only

which include the credibility of instructors and the information/physical activity tasks they provide (19-20, 22).

Self-efficacy to cope with barriers: Barrier identification and problem-solving are two key behaviour change techniques previously identified from the literature. Barriers can be socio-structural such as lack of access/convenience of facilities (20). Whilst these types of barriers cannot be changed by the PEP-TALK intervention, we can facilitate problem-solving strategies to help overcome such barriers.

The intervention programme will be a group-based rolling programme consisting of people in different stages of their behaviour change process. Peers may suggest ideas to other members in addition to ideas from instructors (20). Barriers may also be cognitive beliefs such as a fear of increasing physical activity in case of damaging the implant (9). These beliefs can be targeted with cognitive behavioural strategies.

Task efficacy: Previous literature has consistently reported that if someone has struggled with performing physical activity in the past, they will understandably have poor self-efficacy for performing physical activity tasks in the future (9, 23-24). We will target this by encouraging supportive environments to try exercises with physiotherapists (22), vicariously learning from other patients following THR or TKR (23) and tailored exercises to meet their individual needs (19). This should theoretically increase self-efficacy and the likelihood of greater physical activity engagement (17).

Somatic and emotional states influence self-efficacy (17). Experiencing stress/tension (emotional), fatigue and pain (somatic) can be interpreted by individuals as an indication that they cannot or should not be active. This consequently lowers their self-efficacy. This will be targeted with psychoeducation regarding relationships between mood and pain to physical activity. Conversely positive mood often increases self-efficacy. French et al (23) identified rewards contingent on attempts to perform the behaviour to be a key behaviour change technique for older adults in increasing physical activity. In our intervention, we will ensure participants are praised or rewarded for attempting to achieve their behavioural goal.

2. *Goals*

The SCT suggests that identifying proximal and distal goals are key to behaviour change (17). While this may be the case for younger adults, in older adults and individuals following THR or TKR specifically, goal-setting has consistently shown not to be a useful technique and not acceptable (9, 22-23). French et al (23) proposes two explanations regarding this change. Firstly, with age, cognitive process of executive functioning (planning, attentional capacity, inhibition of responses or novel actions) decreases to reduce abilities to self-regulate with goal-setting. Secondly, at this life stage, achieving set goals and normative comparison is not as pertinent as it is in earlier life. Therefore, we shall not include goal-setting in this intervention.

3. *Outcome Expectation*

While the motivation for this intervention may be to increase physical activity for improved health, evidence suggests that health improvement is not the salient outcome for older adults following THR or TKR. This population appear more interested in the social aspect and the enjoyment through physical activity (9). The Socioemotional Selectivity Theory (25) is a life-span theory of motivation which suggests that as people age, motivation is influenced more by positive, emotionally meaningful goals and activities and less so by normatively defined goals of health. This is extended by Devereux-Fitzgerald's (22) model of the interplay of factors of acceptability to physical activity interventions for older adults. They identified that interventions which provide the most enjoyment and meaningful value (e.g. social interactions) are the most acceptable (22). Our intervention aims to identify what is meaningful and valuable to participants by consistently asking them to reflect on open questions such as "what do you want to gain from attending this group? What are you enjoying most?" then tailoring

For peer review only

why and how to perform physical activity to meet these needs. We will also consider these factors when discussing maintenance and continuation of increased physical activity, identifying activities which are fun and enjoyable for each person. This can be aided by ideas generated from group members who may be at different stages of the behaviour change process.

4. *Socio-Structural Factors*

Although socio-structural factors are key to the SCT, these are aspects which we cannot change from an intervention perspective. However, we can identify modifiable factors and use problem-solving techniques to overcome barriers or find alternatives options. For example, a patient explains there is no safe pavement to walk along from their house to the shops and consequently the patient always drives. The group could offer local knowledge solutions, perhaps there is a nearby bus which can take the patient into a part of the town with good walkways. If the patient does not want to catch the bus then this belief could be explored to further understand the perceived barrier (lack of knowledge of the bus routes, perceived financial cost). This technique was identified as a key behaviour change technique for older adults in increasing physical activity (23).

In summary, while there are four key constructs in the SCT, we anticipate that self-efficacy is the key construct to target for change. A key barrier, specific to this population, to improve self-efficacy could be targeting the personal beliefs regarding fear of damaging the implant or re-injury (9). We prioritise targeting self-efficacy and fear avoidance as they are two key constructs that will change as a result of our behaviour change techniques to mediate and improve physical activity within this population.

Intervention development

The SCT provides an in-depth psychological model of why people do or do not perform behaviours. These psychological models of behaviour have been successfully synthesised into a pragmatic framework called the Capability, Opportunity, Motivation – Behaviour (COM-B) model (26). To produce the most effective behaviour change intervention, the evidence has been mapped on biopsychosocial determinants of physical activity levels post-THR/TKR from the SCT onto the COM-B model for behaviour change (*as presented in figure below*). This activity is summarised in the table below.

Capability Opportunity Motivation model of Behaviour (COM-B; Michie et al, 2014)

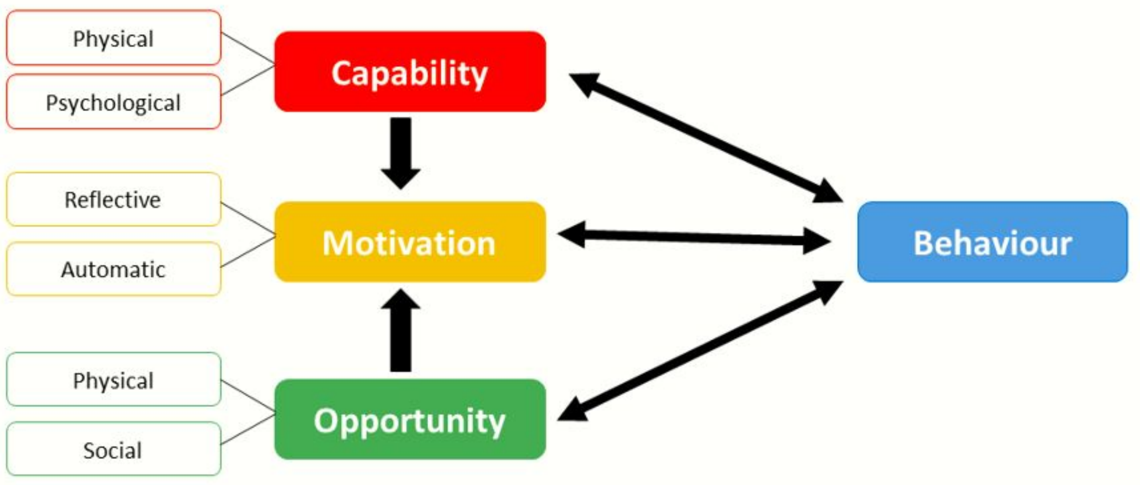

Figure 1: The COM-B model of behaviour change (Michie et al, 2014)

For peer review

Mapping of the COM-B domains against the PEP-TALK SCT targets.

COM-B Model Component	Domain	Activity
Capability	Physical capability	Physiotherapeutic rehabilitation to increase the patient's capability to perform physical activities i.e. specific exercises to reduce stiffness and pain
	Psychological capability	Using cognitive behavioural techniques to increase self and task efficacy beliefs.
Opportunity	Physical opportunity	Identifying and developing problem solving techniques to overcome physical barriers to physical activity i.e. walking to a bus stop further away from the house.
	Social opportunity	Fostering solutions of how to perform physical activities in a social context i.e. communal gardening.
Motivation	Reflective	Using the PEP-TALK discussions to consciously weigh up the individual's pros and cons to performing more physical activity.
	Automatic	Developing active participation from the PEP-TALK participants to encourage linking physical activity into their daily life routine behaviours. Repetition of physically active behaviours can then become linked to everyday activities and will hopefully form into healthy habits which consistently remind, prompt and foster long-term motivation to increase physical activity.

A large proportion of the research into behaviour change techniques to increase physical activity in older adults is based on short-term (less than 12-month follow-up) data. By combining this well-developed model of intervention development, with the SCT model, and specific cognitive behavioural techniques which we have used successfully in previous interventions to increase physical activity (27-28), we hope to produce a sustained behaviour change.

Acceptability of the intervention

The evidence repeatedly recommends listening to what participants want from the intervention (20, 22-23). We aim to learn from participants what their motivations are and what will make the intervention acceptable (22).

We aim to integrate the four analytical themes from the systematic review (9) into the intervention development:

(1) Psychoeducation
(2) Reducing fear-avoidant behaviours in response to unhelpful beliefs i.e. "physical activity will damage my joint replacement"
(3) Providing opportunities for personal enjoyment of the physical activity.
(4) Enabling social contact, peer-support and advice from previous patients

To enhance the acceptability of the intervention, the social enjoyment of the group will be encouraged for making friends, as this is highly valued in older adults. Another aspect is the individual variation in the intervention exercises. This will be overcome by providing one-to-one attention, going at the participant's own pace and making the credibility of the physiotherapist and the intervention content explicit to meet the expectations and needs of older adults.

For peer review only

Hypothesised mediation pathway

From the literature and from our previous models of behaviour change to increase physical activity combined with physiotherapy interventions (27-28), we have developed a model of mediation. We propose that our intervention will increase physical activity levels by increasing self-efficacy and reducing fear avoidance. The pathway of mediation is outlined in the figure below. We are not specifically targeting mental health or pain experience with our intervention, but we are sensitive to monitor if increasing physical activity has a positive effect on these variables.

<u>Proposed pathway of mediation for the PEP-TALK programme</u>

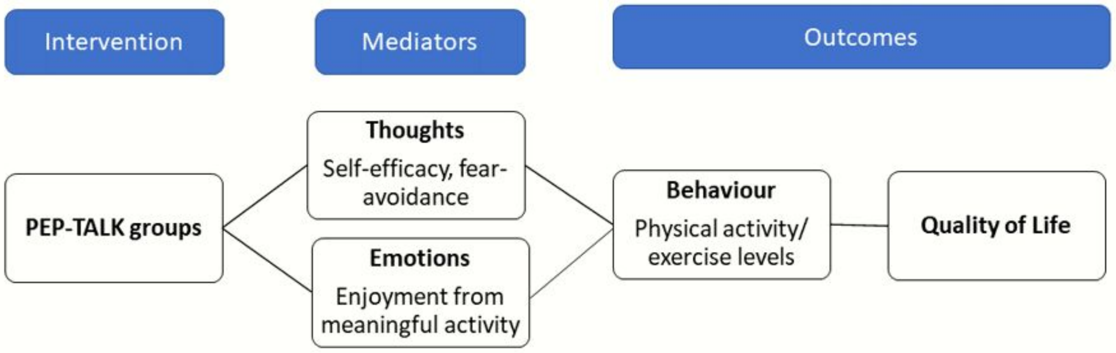

Figure 2: Proposed mediation pathway for PEP-TALK

The PEP-TALK intervention

The PEP-TALK behaviour change group will be delivered face-to-face by one physiotherapist to a group for 30 minutes. Immediately after finishing the 'talking' session the participants will begin their THR/TKR rehabilitation exercises for another 30 minutes. During the exercise session the physiotherapist will continue to talk to the participants. Asking them what they are thinking/feeling when they perform the exercises; encouraging them to reflect on their experience of pain if they encounter this. Using reflective questions to help the participants solve any barriers they encounter whilst performing the exercises. These informal encounters are used to put the theory discussed in the 'talking' group into real life practice.

At the beginning of the PRP-TALK course, intervention participants receive a printed workbook which includes information summarising the techniques, sharing examples and includes homework tasks. The homework tasks are essential for participants to practice translating the behaviour change techniques discussed in the groups, into their real lives, Reflecting on their experiences, thoughts, feelings and behaviours.

The PEP-TALK intervention, in total, lasts for one hour. The control participants only attend the THR/TKR rehabilitation exercise class, which lasts 30 minutes. The control THR/TKR exercise class includes the same physical exercises as prescribed in the intervention group's exercise class but without any of the behaviour change discussion.

<u>Methods of Delivery</u>

The PEP-TALK sessions will be delivered by a physiotherapist trained in the PEP-TALK intervention. The training consists of the PEP-TALK manual outlining the theories of behaviour change, principles of the cognitive behavioural approach, the identified barriers and facilitators to physical activity and exercises. Following this, physiotherapists will attend a one-day training session delivered by a member of the PEP-TALK programme development team (BF, ZH, TS). In this, physiotherapists will discuss the theoretical underpinning of the programme and be provided with case studies and

examples of how the PEP-TALK intervention is designed to be prescribed, and discussion on potential threats to fidelity. We will role play some patient-physiotherapist interactions to provide practical experiences of the intervention in a supportive environment. The trainers will assess how well physiotherapists follow the intervention and will acknowledge any deviations to correct practice.

The PEP-TALK intervention is delivered immediately prior to an exercise group. By timing the interventions with the group discussion first, participants will immediately action and re-enforce the encouragement for physical activity participations through exercising. We have stipulated a maximum PEP-Talk group size of 12 participants to prevent participants from becoming lost in the group and to parallel the standard usual care group size.

A group rather than a one-to-one approach has the advantage of enabling collaborative and vicarious learning, which can improve self-efficacy regarding their goal behaviour (i.e. increased physical activity), whilst also providing lower unit-costs of delivery (29). The principles underpinning this derive from Bandura et al's (17) SCT regarding vicarious learning where learning is proposed to not be acquired through direct experience but by observing other people's actions and consequences (modelling). Secondly, the principles of social cognitive development theory (30) are adopted where knowledge is acquired through guided collaboration with people who already have the knowledge. Collaborative learning with 'peers' and expert people (facilitators) helps bridge distance between an individual's level of skill and their potential, the 'zone of proximal development' (30).
Participants and physiotherapists will be encouraged to develop a positive therapeutic alliance where the physiotherapist will generate an environment of trust and belief around the individual challenges the patient has and to support them to overcome these for sustained physical activity adoption. Evidence has highlighted the beneficial impact of a positive therapeutic alliance on outcomes within physiotherapy practice (31). Due to the nature of identifying individual's helpful and unhelpful thoughts, barriers and facilitators and strategies, the intervention has flexibility in the intention to support this approach. Therefore, whilst the intervention described below has key set-elements which form the content of sessions, there will be opportunity for individuals to express meaningful thoughts and experiences to them, thereby personalising the intervention.

Where Delivered

The PEP-TALK behaviour change group and subsequent exercise sessions will be delivered in an out-patient physiotherapy gym environment. Participants will be sat in a circle to facilitate dialogue. Following the 'talking' intervention, participants begin their THR/TKR exercise session. They will perform exercises in exercise stations, monitored by a trained physiotherapist.

The PEP-TALK behaviour change programme consists of six sessions (A-F) delivered as a rolling programme. Once a new participant has been randomised they can join the groups in any session: A, B, C, D, E or F. Consequently, in every session delivered there will be a mixture of participants who have attended 5,4,3,2,1 or 0 previous PEP-TALK sessions. This necessitates a large amount of repetition of the aims and techniques in every session to ensure all members of the group understand the core behaviour change messages. The rolling programme also enables groups to run continuously, minimising a participant's waiting time to join a group.

A treatment log will be completed by the physiotherapists to record the component of what is discussed across the participants group in each of the session.

Group session will be re-enforced with a participant workbook. This provides participants with salient information from each session, and provides them with exercise progressions, an exercise diary, a guide and space to complete homework tasks/record.

Content of PEP-TALK Sessions

For peer review only

Each of the six PEP-TALK sessions (A - F) will follow this structure:

(1) agenda setting – what will be covered in the session
(2) today's session – covering topics which have been demonstrated to impact on physical activity following joint replacement (content listed below)
(3) conclusion – provision of homework and summarising topics covered today and what will be covered in the next session
(4) break - before commencing exercises group session

There is a degree of overlap between sessions to aid reinforcement of ideas and beliefs. This overlap is largely on identification of barriers and discussion of progress for individuals to share. The principles around the six sessions are presented below:

1. <u>"Being Physically Active": Individual's meaning of physical activity and barriers and problem-solving</u>
   a. Exploring what physical activity means to each participant. For example: active living, transport, sports and exercise. Consideration by participants of what proportion of their lives are engaged with each aspect of physical activity and what the harms and benefits are of being inactive and active. Participants consider what potential barriers exists to activity and whether they want to address these barriers.

2. <u>"Gradually increasing physical activity": Under/Over-Activity, Pacing, Graded Activities</u>
   a. In this session individuals will be taught the principles of pacing and graded-activity. Discussion will be centred on an example e.g. cleaning the car and how pacing and graded-activity could be implemented. The concept of determining a 'baseline' of activity will be established. Individuals will be asked to consider what challenges they have to implementing a graded-activity programme in everyday activities. To facilitate this, individuals will be asked to consider another activity and work through how that activity may be paced in the following week.

3. <u>"Should I be doing this?" : Fear-avoidance</u>
   a. This session will focus on education on avoidance of activity and why individuals avoid activities in relation to their recovery and protection of a joint replacement. Consideration will be focused on thoughts which could be challenged particularly in relation to functional tasks such as washing and dressing, walking, sports or home activities. Individuals will consider how fear avoidance is a circular behaviour in relation to 'thoughts', 'feelings', 'actions', 'results' which can reinforce health beliefs around activity avoidance but acknowledging that such a cycle is a normal response given their previous pain. Discussion will be made for individuals to consider how they may overcome these beliefs.

4. <u>"Physical activity benefits" : Emotion and Sleep, Exercise, Social links</u>
   a. Exploration on the benefits of physical activity on emotional health and sleep will form the basis of this session. Individuals will be asked to consider how being less depressed, stressed and sleep deprived and happier with greater social contact can affect their lives. They will consider how these factors inhibit their ability to be more physically active. Discussion will be made on how worry may relate to pain and what strategies they must address this. Individuals will also think about challenging beliefs around failure to be able to complete certain activities and what their own fears are regarding being more or less active.

5. <u>"Can I change how I think?": Worry, Distraction, Unhelpful Thoughts</u>

For peer review only

a. Fears and worries about jeopardizing recovery and long-term joint health will be explored in this session. Individuals will identify and challenge beliefs around physical activity and harm or damage which are unhelpful thoughts. They will explore a 'vicious cycle' notion where unhelpful thinking leads to feeling low, leading to feeling unmotivated, leading to reduced physical activity leading to atrophy which reinforces the unhelpful thought. Individuals will be asked to consider 'answer back thoughts' and strategies to address such unhelpful thoughts and distractions.

6. <u>"Staying active and having fun" : Social and Rewarding</u>
   a. The benefits of physical activity as a reward will be explored in this session. They will be asked to consider what activities they do alone, and which could be done with others, to increase social contact and increase motivation and pleasure from participating in an activity. Individuals will consider potential barriers and strategies to promote and adopt such an approach to everyday activities' which interest them.

<u>Homework Activities</u>

Participants will be supported with skills developed in the group, to work at home on challenges, barriers and facilitators to physical activity behaviour. The 'home-work' after each session will include pacing and behaviour modification, goal-setting to the individual's health and social needs, and techniques to challenge fear avoidant behaviours.

<u>Follow-up Telephone Calls</u>

Three follow-up telephone calls (maximum 20-minute duration) will be undertaken at two, four and six weeks following the last group session. Follow-up telephone calls are an important element of the behaviour change intervention. They will review participant's goals, identifying any barriers to the completion of these goals, and review any 'helpful' and 'unhelpful' thoughts or feelings towards physical activity which may have arisen since the last consultation. Each telephone call will close with the development of longer-term physical activity plans and promotion of empowerment towards physical activity participation using these behavioural principles instilled during the group intervention.

<u>*Adherence and Fidelity*</u>

The PEP-TALK team phone the physiotherapist delivering the intervention group after their first session has been delivered. The aim of this call is to address any problems the physiotherapist may have encountered and for the PEP-TALK team to offer solutions and tips. After the third session has been delivered, a member of the PEP-TALK team visit the site and observe a PEP-TALK behaviour change and exercise session to perform a quality assessment (QA). If there are quality concerns, then the site will receive additional training and another QA visit will be undertaken.

At a participant level, compliance to the PEP-TALK intervention will be arbitrarily met with participants required to attend 70% of the behaviour-change and exercise groups and 66% of the telephone calls.

<u>*Access to the Intervention*</u>

The PEP-TALK intervention manual and work-book will be available on completion of the trial. This can be accessed through the corresponding author.

Conclusions

The development and content of the PEP-TALK intervention has been presented. This addresses key modifiable risk factors to physical inactivity following hip and knee replacement. The effectiveness of

For peer review only

this intervention will now be assessed in the multi-centre, pragmatic, randomised controlled trial (PEP-TALK Trial).

SUPPLEMENTARY FILE 1: REFERENCES

1. National Joint Registry. 2018. ReportsOnline 2018. National Joint Registry UK. Accessed: 22 July 2019. Available at: http://www.njrcentre.org.uk/njrcentre/Healthcare-providers/Accessing-the-data/ReportsOnline
2. Dailiana ZH, Papakostidou I, Varitimidis S, Liaropoulos L, Zintzaras E, Karachalios T, Michelinakis E, Malizos KN. Patient-reported quality of life after primary major joint arthroplasty: a prospective comparison of hip and knee arthroplasty. BMC Musculoskelet Disord. 2015;16:366.
3. Papakostidou I, Dailiana ZH, Papapolychroniou T, Liaropoulos L, Zintzaras E, Karachalios TS, Malizos KN. Factors affecting the quality of life after total knee arthroplasties: a prospective study. BMC Musculoskelet Disord. 2012;13:116.
4. Inacio MC, Pratt NL, Roughead EE, Graves SE. Comparing co-morbidities in total joint arthroplasty patients using the RxRisk-V, Elixhauser, and Charlson Measures: a cross-sectional evaluation. BMC Musculoskelet Disord. 2015;16:385.
5. Peter WF, Dekker J, Tilbury C, Tordoir RL, Verdegaal SH, Onstenk R, Bénard MR, Vehmeijer SB, Fiocco M, Vermeulen HM, van der Linden-van der Zwaag HM, Nelissen RG, Vliet Vlieland TP. The association between comorbidities and pain, physical function and quality of life following hip and knee arthroplasty. Rheumatol Int. 2015;35:1233-41.
6. Martinez-Cano JP, Herrera-Escobar JP, Arango Gutierrez AS, Sanchez Vergel A, Martinez-Rondanelli A. Prospective quality of life assessment after hip and knee arthroplasty: short- and mid-term follow-up results. Arthroplast Today. 2016;3:125-130.
7. Sampson UK, Fowkes FG, McDermott MM, Criqui MH, Aboyans V, Norman PE, Forouzanfar MH, Naghavi M, Song Y, Harrell FE Jr, Denenberg JO, Mensah GA, Ezzati M, Murray C. Smith E, Hoy DG, Cross M, Vos T, Naghavi M, Buchbinder R, Woolf AD, March L. PICO. The global burden of other musculoskeletal disorders: estimates from the Global Burden of Disease 2010 study. Ann Rheum Dis. 2014;73:1462-9.
8. Withers TM, Lister S, Sackley C, Clark A, Smith TO. Is there a difference in physical activity levels in patients before and up to one year after unilateral total hip replacement? A systematic review and meta-analysis. Clin Rehabil. 2017;31:639-650.
9. Smith TO, Latham S, Maskrey V, Blyth A. Patients' perceptions of physical activity before and after joint replacement: a systematic review with meta-ethnographic analysis. Postgrad Med J. 2015;91:483-91.
10. Global Burden of Disease Study 2013 Collaborators. Global, regional, and national incidence, prevalence, and years lived with disability for 301 acute and chronic diseases and injuries in 188 countries, 1990–2013: a systematic analysis for the Global Burden of Disease Study 2013. Lancet. 2015;386:743–800
11. Lee CD, Folsom AR, Blair SN. Physical activity and stroke risk: a meta-analysis. Stroke. 2003;34:2475-81.
12. Laaksonen DE, Lindström J, Lakka TA, Eriksson JG, Niskanen L, Wikström K, et al; Finnish diabetes prevention study. Physical activity in the prevention of type 2 diabetes: the Finnish diabetes prevention study. Diabetes. 2005;54:158-65.
13. Lee IM. Physical activity and cancer prevention--data from epidemiologic studies. Med Sci Sports Exerc. 2003;35:1823-7.
14. Nocon M, Hiemann T, Müller-Riemenschneider F, Thalau F, Roll S, Willich SN. Association of physical activity with all-cause and cardiovascular mortality: a systematic review and meta-analysis. Eur J Cardiovasc Prev Rehabil. 2008;15:239-46.

For peer review only

15. Abraham C and Michie, S. A taxonomy of behaviour change techniques used in interventions. Health Psychology, 2008;27:379-87.
16. Chase J. A. Interventions to increase physical activity among older adults: a meta-analysis. The Gerontologist, 2015;55:706-18.
17. Bandura A. Social foundations of thought and action: A social cognitive theory**.** Prentice-Hall, Inc, 1986.
18. Baranowski T, Anderson C and Carmack C. Mediating variable framework in physical activity intervetnions: How are we doing? how might we do better? Am J Prevent Med. 1998;15;266-97.
19. Arnautovska U, O'Callaghan F, Hamilton K. Applying the Integrated Behavior Change Model to Understanding Physical Activity Among Older Adults: A Qualitative Study. J Sport Exerc Psychol. 2017;39:43-55.
20. Olanrewaju O, Kelly S, Cowan A, Brayne C, Lafortune L. Physical activity in community dwelling older people: a systematic review of reviews of interventions and context. PLoS One. 2016;11:e0168614.
21. Withers TM, Lister S, Sackley C, Clark A, Smith TO. Is there a difference in physical activity levels in patients before and up to one year after unilateral total hip replacement? A systematic review and meta-analysis. Clin Rehabil. 2017;31:639-50.
22. Devereux-Fitzgerald A, Powell R, Dewhurst A, French DP. The acceptability of physical activity interventions to older adults: A systematic review and meta-synthesis. Soc Sci Med. 2016;158:14-23.
23. French DP, Olander EK, Chisholm A, Mc Sharry J. Which behaviour change techniques are most effective at increasing older adults' self-efficacy and physical activity behaviour? A systematic review. Ann Behav Med. 2014;48:225-34.
24. Pekmezi DW, Neighbors CJ, Lee CS, Gans KM, Bock BC, Morrow KM, Marquez B, Dunsiger S, Marcus BH. A culturally adapted physical activity intervention for Latinas: a randomized controlled trial. Am J Prev Med. 2009;37:495-500.
25. Carstensen LL. Motivation for social contact across the life span: a theory of socioemotional selectivity. Nebr Symp Motiv. 1992;40:209-54.
26. Michie S, Atkins L, West R. The Behaviour Change Wheel: A guide to designing Interventions. Silverback Publishing. 2014
27. Lamb SE, Lall R, Hansen Z, Castelnuovo E, Withers EJ, Nichols V, Griffiths F, Potter R, Szczepura A, Underwood M; BeST trial group. A multicentred randomised controlled trial of a primary care-based cognitive behavioural programme for low back pain. The Back Skills Training (BeST) trial. Health Technol Assess. 2010;14:1-253.
28. Lamb 2015. Better outcomes for older people with spinal trouble. Accessed: 24 May 2018. Available at: http://www.isrctn.com/ISRCTN12698674
29. Critchley DJ, Ratcliffe J, Noonan S, Jones RH, Hurley MV. Effectiveness and cost-effectiveness of three types of physiotherapy used to reduce chronic low back pain disability: a pragmatic randomized trial with economic evaluation. Spine 2007;32:1474–81.
30. Vygotsky L. Interaction Between. Learning and Development. In Gauvain and Cole (eds) Readings on the Development of Children. New York: Scientific. American Books. pp. 34-40, 1978.
31. Paulo H. Ferreira, Manuela L. Ferreira, Christopher G. Maher, Kathryn M. Refshauge, Jane Latimer, Roger D. Adams; The therapeutic alliance between clinicians and patients predicts outcome in chronic low back pain. Phys Ther 2013;93:470–8.

For peer review only

Supplementary File 2: Additional results

Pre-Specified Definition of Compliance

Compliance was defined in three nested levels for both randomised groups. These are:

Strict Compliance (as defined in the original Protocol):
Usual Care group
• Attends at least 4 out of 6 physiotherapy sessions

Experimental Intervention group
• Attends at least 4 out of 6 group intervention sessions with a minimum of 3 participants per session
• Received 2 out of 3 follow-up telephone calls

Compliance:
Usual Care group
• Attends at least 4 out of 6 physiotherapy sessions

Experimental Intervention group
• Attends at least 4 out of 6 group intervention sessions with a minimum of 3 participants per session

Attendance:
Usual Care group
• Attends at least 1 out of 6 physiotherapy sessions

Experimental Intervention group

- Attends at least 4 out of 6 group intervention sessions.

Additional Results

A summary of withdrawals is provided in **Supplementary Table 1**.

The primary analysis is performed assuming the data is missing at random (MAR). To assess the MAR assumption, varying scores of the UCLA Activity Score for all time points were imputed where data is missing and these "complete" datasets were reanalysed, using the same mixed effects as used in the primary analysis. For each missing data point, the median value of the group that participant belongs to is imputed and the imputed dataset analysed. The analysis is repeated on a population that has the 60th quantile imputed for one group's missing values and the 40th quantile for the other, then again using the 70th and 30th quantiles, up to 90th and 10th quantiles. The process was repeated but flipping the groups. In total nine sensitivity analyses were performed and the results displayed graphically in **Supplementary Figure 5**. This method used simple imputation of these quantiles, therefore the estimates of the variance will be effected, and so will all p-values and Confidence Intervals reported. **Supplementary Figure 5**shows that there would need to be an implausibility large departure from the missing at random assumption to see a statistically significant result in either direction with a result only being yielded if the 10th and 90th percentiles are imputed into each treatment group. This suggests the result from the primary analysis is robust to missing data and adds support to the findings from the primary analysis.

For peer review only

A sensitivity analysis on the per-protocol population has been performed to assess the internal validity of the trial's primary results. The analysis is based on the same mixed effects analysis model as used for the primary outcome but for the Per-Protocol population as described in the Statistical Analysis Plan.[35] To be considered per-protocol participants must have data on the UCLA Activity Score at 12 months, cannot be "Non-Compliant", cannot be part of the COVID-19 group (as these participants did not complete their intervention per-protocol), did not crossover randomised treatments and did not have any Important protocol deviations reported. Results from this analysis are reported in **Supplementary Table 3**. The per-protocol analysis reinforces the main trial result findings, there is no between group difference.

An analysis on the primary outcome using a reduced version of the primary analysis model, only using person as a random effect has been performed. The results are presented in **Supplementary Table 4**. The results from the reduced model in **Supplementary Table 4** are extremely similar the primary analysis results. The Akaike Information Criterion (AIC) for the primary analysis model was 1,372.47 whereas the AIC for the reduced model was 1,370.84 suggesting a marginally better model fit with centre removed.

All subgroup analyses are on the primary outcome only. Subgroup analyses of the two clinical stratifying variables (type of operation and (THR or TKR), Charlson Comorbidity Index Score (1–3 or ≥ 4)) were performed as well as a subgroup analysis on COVID-19 status (Pre-COVID-19 or COVID-19). These used an extended primary analysis model including an interaction term between treatment and each stratifying variable/COVID-19 status to define the subgroups. These analyses are exploratory, and results should be interpreted with due caution. The results will be presented in a **Supplementary Figure 6**.

Supplementary Figure 1 gives a plot of complication type.

Descriptive statistics for the Generalized Self-Efficacy Scale, Tampa Scale for Kinesiophobia, Hospital Anxiety and Depression Score, EQ-5D-5L Index, EQ-VAS and Numerical Rating Scale for Pain are given by COVID-19 status in **Supplementary Table 5**, no formal analysis is performed. The presentation of these results was pre-specified in the analysis plan and aid in assessing the impact of the COVID-19 pandemic on the trial participants. Results indicate potentially higher levels of anxiety, depression and kinesiophobia at six-months in the COVID-19 population, these apparent differences were not sustained to the 12-month follow-up. Observed self-efficacy scores were lower in the COVID-19 group across all follow-up time points. Other measures did not indicate any noticeable between group difference. These results should be interpreted with great caution due to small sample size, non-random groups, and the exploratory nature of the results.

For peer review only

Supplementary Table 1: Withdrawals summary

	Usual (n=13)	Experimental (n=24)	Total (n=37)
**Treatment Non-Compliance Reason**			
Complete withdrawal from the study and use of data	2	2	4
Withdrawal from intervention and completion of questionnaires	4	11	15
Withdrawal from intervention only	7	11	18
**Withdrawal Time Point**			
6 Months	12	17	29
12 Months	1	7	8

N - number of participants

For peer review only

Supplementary Table 2: Questionnaire returns by treatment group

Time Point	Usual	Experimental	Cumulative missing data	Total with data
Baseline	85 (100.0)	139 (100.0)	0 (0.0)	224 (100.0)
6 Months	69 (81.2)	117 (84.2)	38 (17.0)	186 (83.0)
12 Months	70 (82.4)	112 (80.6)	42 (18.8)	182 (81.2)

All data frequency and (%)

For peer review only

Supplementary Table 3: UCLA Activity Score per-protocol results

Time Point	Usual	Experimental	Mean Difference	
	**n, Mean (SD)**	**n, Mean (SD)**	**Unadjusted**	**Adjusted (95% CI)**
Baseline	n=46, 3.76 (1.51)	n=54, 3.67 (1.65)	-0.09	
6 Months	n=44, 4.91 (1.44)	n=50, 5.18 (1.86)	0.27	0.43 (-0.23,1.08)
12 Months	n=46, 5.04 (1.59)	n=54, 4.83 (1.79)	-0.21	-0.17 (-0.81,0.48)

CI - confidence intervals; N – number of participants; SD – standard deviation

For peer review only

Supplementary Table 4: UCLA Activity Score reduced model (no recruiting centre random effect) results

Time Point	Usual	Experimental	Mean Difference	
	n, Mean (SD)	n, Mean (SD)	Unadjusted	Adjusted (95% CI)
Baseline	n=85, 3.62 (1.52)	n=138, 3.57 (1.57)	-0.06	
6 Months	n=69, 4.77 (1.52)	n=117, 4.97 (1.68)	0.20	0.28 (-0.21,0.76)
12 Months	n=70, 4.87 (1.61)	n=111, 4.84 (1.91)	-0.03	-0.03 (-0.52,0.46)

CI - confidence intervals; N – number of participants; SD – standard deviation

For peer review only

Supplementary Table 5: Descriptive results for selected secondary outcomes by COVID-19 status

	Pre-COVID-19<br>n, Mean (SD)	COVID-19<br>n, Mean (SD)
**Generalized Self-Efficacy Scale**		
Baseline	n=153, 31.82 (5.49)	n=69, 30.90 (5.24)
6 Months	n=112, 33.04 (5.22)	n=44, 31.50 (5.29)
12 Months	n=112, 32.83 (6.27)	n=50, 30.74 (6.13)
**Tampa Scale for Kinesiophobia**		
Baseline	n=153, 40.09 (7.81)	n=68, 39.38 (7.20)
6 Months	n=103, 34.86 (7.79)	n=44, 35.82 (6.62)
12 Months	n=103, 35.57 (8.30)	n=44, 35.80 (6.50)
**Hospital Anxiety and Depression Scale (Overall)**		
Baseline	n=154, 11.99 (6.38)	n=69, 12.83 (7.46)
6 Months	n=110, 8.65 (6.20)	n=46, 9.39 (6.89)
12 Months	n=113, 9.46 (6.95)	n=47, 9.38 (6.60)
**Hospital Anxiety and Depression Scale (Anxiety)**		
Baseline	n=154, 6.19 (3.84)	n=69, 6.71 (4.24)
6 Months	n=112, 4.79 (3.55)	n=46, 5.33 (4.16)
12 Months	n=113, 5.11 (3.75)	n=48, 5.40 (3.95)
**Hospital Anxiety and Depression Scale (Depression)**		
Baseline	n=155, 5.83 (3.40)	n=69, 6.12 (3.95)
6 Months	n=113, 3.89 (3.31)	n=47, 4.09 (3.66)
12 Months	n=115, 4.30 (3.97)	n=48, 4.23 (3.44)
**EQ-5D-5L Index**		
Baseline	n=155, 0.40 (0.24)	n=69, 0.38 (0.28)
6 Months	n=129, 0.68 (0.25)	n=56, 0.69 (0.23)
12 Months	n=128, 0.67 (0.26)	n=55, 0.68 (0.29)
**EQ-VAS**		
Baseline	n=155, 62.34 (21.77)	n=69, 57.55 (23.07)
6 Months	n=130, 71.84 (20.74)	n=55, 75.02 (16.28)
12 Months	n=124, 73.19 (19.85)	n=55, 71.82 (17.62)
**Numerical Rating Scale for Pain**		
Baseline	n=155, 7.09 (1.87)	n=69, 7.10 (1.82)
6 Months	n=115, 3.55 (2.72)	n=47, 3.28 (2.59)
12 Months	n=112, 3.68 (2.88)	n=51, 3.47 (2.87)

N – number of participants; SD – standard deviation

Supplementary Figure 1: Complication type by randomised group

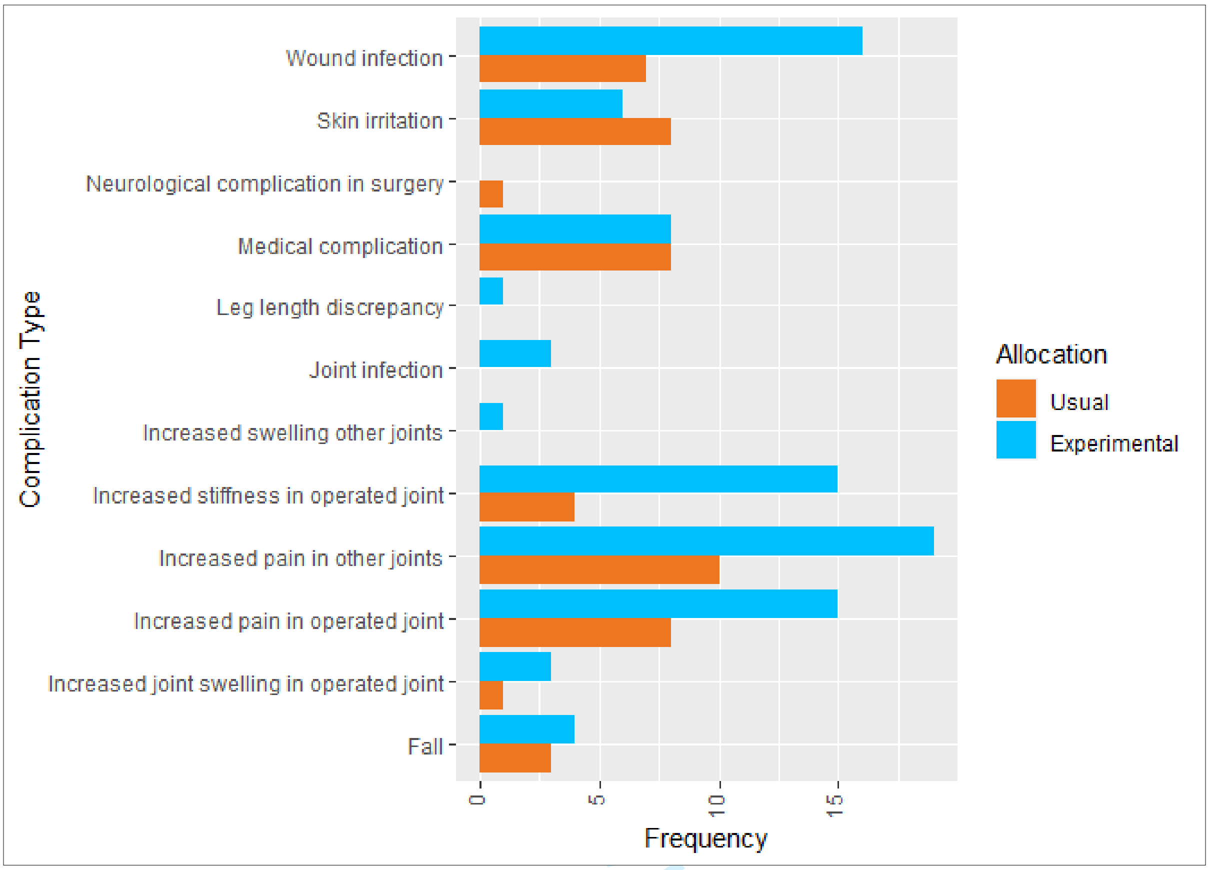

Supplementary Figure 2: Overall compliance by (a) raw frequencies and (b) percentage of randomised group

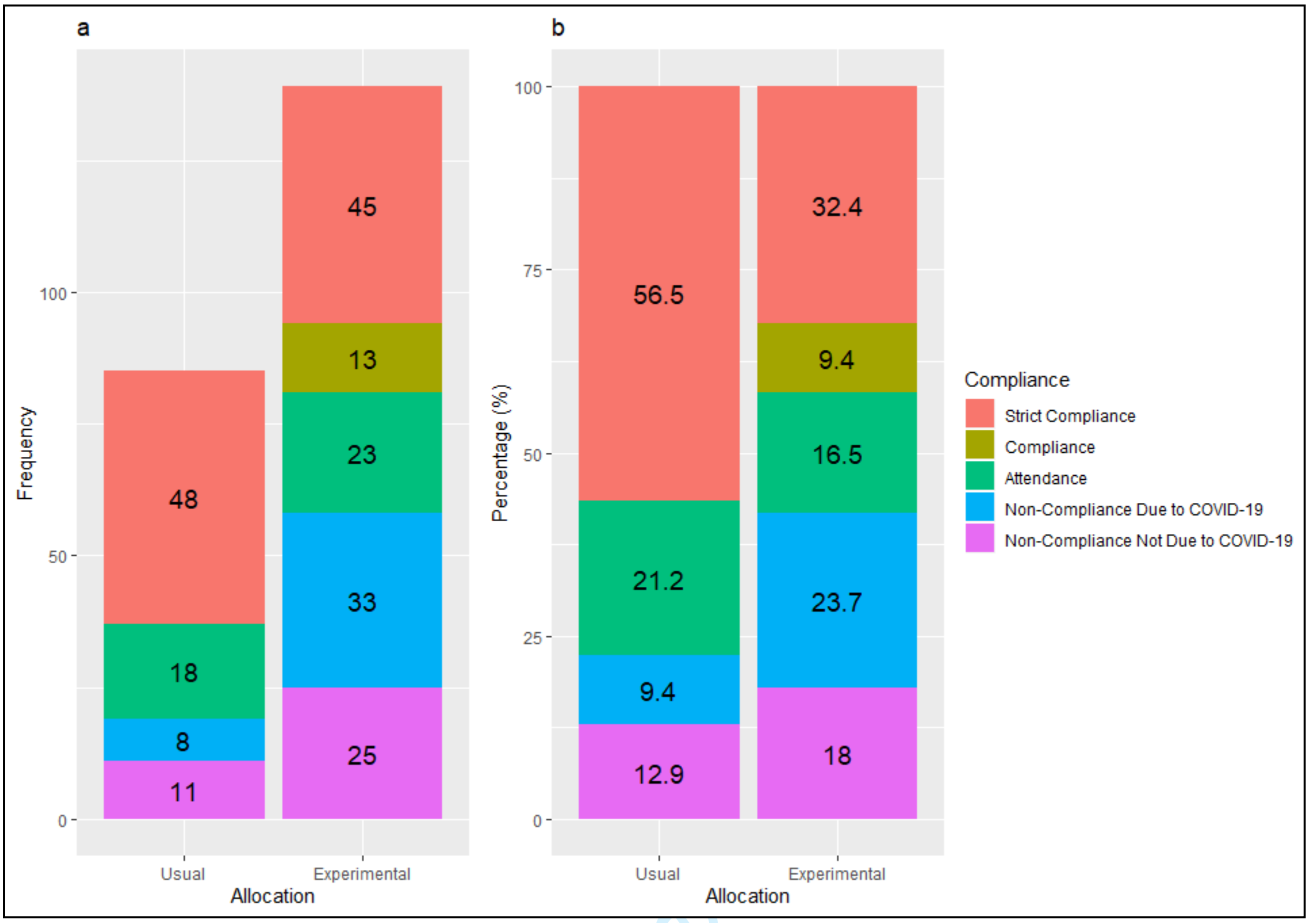

Supplementary Figure 3: Experimental intervention group sizes over time, including change from a randomisation ratio of 1:1 to 2:1

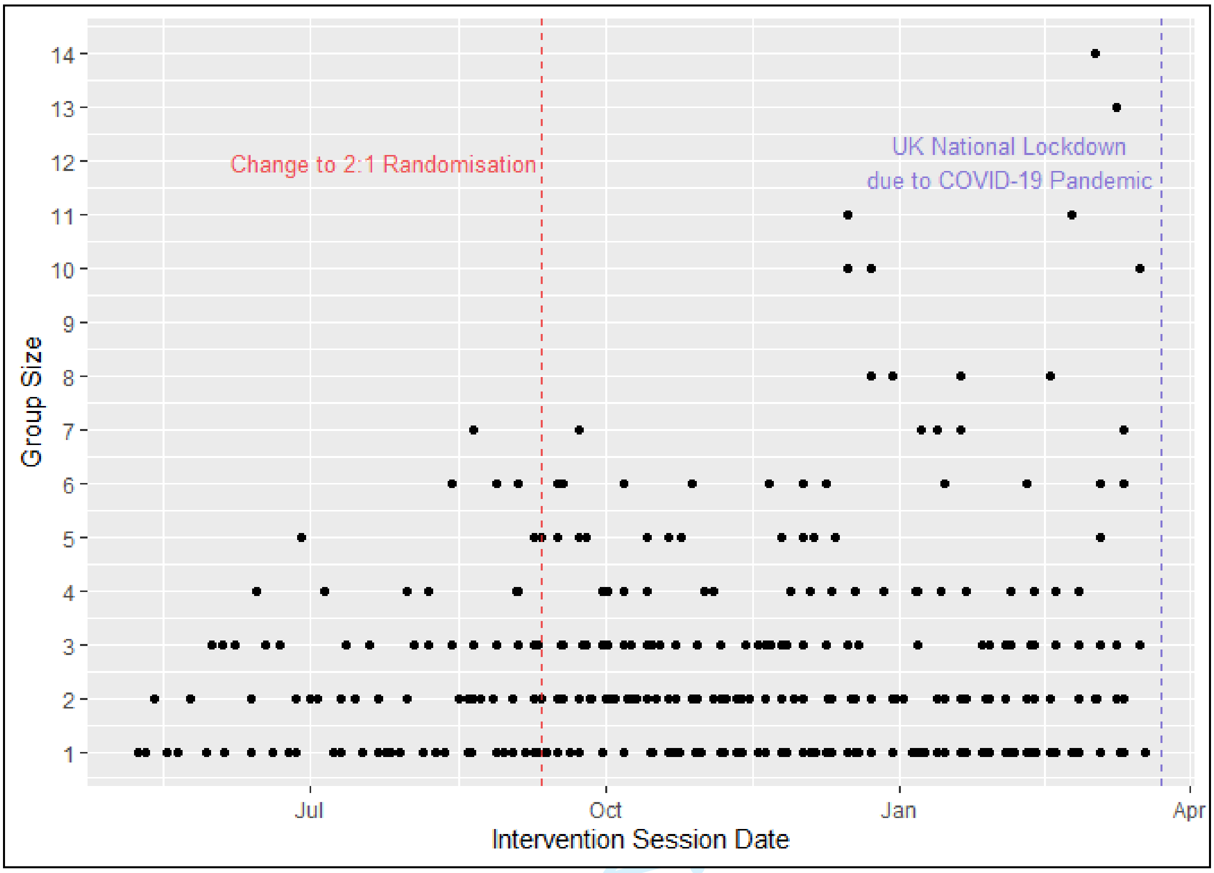

For peer review only

Supplementary Figure 4: Experimental intervention group compliance by COVID-19 group

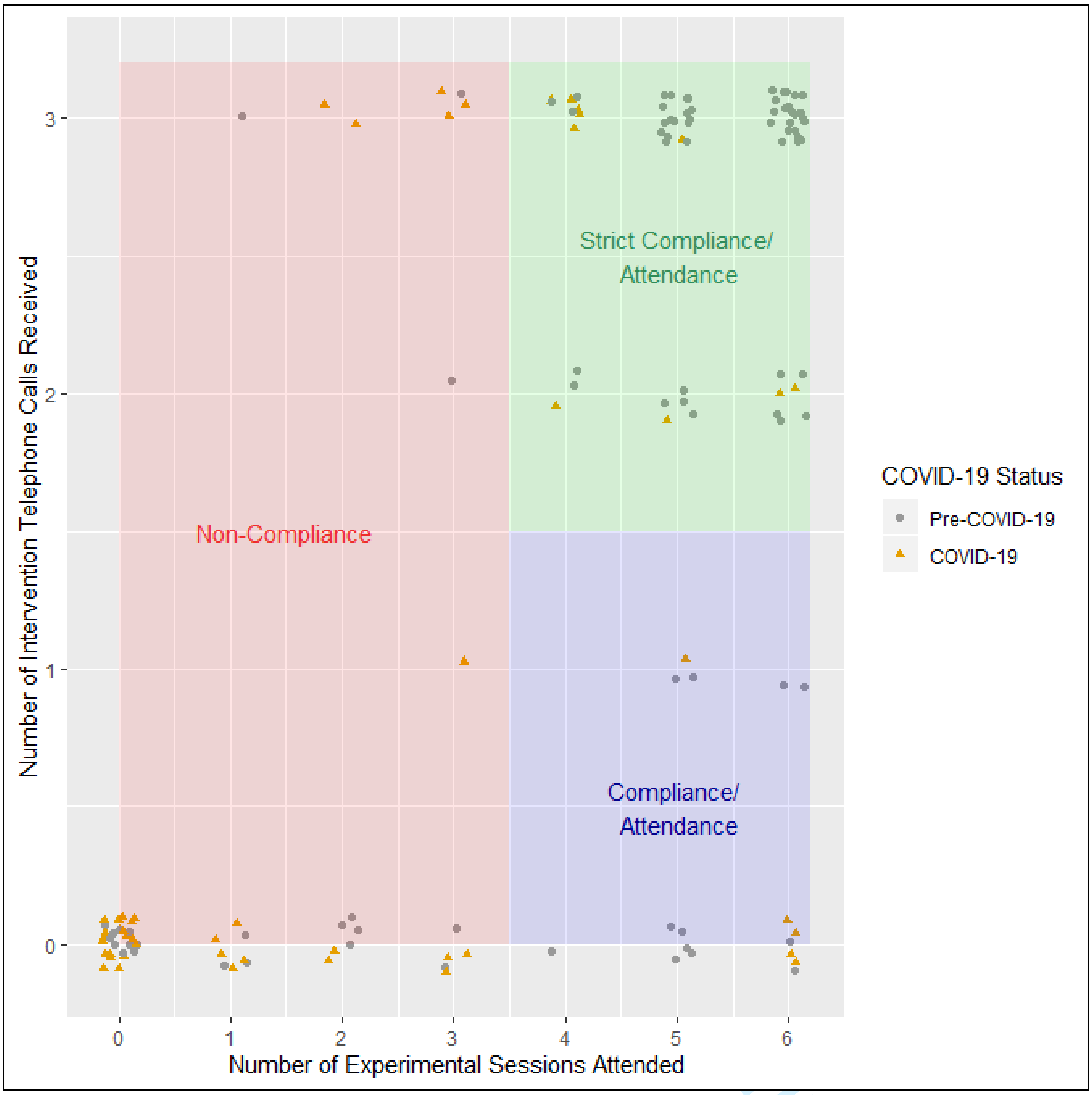

Supplementary Figure 5: 12 month adjusted mean difference UCLA Activity Score for varying imputed quantiles for missing data

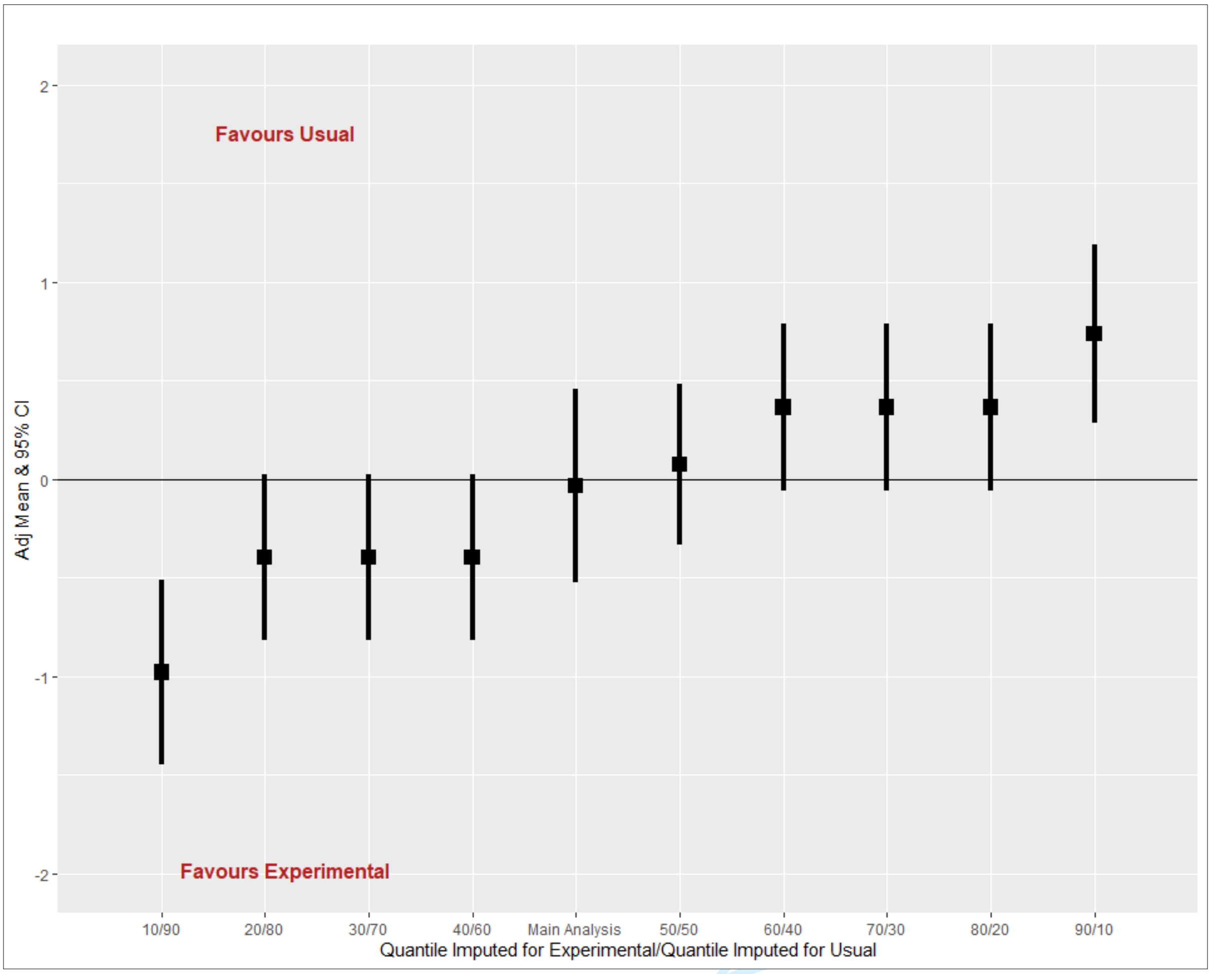

Supplementary Figure 6: Subgroup analyses results

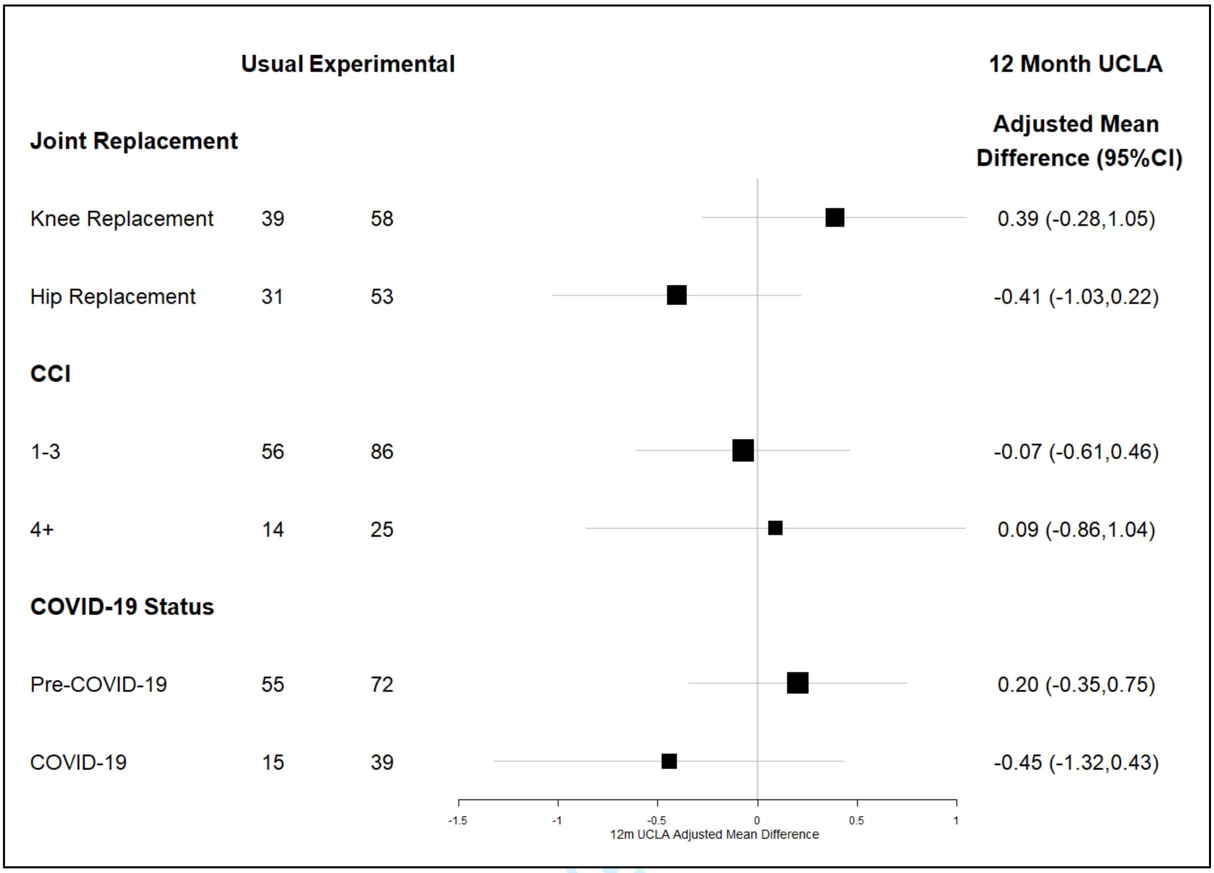

CCI – Charlson Comorbidity Index; CI – Confidence Intervals; UCLA – University fo Los Angeles Activity Score

view only

CONSERVE-CONSORT Checklists

CONSERVE-CONSORT Extension: 22Jan2022 (PEP-TALK Final Report)			
**Item**	**Item Title**	**Description**	**Page No.**
I.	Extenuating Circumstances	Describe the circumstances and how they constitute extenuating circumstances.	Methods, Randomisation and masking Para 1; Statistical Methods, Para 3; Results, Recruitment and participant flow, Para 2; Supplementary File 2
II.	Important Modifications	a. Describe how the modifications are important modifications.	Methods, Randomisation and masking Para 1; Statistical Methods, Para 3;
		b. Describe the impacts and mitigating strategies, including their rationale and implications for the trial.	Methods, Randomisation and masking Para 1; Statistical Methods, Para 3;
		c. Provide a modification timeline.	Results, Recruitment and participant flow, Para 2

For peer review only - http://bmjopen.bmj.com/site/about/guidelines.xhtml

III.	Responsible Parties	State who planned, reviewed and approved the modifications.			Methods, Randomisation and masking, Para 1
IV.	Interim data	If modifications were informed by trial data, describe how the interim data were used, including whether they were examined by study group, and whether the individuals reviewing the data were blinded to the treatment allocation.			No interim analysis performed.

**CONSORT Number and Item**		For each row, if important modifications occurred check "direct impact" and/or "mitigating strategy" and describe the changes in the trial manuscript or supplement. Check "no change" for items that are unaffected in the extenuating circumstance.			**Page No.**
		**No Change**	**Impact***	**Mitigating Strategy****	
1	Title and abstract		X	X	2
2	Introduction	X			4-5
3	Methods: Trial Design	X			5
4	Methods: Participants	X			5
5	Methods: Interventions	X			5-6
6	Methods: Outcomes	X			6
7	Methods: Sample Size	X			7
8-10	Methods: Randomisation	X			7
11	Methods: Blinding	X			7
12	Methods: Statistical methods		X		7-8
13	Results: Participant flow		X	X	8-9
14	Results: Recruitment		X	X	8-9

PEP-TALK_FinalReport_ConserveConsortChecklist_V0.3_04Jan2022

For peer review only - http://bmjopen.bmj.com/site/about/guidelines.xhtml

15	Results: Baseline data	X			9, Table 1
16	Results: Numbers analysed	X			9, Table 2
	Results: Outcomes and estimation		X	X	9-10 Tables 3, 4, 5, 6<br>Figures 3,4,5
18	Results: Ancillary analyses		X	X	10-11, Sup File 2
19	Results: Harms	X			10
20	Discussion: Limitations		X		11-13
21	Discussion: Generalisability		X		11-13
	Other information: Registration	X			2
24	Other information: Protocol	X			Published, ref pg 5
25	Other information: Funding	X			13

*Aspects of the trial that are directly affected or changed by the extenuating circumstance and are not under the control of investigators, sponsor or funder.
**Aspects of the trial that are modified by the study investigators, sponsor or funder to respond to the extenuating circumstance or manage the direct impacts on the trial.

For peer review only - http://bmjopen.bmj.com/site/about/guidelines.xhtml

BMJ Open

Randomised controlled trial of a behaviour change physiotherapy intervention to increase physical activity following hip and knee replacement: the PEP-TALK trial

Journal:	*BMJ Open*
Manuscript ID	bmjopen-2022-061373.R1
Article Type:	Original research
Date Submitted by the Author:	01-Apr-2022
Complete List of Authors:	Smith, Toby O.; University of Oxford, Nuffield Department of Orthopaedics, Rheumatology and Musculoskeletal Sciences; University of East Anglia,<br>Parsons, Scott; University of Oxford, Nuffield Department of Orthopaedics, Rheumatology and Musculoskeletal Disorders<br>Ooms, Alexander; University of Oxford, Nuffield Department of Orthopaedics, Rheumatology and Musculoskeletal Disorders<br>Dutton, Susan; University of Oxford, CSM<br>Fordham, Beth; University of Oxford, NDORMS<br>Garrett, Angela; University of Oxford, Nuffield Department of Orthopaedics, Rheumatology and Musculoskeletal Sciences<br>Hing, Caroline; St George's University Hospitals NHS Foundation Trust<br>Lamb, Sarah; University of Exeter, College of Medicine and Health; University of Oxford, NDORMS
**Primary Subject Heading**:	Rehabilitation medicine
Secondary Subject Heading:	Sports and exercise medicine
Keywords:	Rheumatology < INTERNAL MEDICINE, Adult orthopaedics < ORTHOPAEDIC & TRAUMA SURGERY, Hip < ORTHOPAEDIC & TRAUMA SURGERY, Knee < ORTHOPAEDIC & TRAUMA SURGERY

SCHOLARONE™
Manuscripts

I, the Submitting Author has the right to grant and does grant on behalf of all authors of the Work (as defined in the below author licence), an exclusive licence and/or a non-exclusive licence for contributions from authors who are: i) UK Crown employees; ii) where BMJ has agreed a CC-BY licence shall apply, and/or iii) in accordance with the terms applicable for US Federal Government officers or employees acting as part of their official duties; on a worldwide, perpetual, irrevocable, royalty-free basis to BMJ Publishing Group Ltd ("BMJ") its licensees and where the relevant Journal is co-owned by BMJ to the co-owners of the Journal, to publish the Work in this journal and any other BMJ products and to exploit all rights, as set out in our licence.

The Submitting Author accepts and understands that any supply made under these terms is made by BMJ to the Submitting Author unless you are acting as an employee on behalf of your employer or a postgraduate student of an affiliated institution which is paying any applicable article publishing charge ("APC") for Open Access articles. Where the Submitting Author wishes to make the Work available on an Open Access basis (and intends to pay the relevant APC), the terms of reuse of such Open Access shall be governed by a Creative Commons licence – details of these licences and which Creative Commons licence will apply to this Work are set out in our licence referred to above.

Other than as permitted in any relevant BMJ Author's Self Archiving Policies, I confirm this Work has not been accepted for publication elsewhere, is not being considered for publication elsewhere and does not duplicate material already published. I confirm all authors consent to publication of this Work and authorise the granting of this licence.

For peer review only

BMJ OPEN

TITLE PAGE

Title: Randomised controlled trial of a behaviour change physiotherapy intervention to increase physical activity following hip and knee replacement: the PEP-TALK trial

Authors: Smith TO,[1,2] Parsons SR,[1] Ooms AG,[1,3] Dutton SJ,[1,3] Fordham B,[1] Garrett A,[1] Hing CB,[4] Lamb SE[1,5] on behalf of the PEP-TALK Trial Collaborators.

Affiliations

1. Nuffield Department of Rheumatology, Orthopaedics and Musculoskeletal Sciences, University of Oxford, Botnar Research Centre, Windmill Road, Oxford, UK
2. Faculty of Medicine and Health Sciences, University of East Anglia, Norwich, UK
3. Oxford Clinical Trials Research Unit (OCTRU), Centre for Statistics in Medicine (CSM), University of Oxford, Windmill Road, Oxford, UK
4. Department of Trauma and Orthopaedics, St George's Hospital, St George's University Hospitals NHS Foundation Trust, Tooting, London, UK
5. College of Medicine and Health Sciences, University of Exeter, Exeter, UK

Corresponding Author: Dr Toby Smith, School of Health Sciences, Queen's Building, University of East Anglia, Norwich, UK. Email: toby.smith@uea.ac.uk

Word Count (currently): Abstract: 299 (limit 300); Main Manuscript: 4705

For peer review only

ABSTRACT

OBJECTIVE: To test the effectiveness of a behaviour change physiotherapy intervention to increase physical activity compared with usual rehabilitation after Total Hip Replacement (THR) or Total Knee Replacement (TKR).

DESIGN: Multicentre, pragmatic, two-arm, open, randomised controlled, superiority trial

SETTING National Health Service providers in nine English hospitals.

PARTICIPANTS: 224 individuals aged ≥18 years, undergoing a primary THR or TKR deemed "moderately inactive" or "inactive".

INTERVENTION: Participants received either six, 30-minute, weekly, group-based exercise sessions (usual care), or the same six-weekly, group-based, exercise sessions each preceded by a 30-minute cognitive behaviour discussion group aimed at challenging barriers to physical inactivity following surgery (experimental).

RANDOMISATION & BLINDING: Initial 75 participants were randomised 1:1 before changing the allocation ratio to 2:1 (experimental:usual care). Allocation was based on minimisation, stratifying on comorbidities, operation type and hospital. There was no blinding.

MAIN OUTCOME MEASURES: Primary: UCLA Activity Score at 12 months. Secondary: six and 12 month assessed function, pain, self-efficacy, kinesiophobia, psychological distress and quality of life.

RESULTS: Of the 1254 participants assessed for eligibility, 224 were included (139 experimental:85 usual care). Mean age was 68.4 years (standard deviation: 8.7), 63% were female, 52% underwent TKR. There was no between-group difference in UCLA score (mean difference: -0.03 (95% CI: -0.52 to 0.45, p=0.89)). There were no differences observed in any of the secondary outcomes at six or 12 months. There were no important adverse events in either group. The COVID-19 pandemic contributed to the reduced intended sample size (target 260) and reduced intervention compliance.

CONCLUSIONS: There is no evidence to suggest attending usual care physiotherapy sessions plus a group-based behaviour change intervention differs to attending usual care physiotherapy alone. As the trial could not reach its intended sample size, nor a proportion of participants receive their intended rehabilitation, this should be interpreted with caution.

TRIAL REGISTRATION: ISRCTN29770908

Keywords: arthroplasty; osteoarthritis; rehabilitation; physical activity; exercise; cognitive behavioural

STRENGTHS AND LIMITATIONS OF THIS STUDY

- The multicentre recruitment approach enhanced external validity across population characteristics in England.

For peer review only

- Functional, behavioural and psychological outcomes were collected to ensure a global participant assessment.
- It was challenging to ensure there were acceptable numbers of people in the group-based intervention.
- All 12-month follow-up data were collected during the COVID-19 pandemic, potentially impacting on typical recovery and psychological outcomes.
- The COVID-19 pandemic meant we were unable to reach our anticipated sample size or deliver the intervention as planned.

INTRODUCTION

For peer review only

Total Hip Replacement (THR) and Total Knee Replacement (TKR) are two highly successful orthopaedic procedures which reduce pain for people with osteoarthritis.[1,2] Over 200,000 THRs and TKRs were performed in the United Kingdom (UK) in 2019 pre-pandemic.[1] Approximately 90% of patients are typically satisfied following THR and TKR,[2] with significant improvements in pain and physical function after three to 12 months.[2,3]

Historically, it has been assumed that people become more active following THR or TKR through the amelioration of joint pain.[4] However, current literature suggests physical activity, at best, remains the same from pre- to post-operatively, and in some instances declines.[4,5]

People following THR and TKR have reported a number of challenges which make engaging in physical activity difficult, most notably psychosocial barriers and fear avoidance beliefs.[6] Such barriers include receiving insufficient and inconsistent information on being more physically active, fear of damaging joint replacements and causing pain, and not being able to goal-set or problem-solve physical activities within individual's lifestyles.[6] Whilst previous international guidance has acknowledged the importance of physical activity on health and wellbeing, people following THR and TKR have reported difficulty in being active.[6] There is limited support or guidance currently offered on how to overcome these problems post-operatively.[6]

Not being physically active after joint replacement can have a major negative impact on a person's health and a burden on the National Health Service (NHS). Medical co-morbidities are common in this population. These include hypertension (56%),[7] cardiovascular disease (20%),[8] diabetes (16%)[8] and multi-joint pain (57%).[7] Approximately 27% of people who undergo joint replacement have three or four comorbidities.[8] Medical comorbidities have a significant negative impact on both health-related quality of life (HRQoL) and result in a societal burden.[9,10] Participating in regular physical activity can decrease the risk of cardiovascular disease by 52%,[11] diabetes by 65%,[12] and some cancers by 40%.[13] It is associated with a reduction in all-cause mortality by 33% and cardiovascular mortality by 35%.[14]

Current rehabilitation following THR and TKR in the UK, as advocated by the National Institute for Health and Care Excellence (NICE), centres around regaining joint movement, strength and gait re-education.[15] There is currently no evidence informing patients or healthcare professionals on how to increase physical activity specifically following THR and TKR. Following joint replacement, people have specific psychological needs and challenges which differ to the non-joint replacement population.[6] Therefore, a specific intervention tailored to this population's health beliefs, including

For peer review only - http://bmjopen.bmj.com/site/about/guidelines.xhtml

fear avoidance regarding implant survival, dislocation and increased knowledge on the impact of physical inactivity on other comorbidities, is required. Previous research has demonstrated that behaviour-change interventions can effectively increase physical activity across the lifespan.[16-20] Given this, it was hypothesised that such an intervention could be beneficial for this population. Accordingly, the purpose of this trial was to answer the research question “following a primary THR or TKR, does a group exercise and behaviour-change intervention targeted to increase physical activity participation increase HRQoL and clinical outcomes over the initial 12 post-operative months compared to group exercise alone?”

METHODS

Study design

A full protocol has been published previously.[21]

This was a two-arm, open, pragmatic, parallel, multi-centre, randomised controlled superiority trial. The study flow chart is presented as **Figure 1**. Participants were recruited from eight UK NHS hospital trusts by the clinical team once they had been listed for THR or TKR. Interventions were delivered in physiotherapy departments within these NHS facilities.

We recruited adults who were due to undergo primary unilateral THR or TKR where the indication for surgery was degenerative joint pathology (not trauma). Potential participants were classified as ‘moderately inactive’ or ‘inactive’ using the General Practice Physical Activity Questionnaire (GPPAQ)[22] and have a Charlson Comorbidity Index (CCI) of ≥1 point.[23,24] We excluded people who were cognitively impaired, defined as an Abbreviated Mental Test Score (AMTS)[25] of <8; whose usual place of residence was a care home; were unable to read and/or comprehend English; and had no access to a working telephone.

Study treatments

Usual NHS surgical and in-patient care was received by both control and intervention groups. On hospital discharge, all participants attended six-weekly, 30-minute, group-based exercise classes within each hospital trust’s physiotherapy department. These groups commenced within four weeks post-operation. The principles regarding prescription of group exercises to increase range of motion, strength and gait pattern, were consistent. Whilst the rehabilitation of THR and TKR focuses on overall lower limb function, all participants following a THR focused on hip exercises, whereas those following a TKR focused on knee exercises. One physiotherapist (with or without a second physiotherapist or therapy assistant) ran each session.

The programme and rationale for the experimental intervention are presented in detail in **Supplementary File 1**. In brief, the intervention was grounded in the Social Cognitive Theory (SCT)[26] based on the theory that behaviour (physical activity level) is influenced by bi-directional relationships with personal factors (cognitive, emotional and physical) and environment. In this process, the cognitive behavioural approach in the PEP-TALK intervention, used techniques to identify and target unhelpful thoughts and behaviours in order to produce adaptive thoughts, behaviours, emotions and physiological responses. Previous systematic reviews examining barriers and facilitators for older adults to increase physical activity have identified specific beliefs which could reduce an individual’s general self-efficacy.[4,6,27,28] These include: stigma, body image[28] and ageing stereotypes.[27] Unhelpful beliefs can be identified and explored using cognitive behavioural techniques to increase

For peer review only

self-efficacy. The evidence also identified tools to increase general self-efficacy which include the credibility of instructors and the information/physical activity tasks they provide.[27-29] The PEP-TALK intervention was designed to address these, exploring known barriers and facilitators to physical activity after joint replacement,[6] to promote increased participation in activity post-operatively.

In practice, participants randomised to the experimental group received the same six-weekly, group-based, 30-minute, exercise session as the usual care group. The only difference between the two groups was the addition of a 30-minute, group-based, behaviour change intervention prior to the routine 30 minutes of exercise, and three follow-up telephone calls two, four and six weeks after the last group-based session. In the group-based sessions, participants were facilitated (as a group) to develop skills to overcome challenges to physical activity behaviour, supplemented through a workbook. This encouraged reflective activities such as recording physical, emotional and cognitive barriers and facilitators to physical activity. One physiotherapist (with or without a second physiotherapist or therapy assistant) ran each session. During the follow-up telephone calls, participant's goals were reviewed, any barriers to the completion of these goals were identified, and the physiotherapist reviewed any 'unhelpful' and 'helpful' thoughts or feelings towards physical activity which may have arisen since the last consultation, and closed with the development of longer-term physical activity goal-setting. A treatment log was completed by physiotherapists to record the components of what was discussed across the group in each session and each telephone call.

Each member delivering the experimental intervention attended a one-day training session which taught the components and format of the intervention. To ensure compliance with the treatment protocol, the PEP-TALK team made regular visits for quality assurance.

Data collection

At the time of enrolment, site healthcare professionals checked eligibility and recorded demographic characteristics. Baseline scores for outcome questionnaires were obtained before randomisation. Data collected at baseline included: gender, age, height and weight, CCI, self-reported presence and location of multi-site joint pain, co-morbidities determined from the medical notes, AMTS, employment status and occupation (when appropriate).

Participants were followed-up at six and 12 months after randomisation.

The primary outcome was the University of California Los Angeles (UCLA) Activity Score[30] (scored 0 to 10; higher scores indicate greater physical activity) at 12 months. This was selected as it is a reliable and valid self-reported tool to assess physical activity[31] and has been previously used for this means in orthopaedic trials.[32] Secondary outcomes at six and 12 months after randomisation were measured using the Lower Extremity Functional Scale (LEFS)[33] (scored 0 to 80, higher scores indicating less functional disability), Oxford Hip Score (OHS)[34] or Oxford Knee Score (OKS)[35] (scored 0 to 48, higher scores indicating less disease-specific function), Numerical Rating Scale (NRS) for pain (scored 0 to 10, higher scores indicating greater pain perception), the Generalized Self-Efficacy Scale (GSES)[36] (scored 10 to 40, higher scores indicating greater self-efficacy), the Tampa Scale for Kinesiophobia[37] (scored 17 to 68, higher scores indicating greater fear of motion), the Hospital Anxiety and Depression Scale (HADS)[38] (scored 0 to 21, higher scores indicating greater anxiety and depression), and the EQ-5D-5L[39] (scored 0 to 1, higher scores indicating greater HRQoL). Participants provided a retrospective assessment of complications at each six-month follow-up period. Health resource utilisation data were collected but is not presented in this paper.

For each participant in the experimental intervention arm, the number of trial exercise sessions attended and group size of each session was recorded. The number of telephone contacts made after

the end of the sessions and adherence with intervention protocols was also collected. There were no changes to the outcomes during the trial.

Randomisation and masking

Random allocation was 1:1 originally. Randomisation was performed using a centralised computer randomisation program provided by the Oxford Clinical Trials Research Unit (OCTRU). Research nurses and physiotherapists at recruiting centres enrolled participants and then assigned participants by accessing the online OCTRU randomisation program, thereby adopting a concealed allocation approach. Randomisation was undertaken using a minimisation algorithm, stratified by: hospital site; type of joint replacement (THR or TKR); CCI of one to three versus ≥4.[23,24] It had a probabilistic element introduced to ensure unpredictability of treatment assignment.

The experimental intervention was designed to have three or more people per group.[21] Early sites found it difficult to consistently reach this level of participant numbers with the original 1:1 randomisation allocation. Accordingly, after 75 randomisations, we modified the randomisation ratio to 2:1 in favour of the experimental intervention. This ensured that a greater number of people are allocated to the experimental intervention. The sample size was increased to 260 to account for this change.

Masking participants or the teams providing interventions was not possible.

Sample size

The trial was powered on the single primary outcome of UCLA at 12 months. Originally, 250 participants (125 per arm) were required to detect a standardised effect size of 0.4 with 80% power and 5% (two-sided) significance, and allowing for 20% loss to follow-up. These calculations were based on the primary outcome, UCLA Activity Score at 12 months, assuming a baseline standard deviation of 2.5 and a between-group difference of one.[32] The minimally clinically important difference (MCID) was reported as a within-person difference of 0.92 points.[32]

The target sample size was increased to 260 to account for the change in randomisation ratio.[21]

Results from the secondary outcomes provide supporting evidence for the results from the primary outcome analysis and are not powered separately. No allowance for multiple testing was included as a single primary outcome was considered.

Statistical methods

There was no planned interim analyses or pre-defined stopping rules. Full analysis details are in the published statistical analysis plan.[40]

The primary outcome measure, UCLA at 12 months, was modelled using a linear mixed effects model adjusting for person within centre random effects, CCI, type or operation (TKR or THR), time (six and 12 months) and baseline UCLA score as fixed effects using the intention-to-treat population (participants analysed as randomised). A treatment by time point interaction was included to allow time specific treatment effects to be calculated. This approach makes use of all available data at each time point. The secondary outcomes (LEFS, OKS, OHS, HADS, NRS for pain, GSES, Tampa, EQ-5D-5L Index and EQ-VAS) were analysed using a similar modelling approach. The number of participants with one or more complications were analysed using logistic regression, adjusting for minimisation factors

For peer review only

and treatment. Total number of complications were analysed using Poisson Regression adjusting for the same factors.

Supporting analyses to the primary outcome included an area under the curve (AUC) analysis and complier average causal effect (CACE) analyses for all three pre-defined levels of compliance (Strict Compliance, Compliance, Attendance).[40] Full definitions of the three compliance levels are given in Supplementary **File 2.** The AUC analysis provided additional information on the trajectory of function recovery of these participants. The CACE analysis answered the question, for those participants who received the intervention as planned, did it improve function over usual care alone? The AUC analysis was performed using the same model as used for the primary analysis except including baseline UCLA Activity Score in the "time" fixed effect allowing time point specific treatment effects to be calculated for baseline, six and 12 months. The CACE analysis has been performed through 10000 bootstrapped samples. Adjusted linear regression was used for the 12-month UCLA Activity Score; adjusting for randomised treatment, baseline UCLA Activity Score, recruiting site, CCI (continuous), and joint replacement was used to obtain ITT estimates. The pathway from treatment allocation to compliance (rate of potential compliers in the usual care group) was also estimated using adjusted linear regression: compliance indicators was analysed adjusting for the same variables. CACE estimates were obtained by taking the ratio of the ITT estimate and potential complier rate. Standard errors, confidence intervals and p-values were calculated using the bootstrapped samples.

Other analyses examining the missing data assumptions, the per-protocol population, using a reduced model, treatment effects within pre-defined clinical subgroups and exploratory descriptive statistics for selected secondary outcomes by COVID-19.

Study monitoring

A Trial Steering Committee (TSC) and Data Safety Monitoring Committee (DSMC) were appointed to independently review data on safety, protocol adherence and trial recruitment.

Patient and public involvement

Patient involvement began during protocol development and continued throughout the trial. A patient-member (not enrolled in the trial) attended TSC meetings. The same patient-member was a co-investigator. He provided insights into the trial conduct, particularly on data collection processes and helped interpret the findings to inform the trial's dissemination phase.

Trial participants who expressed an interest in receiving information on the trial findings were provided with this.

RESULTS

Recruitment and participant flow

Recruitment occurred between 12 April 2019 to 27 March 2020. The CONSORT[41] flow chart is presented as **Figure 1.** In total, 230 participants were randomised. Six were randomised in error, resulting in an analysable population of 224 participants (85 usual care; 139 experimental).

For peer review

Due to the COVID-19 pandemic, 47 participants that had consented to take part in the study could not be randomised and the trial was stopped 30 participants short of its planned sample size. All elective THRs and TKRs were cancelled as part of the UK national COVID-19 lockdown (23rd March 2020). Group-based physiotherapy classes within the participating hospital outpatient settings (a mechanism this trial relied on for both treatment groups) were also halted. Consequently, it was not feasible to continue the trial for the final 30 planned participants.

Retention

The retention of participants is presented in **Figure 1**. There were 37 withdrawals (13 usual care; 24 experimental). **Supplementary Table 1** gives a summary of type of withdrawals by level of withdrawal and treatment group. The return of primary outcome data is presented in **Supplementary Table 2**. This illustrates that for the primary, ITT, analysis of the UCLA Activity Score there were 223 (99.6%) participants to supply a UCLA Activity Score at baseline (85 usual care; 138 experimental), 186 (83.0%) responses at six months (69 usual care; 117 experimental) and 181 (80.8%) responses at 12 months (70 usual care; 111 experimental).

Participant characteristics

Baseline characteristics are presented by randomised treatment group in **Table 1**. The mean participant age was 68.4 years (standard deviation (SD): 8.7), 62.9% were female with 52.2% undergoing TKR. Seventy-four percent of the cohort had a CCI of one to three (mean 2.9 (SD: 1.3)). Mean BMI was 30.9kg/m^2 (SD: 5.7). The mean duration of symptoms prior to surgery was 46.9 months (SD: 50.9) with 73.2% presenting with an American Society of Anesthesiology (ASA) grade of two at surgery. As **Table 1** demonstrates, the two groups were comparable with the experimental group presenting with a slightly higher proportion of females (64.7% vs. 60.0%), longer duration of symptoms (mean: 48.8 months vs. 43.8 months) and fewer inactive participants (79.1% vs. 83.5%) compared to the usual care group.

Table 1: Baseline characteristics by randomised group

	**Usual (n=85)**	**Experimental (n=139)**	**Total (n=224)**
Age, years	n=85, 68.5 (8.8)	n=139, 68.3 (8.6)	n=224, 68.4 (8.7)
UCLA Activity Score, 1-10	n=85, 3.6 (1.5)	n=138, 3.6 (1.6)	n=223, 3.6 (1.5)
**Joint Replacement**			
Hip replacement	40 (47.1)	67 (48.2)	107 (47.8)
Knee replacement	45 (52.9)	72 (51.8)	117 (52.2)
**CCI, Dichotomised**			
1-3	64 (75.3)	102 (73.4)	166 (74.1)
4+	21 (24.7)	37 (26.6)	58 (25.9)
CCI, Continuous	n=85, 2.8 (1.3)	n=139, 3.0 (1.3)	n=224, 2.9 (1.3)
**Sex**			
Female	51 (60.0)	90 (64.7)	141 (62.9)
Male	34 (40.0)	49 (35.3)	83 (37.1)
**BMI, Categories**			
Healthy Weight	15 (17.6)	25 (18.0)	40 (17.9)
Overweight	22 (25.9)	45 (32.4)	67 (29.9)
Obese	42 (49.4)	60 (43.2)	102 (45.5)

	Usual (n=85)	Experimental (n=139)	Total (n=224)
Morbidly Obese	6 (7.1)	9 (6.5)	15 (6.7)
BMI, kg/m2	n=85, 31.1 (5.9)	n=139, 30.7 (5.6)	n=224, 30.9 (5.7)
**Joint Pain in the Past 7 Days**			
Yes	85 (100.0)	138 (99.3)	223 (99.6)
No	0 (0.0)	1 (0.7)	1 (0.4)
**GPPAQ Level**			
Active	0 (0.0)	0 (0.0)	0 (0.0)
Moderately Active	2 (2.4)	1 (0.7)	3 (1.3)
Moderately Inactive	12 (14.1)	28 (20.1)	40 (17.9)
Inactive	71 (83.5)	110 (79.1)	181 (80.8)
AMTS	n=85, 9.6 (0.6)	n=139, 9.6 (0.6)	n=224, 9.6 (0.6)
EQ-5D-5L Score	n=85, 0.4 (0.2)	n=139, 0.4 (0.3)	n=224, 0.4 (0.2)
EQ-VAS, 0-100	n=85, 61.3 (20.0)	n=139, 60.6 (23.6)	n=224, 60.9 (22.2)
Numeric Pain, 0-10	n=85, 6.9 (1.9)	n=139, 7.2 (1.8)	n=224, 7.1 (1.9)
Symptom Duration, Months	n=85, 43.8 (48.8)	n=138, 48.8 (52.2)	n=223, 46.9 (50.9)
**ASA Classification**			
1	4 (4.7)	12 (8.6)	16 (7.1)
2	61 (71.8)	103 (74.1)	164 (73.2)
3	20 (23.5)	22 (15.8)	42 (18.8)
4	0 (0.0)	2 (1.4)	2 (0.9)

Data are mean (SD-standard deviation) or n (%). +Stratification factor used in randomisation. UCLA=University of California, Los Angeles, CCI=Charlson Comorbidity Index, BMI=Body Mass Index, GPPAQ=General Practice Physical Activity Questionnaire, AMTS=Abbreviated Mental Test Score, EQ-5D-5L=Health-related quality of life assessed by EuroQol 5-level EQ-5D, EQ-VAS=EuroQol Visual Analogue Scale; ASA=American Society of Anesthesiologists.

<u>*Main analyses*</u>

The results of the analysis for the primary outcome measure are presented in **Table 2** and **Figure 2**. There was no evidence to support rejecting the null hypothesis that there was no difference between attending group-based exercise plus a group-based behaviour change intervention and attending group-based exercise alone on the UCLA Activity Score at 12 months post-randomisation, at the 5% significance level (mean difference: -0.03; 95% CI: -0.52 to 0.45; p=0.89). However, as the trial could not reach its intended final sample size due to the COVID-19 pandemic, this result should be interpreted with caution. The interpretation of the results did not change on per-protocol analysis or reduced model analysis (**Supplementary Table 3; Supplementary Table 4**).

Table 2: UCLA Activity Score (primary outcome) results

Time Point	Usual	Experimental	Mean Difference		p-value
	n, Mean (SD)	n, Mean (SD)	Unadjusted	Adjusted (95% CI)	
Baseline	n=85, 3.62 (1.52)	n=138, 3.57 (1.57)	-0.06	-	-
6 Months	n=69, 4.77 (1.52)	n=117, 4.97 (1.68)	0.20	0.27 (-0.21,0.76)	0.27
**12 Months (Primary Outcome)**	**n=70, 4.87 (1.61)**	**n=111, 4.84 (1.91)**	**-0.03**	**-0.03 (-0.52,0.45)**	**0.89**
Area under the curve over 12 months	4.81 (0.29)	4.89 (0.28)	-	0.09 (-0.47,0.64)	0.88
CACE: Strict Compliance	-	n=46	-	-0.24 (-1.45,0.96)	0.69

Time Point	Usual	Experimental	Mean Difference		p-value
	n, Mean (SD)	n, Mean (SD)	Unadjusted	Adjusted (95% CI)	
CACE: Compliance	-	n=58	-	-0.20 (-1.19,0.79)	0.69
CACE: Attendance	-	n=81	-	-0.16 (-0.90,0.59)	0.68

N - number of participants; SD – standard deviation; CACE – complier average causal effect.
For the AUC analysis, the standard deviations presented are the standard errors for these estimates calculated using the delta method. CACE analysis based on 10000 bootstrapped samples.

Three Complier Average Causal Effect (CACE) estimations were performed on the 12-month UCLA Activity Score, one for each definition of compliance (Strict Compliance, Compliance and Attendance). **Table 2** presents the CACE estimates for the three levels of compliance. There was no difference in outcome based on these analyses and all effect estimates were within the MCID of 0.92.[34]

The results of all continuous secondary outcomes are presented in **Table 3**. They demonstrate no significant between-group differences for any of the continuous secondary outcomes at any time point. A general pattern of improvement from baseline to six months then levelling off at 12 months with no significant between-group differences observable, was seen throughout.

A total of 141 complications were reported from 75 participants, 50 (35.5%) in the usual care group and 91 (64.5%) in the experimental group (**Table 4**; **Supplementary Figure 1**). It should be noted that 62.1% of participants were randomised to the experimental group so this apparent difference is expected if complication rate was the same across both groups. The most common complications were increased pain either in the operated joint or in other joints, wound infections, medical complications and stiffness in the operated joint. Most complications (65.2%) were reported in the first six months post-randomisation. There was no difference in the number of people who had a complication (28 vs. 47; odd ratio (OR): 1.03; 95% CI: 0.56 to 1.89) or total numbers of complications (50 vs. 91; OR: 1.10; 95% CI: 0.77 to 1.56) between the usual care and experimental group respectively. There was one adverse event (fall, usual care) and three serious adverse events (two experimental (cardiac failure, pneumonia), one usual care (suspected deep vein thrombosis)).

Table 3: Continuous secondary outcome results

Time Point	Usual	Experimental	Mean Difference		p-value
	n, Mean (SD)	n, Mean (SD)	Unadjusted	Adjusted (95% CI)	
**Lower Extremity Functional Scale**					
Baseline	n=82, 23.72 (13.11)	n=130, 24.50 (14.07)	0.78	-	-
6 Months	n=45, 45.40 (19.76)	n=80, 51.44 (17.70)	6.04	2.60 (-3.29,8.50)	0.39
12 Months	n=51, 47.86 (18.97)	n=80, 50.67 (21.40)	2.81	1.26 (-4.61,7.13)	0.67
**Oxford Hip Score**					
Baseline	n=40, 16.05 (6.36)	n=67, 16.78 (7.99)	0.73	-	-
6 Months	n=28, 34.84 (11.73)	n=50, 39.68 (8.93)	4.84	3.86 (-0.92,8.64)	0.11
12 Months	n=27, 36.90 (12.48)	n=48, 39.42 (10.46)	2.52	2.37 (-2.53,7.27)	0.34
**Oxford Knee Score**					
Baseline	n=45, 18.67 (8.51)	n=72, 17.46 (6.99)	-1.21	-	-
6 Months	n=33, 35.20 (7.62)	n=51, 33.45 (9.38)	-1.75	-1.74 (-5.03,1.54)	0.30
12 Months	n=35, 34.90 (8.46)	n=55, 33.54 (9.84)	-1.36	-1.43 (-4.72,1.86)	0.39

Time Point	Usual	Experimental	Mean Difference		p-value
	n, Mean (SD)	n, Mean (SD)	Unadjusted	Adjusted (95% CI)	
**Numerical Rating Scale for Pain**					
Baseline	n=85, 6.87 (1.94)	n=139, 7.23 (1.79)	0.36	-	-
6 Months	n=61, 3.34 (2.59)	n=101, 3.54 (2.74)	0.20	0.19 (-0.64,1.02)	0.66
12 Months	n=61, 4.08 (2.87)	n=102, 3.33 (2.85)	-0.75	-0.75 (-1.59,0.09)	0.08
**Generalized Self-Efficacy Scale**					
Baseline	n=84, 31.31 (5.49)	n=138, 31.67 (5.39)	0.36	-	-
6 Months	n=58, 31.88 (5.18)	n=98, 33.03 (5.30)	1.15	1.15 (-0.30,2.61)	0.12
12 Months	n=61, 32.16 (5.55)	n=101, 32.20 (6.72)	0.03	0.33 (-1.13,1.78)	0.66
**Tampa Scale for Kinesiophobia**					
Baseline	n=85, 40.04 (7.44)	n=136, 39.77 (7.75)	-0.26	-	-
6 Months	n=56, 35.77 (7.74)	n=91, 34.77 (7.29)	-1.00	-0.39 (-2.40,1.61)	0.70
12 Months	n=57, 36.56 (6.91)	n=90, 35.06 (8.27)	-1.51	-0.77 (-2.79,1.24)	0.45
**Hospital Anxiety and Depression Scale (Overall)**					
Baseline	n=85, 11.85 (6.16)	n=138, 12.50 (7.07)	0.65	-	-
6 Months	n=59, 8.97 (6.52)	n=97, 8.81 (6.36)	-0.15	-1.18 (-2.73,0.37)	0.14
12 Months	n=62, 9.02 (6.61)	n=98, 9.70 (6.99)	0.69	0.52 (-1.03,2.06)	0.51
**Hospital Anxiety and Depression Scale (Anxiety)**					
Baseline	n=85, 5.89 (3.78)	n=138, 6.63 (4.07)	0.74	-	-
6 Months	n=60, 4.95 (4.01)	n=98, 4.95 (3.57)	0.00	-0.71 (-1.67,0.25)	0.15
12 Months	n=62, 4.76 (3.73)	n=99, 5.46 (3.84)	0.71	0.36 (-0.60,1.31)	0.46
**Hospital Anxiety and Depression Scale (Depression)**					
Baseline	n=85, 5.95 (3.16)	n=139, 5.89 (3.81)	-0.06	-	-
6 Months	n=61, 4.03 (3.27)	n=99, 3.90 (3.51)	-0.13	-0.25 (-1.13,0.63)	0.58
12 Months	n=62, 4.26 (3.47)	n=101, 4.30 (4.02)	0.04	0.24 (-0.65,1.12)	0.60
**EQ-5D-5L Index**					
Baseline	n=85, 0.40 (0.22)	n=139, 0.39 (0.27)	-0.01	-	-
6 Months	n=68, 0.66 (0.23)	n=117, 0.69 (0.25)	0.03	0.03 (-0.03,0.10)	0.31
12 Months	n=70, 0.67 (0.24)	n=113, 0.67 (0.29)	0.00	0.00 (-0.06,0.07)	0.93
**EQ-VAS**					
Baseline	n=85, 61.33 (20.01)	n=139, 60.58 (23.56)	-0.75	-	-
6 Months	n=68, 70.93 (18.67)	n=117, 73.86 (20.02)	2.94	2.84 (-2.31,7.99)	0.28
12 Months	n=69, 72.51 (17.90)	n=110, 72.94 (19.98)	0.43	1.47 (-3.73,6.68)	0.58

Table 4: Complication results

	Usual	Experimental	Odds Ratio	p-value
	N (%)	N (%)	(95% CI)	
Number of participants who had a complication	28 (32.94)	47 (33.81)	1.03 (0.56,1.89)	0.94
Total complications	50 (58.82)	91 (65.47)	1.10 (0.77,1.56)	0.61

CI – confidence intervals

Analysis by compliance

Treatment compliance is summarised in **Supplementary Figure 2**. Compliance is reported by categories as defined in the analysis plan.[40] In total, 489 experimental intervention or physiotherapy exercises sessions were held. The sessions ran from 08 May 2019 to 18 March 2020. 162 were

experimental sessions and 327 were exercise alone sessions (161 usual care; 166 experimental). There was one experimental class that was not accompanied by a physiotherapy class.

A major component of the definition of compliance for the experimental group was the group class sizes. The median class size for the intervention classes was two with a range of one to 14. **Supplementary Figure 3** is a plot of the group sizes for all intervention sessions. Any class with three or more participants was considered a “compliant” class. In total, 75 (46.3%) of the 162 intervention sessions had three or more participants. To address the issue of compliance, the randomisation procedure was changed from 1:1 to 2:1. **Supplementary Figure 4** is a breakdown of treatment compliance by participants randomised using either a 1:1 or 2:1 randomisation ratio. In both groups, the number of participants who were non-compliant rose considerably and the number of strict compliers fell after the change from 1:1 to 2:1 randomisation. A confounder to this result is that participants whose intervention was disrupted by COVID-19 were all randomised using a 2:1 ratio. The large increase in non-compliance in that population is seen in **Supplementary Figure 4**.

Impact of COVID-19 on trial findings

The level of disruption to the intervention delivery caused by the COVID-19 pandemic was high. There was a high level of non-compliance, particularly in the experimental group. This apparent between-group difference in non-compliance was because the pre-defined definitions of compliance were stricter in the experimental than the usual care group. To be an “Attender” in the experimental group, one needed to attend four out of six group intervention sessions, to achieve the same level of compliance in the usual care group, only one session was required to be attended. In the usual care group, 66 (77.6%) attended at least one physiotherapy session, a similar proportion, 111 (80%), attended at least one physiotherapy session in the experimental group. Due to the added therapy the experimental group received, the definition for compliance had to be stricter but both groups had a similar proportion who attended at least one session.

The final months of the trial, before all group-based physiotherapy classes within the hospital outpatient setting were halted due to the COVID-19 pandemic, yielded the highest group sizes. **Supplementary Figure 4** summarises the compliance to the experimental group by pre-COVID-19 compared to COVID-19 to estimate the impact of the pandemic on compliance. This is plotted by time in **Supplementary File 3**. Based on this, a large proportion of participants who could not be randomised due to the trial closure would have ended up falling into either the “Compliant” or “Strict Compliant” groups.

Additional analyses

The missing data analysis suggests that the missing at random assumption made in the primary analysis is appropriate (**Supplementary Figure 5**). The per-protocol and reduced model results support the main findings from the trial and there was no evidence of any difference in the exploratory subgroup analysis. The exploratory descriptive statistics by COVID-19 status may suggest participants in the COVID-19 group had poorer psychological outcomes (**Supplementary Table 5**). The results are presented in full in **Supplemental Figure 6**.

DISCUSSION

The findings suggest that following THR or TKR, there is no difference between the addition of a group-based exercise and behaviour change intervention in physical activity and other clinical outcomes

For peer review only

during the first post-operative year compared to attending group-based exercise alone. However, the COVID-19 pandemic significantly impacted on this trial whereby the intended sample size was not achieved, and a considerable proportion of participants were unable to receive their intended post-operative rehabilitation. Accordingly, these findings should be interpreted with caution.

The rationale for undertaking this study was the uncertainty over how to increase physical activity following THR and TKR. Whilst several studies have been published over the intervening period acknowledging that physical activity remains low following joint replacement,[42-44] there continues to be uncertainty over how to overcome this. Studies in other populations, most notably older adults, individuals with chronic respiratory disorders and those with chronic rheumatological diseases have provided promise that a behaviour change intervention may improve physical activity.[17-20] As previously acknowledged, the specific challenges which individuals face in relation to fear avoidance, beliefs about implant failure, multi-joint pain and other comorbidities[6] may account for why this behaviour change intervention did not demonstrate similar changes. However this trial specifically relates to the effectiveness of a behaviour intervention targeted to the behaviour change construct of self-efficacy in the joint replacement population. There may remain value for future research exploring the effectiveness of other behaviour change constructs, to increase physical activity after these orthopaedic procedures. Furthermore, the results from this trial have been impacted by the COVID-19 pandemic, principally on intervention delivery and compliance. Given the impact COVID-19 had, there still remains a need to better understand how to increase physical activity following THR or TKR.

Trial participants understood the research objective was to explore the effectiveness of an intervention aimed at increasing physical activity following THR or TKR. However, compliance to the intervention was low throughout the trial. Accordingly, the appetite to increase physical activity remains uncertain. Previous literature has suggested that whilst individuals may be no more physically active after joint replacement,[44,45] clinical outcomes and specifically pain do significantly improve.[46,47] This corresponds with an improvement in HRQoL. Patient satisfaction to outcome and expectations may be met but this is not translated into increased physical activity. Given the wider health benefits which physical activity confers, consideration should be made on how health professionals promote physical activity messages within post-operative recovery programmes so added health gains are maximised. How this is operationalised following this trial's findings, remains unclear.

Whilst the results indicate no superiority to the addition of a behaviour change intervention to usual physiotherapy rehabilitation after TKR or THR, the findings offer important clinical implications. Firstly, the trial indicates that joint replacement and usual physiotherapy rehabilitation can improve clinical outcomes. Previous literature suggests improvements in pain, function and HRQoL[46,47] for people following THR and TKR. However, the trial also indicates both pre- and post-COVID-19 that there were differences in adherence and compliance to both usual and experimental physiotherapy interventions. Whilst previous literature has highlighted geographical and service-provision differences in rehabilitation after joint replacement,[48,49] there has been limited evidence to indicate variability in adherence to rehabilitation. This may reflect variation in rehabilitation need. Whilst some patients may need substantial levels of physiotherapy following joint replacement to promote physical function, activity and improvements in HRQoL, these may not be homogeneous within the population.[50] Stratification on rehabilitation need may therefore be warranted. Whilst previous authors have attempted to identify those at most risk of poor outcomes post-operative,[51,52] there remains uncertainty over what physiotherapy intervention is more beneficial for these patients. Further consideration on the optimal rehabilitation programme to promote physical activity for those with the most to gain as opposed to assuming all, as adopted in this trial, may be indicated.

There are several trial strengths and limitations to be considered. A major strength was the pragmatic approach taken to assess effectiveness. The broad eligibility criteria to reflect typical patients who undergo THR and TKR, balanced by the inclusion of only those, who were pre-operatively moderately inactive or inactive, meant the eligibility criteria were constructed to theoretically recruit those who had the most to gain. The multi-site, national recruitment process across NHS health trusts also offered the ability to recruit a diverse cohort in relation to socioeconomic, ethnic and geographical factors. However, a limitation to the design was that several measures which may have characterised such diversity including level of deprivation, educational status, ethnicity and educational background were not collected. This decision was made to offer a more efficient data collection process, not over-burdening participants with extensive demographic data requests. Smith et al[53] previously acknowledged this as a recurrent limitation to musculoskeletal research. Future research should consider the impact of socioeconomic and deprivation factors both on the design of interventions, processes and analysis. A further limitation was the impact of COVID-19. Whilst acknowledged that the trial over-recruited, consenting 277 participants, only 230 were randomised as the pandemic disrupted surgical and rehabilitation delivery. This means the results were underpowered to answer the trial's primary research question. Secondly, 69 individuals who were receiving rehabilitation during this time had their intervention delivery impacted on this change in service provision. Consequently, intervention compliance reduced, impacting on any effect estimate generated from that point onwards. Given this equated to 30% of the cohort, it is proposed this had a significant impact. What is more difficult to estimate is the impact of the COVID-19 social restrictions on outcome. All participants experienced the 2020 social restrictions prior to completing their 12-month questionnaires (first 12-month questionnaire completed 23 March 2020). Whilst previous studies[54,55] indicate that individuals with joint pain substantially reduced their natural physical activity engagement during this time, we did not specifically collect data to ascertain the effects of 'lockdown' on outcomes. The effect of this on 12-month results should therefore be considered.

CONCLUSIONS

The addition of a group-based behaviour change intervention to usual physiotherapy rehabilitation following primary THR and TKR does not offer benefit over usual physiotherapy alone on physical activity and clinical outcomes over the first 12 post-operative months. These findings should be viewed with caution as the COVID-19 pandemic impacted on both the ability of participants to undergo joint replacement and compliance to their rehabilitation. Given the health and social benefits which being active offers older adults, further exploration on methods to increase physical activity for those who are inactive following joint replacement remains important.

For peer review only

DECLARATIONS AND ACKNOWLEDGEMENTS

Authors' Contributors: TS, BF, SJD, SL, AO, CH, AG, SP researched the topic and devised the study. TS, SP, AO, BF, SJD, CH, AG, SL provided the first draft of the manuscript. AO, SD provided statistical analysis and oversight. TS, SP, BF, AO, SJD, CH, AG, SL contributed equally to manuscript preparation. TS acts a guarantor.

Funding Statement: The research is supported by the National Institute for Health Research (NIHR) Research for Patient Benefit grant (PB-PG-1216-20008). The views expressed are those of the author(s) and not necessarily those of the NHS, the NIHR or the Department of Health and Social Care.

Competing Interests Statement: None declared.

Disclaimer: The views expressed are those of the author(s) and not necessarily those of the NHS, the NIHR or the Department of Health and Social Care.

Ethics Approval: Ethical approval was gained from the South Central (Oxford B) Research Ethics Committee (Approval Date: 23Oct2018; Reference Number: 18/SC/0423).

Trial Sponsor: University of Oxford (Clinical Trials and Research Governance Team. Joint Research Office, 1st floor, Boundary Brook House, Churchill Drive, Headington, Oxford OX3 7GB. Email: ctrg@admin.ox.ac.uk. The views expressed are those of the author(s) and not necessarily those of the sponsor.

Provenance and Peer Review: Not commissioned; externally peer reviewed.

PEP-TALK Collaborators: Mr Steve Algar (Banbury, PPI Representative), Dr Zara Hansen (ZH) (University of Oxford), Dr Vicki Barber (Oxford Clinical Trials Research Unit (OCTRU), University of Oxford), Mr Malcolm Hart (OCTRU), University of Oxford), May Ee Png (OCTRU, University of Oxford), Professor Karen Barker (Principal Investigator - Oxford University Hospital NHS Foundation Trust (OUH)**,** Mr Ian Smith (Principal Investigator **-** Lewisham and Greenwich University Hospitals NHS Foundation Trust), Professor Iain McNamara (Principal Investigator (Norfolk and Norwich University Hospitals NHS Foundation Trust (NNUH) & Spire Norwich), Mr Michael Dunn (Principal Investigator - St George's University Hospital NHS Foundation Trust), Mrs Dawn Lockey (Principal Investigator - City Hospitals Sunderland NHS Foundation Trust), Mr Sonny Driver (Principal Investigator – North Middlesex University Hospital NHS Trust), Mrs Jamila Kassam (Barts Health NHS Trust), Mr Peter Penny (Norwich Spire, Norwich), Mrs Celia Woodhouse, Mrs Tracey Potter and Mrs Helena Daniell (NNUH), Mr Alex Herring and Mrs Yan Cunningham (City Hospitals Sunderland NHS Foundation Trust), Irrum Afzal (South West London Elective Orthopaedic Centre), Mr Maninderpal Matharu (Barts Health NHS Trust) and Mrs Tamsin Hughes, Ms Erin Hannink and Mrs Michelle Moynihan (OUH).

Oversight Committee Membership: TSC Members: Professor David Deehan (Newcastle University), Dr Emma Godfrey (Kings College London), Dr Neil Artz (University of the West of England), Mr Steve Algar (PPI representative). **DSMC Members:** Dr Lindsey Smith (University of the West of England & Weston Area Health NHS Trust), Dr Dipesh Mistry (University of Warwick), Mr Paul Baker (South Tees Hospital NHS Foundation Trust)

Patient consent for publication: Not required.

For peer review only

Data Sharing Statement: Access to the de-identified dataset for purposes of research other than this study, would be at the discretion of the Chief Investigator, Dr Toby Smith and OCTRU. Requests for the de-identified dataset generated during the current study should be made to the Chief Investigator, Dr Toby Smith (email: toby.smith@uea.ac.uk) or OCTRU (octrutrialshub@ndorms.ox.ac.uk). Dr Toby Smith and OCTRU will consider requests once the main results from the study have been published up until 31st December 2026.

For peer review only

FIGURE AND TABLE LEGENDS

Figure 1: CONSORT Flow-Chart

Figure 2: UCLA Activity Score boxplots

Table 1: Baseline characteristics by randomised group

Table 2: UCLA Activity Score (primary outcome) results

Table 3: Continuous secondary outcome results

Table 4: Complication results

Supplementary File 1: PEP-TALK programme intervention outline and development

Supplementary File 2: Additional results

Supplementary Table 1: Withdrawals summary

Supplementary Table 2: Questionnaire returns by treatment group

Supplementary Table 3: UCLA Activity Score per-protocol results

Supplementary Table 4: UCLA Activity Score reduced model (no recruiting centre random effect) results

Supplementary Table 5: Descriptive results for selected secondary outcomes by COVID-19 status

Supplementary Figure 1: Complication type by randomised group

Supplementary Figure 2: Overall compliance by (a) raw frequencies and (b) percentage of randomised group

Supplementary Figure 3: Experimental intervention group sizes over time, including change from a randomisation ratio of 1:1 to 2:1

Supplementary Figure 4: Experimental intervention group compliance by COVID-19 group

Supplementary Figure 5: 12 month adjusted mean difference UCLA Activity Score for varying imputed quantiles for missing data

Supplementary Figure 6: Subgroup analyses results

For peer review only

REFERENCES

1. National Joint Registry. 18th Annual Report. Accessed 19.12.2020. Available at: NJR 18th Annual Report 2021.pdf (njrcentre.org.uk)

2. Dailiana ZH, Papakostidou I, Varitimidis S, Liaropoulos L, Zintzaras E, Karachalios T, Michelinakis E, Malizos KN. Patient-reported quality of life after primary major joint arthroplasty: a prospective comparison of hip and knee arthroplasty. *BMC Musculoskelet Disord* 2015;16:366.

3. Papakostidou I, Dailiana ZH, Papapolychroniou T, Liaropoulos L, Zintzaras E, Karachalios TS, Malizos KN. Factors affecting the quality of life after total knee arthroplasties: a prospective study. *BMC Musculoskelet Disord* 2012;13:116.

4. Withers TM, Lister S, Sackley C, Clark A, Smith TO. Is there a difference in physical activity levels in patients before and up to one year after unilateral total hip replacement? A systematic review and meta-analysis. *Clin Rehabil* 2017:316;39-50.

5. Smith TO, Hilton G, Mansfield M, Mann CJV, Sackley CM. Does physical activity change following hip and knee arthroplasty? An analysis of data from the Osteoarthritis Initiative. *Physiotherapy* 2018; 104; 80-90.

6. Smith TO, Latham S, Maskrey V, Blyth A. Patients' perceptions of physical activity before and after joint replacement: a systematic review with meta-ethnographic analysis. *Postgrad Med J 2015;91:483-91.*

7. Inacio MC, Pratt NL, Roughead EE, Graves SE. Comparing co-morbidities in total joint arthroplasty patients using the RxRisk-V, Elixhauser, and Charlson Measures: a cross-sectional evaluation. *BMC Musculoskelet Disord* 2015;16:385.

8. Peter WF, Dekker J, Tilbury C, Tordoir RL, Verdegaal SH, Onstenk R, Bénard MR, Vehmeijer SB, Fiocco M, Vermeulen HM, van der Linden-van der Zwaag HM, Nelissen RG, Vliet Vlieland TP. The association between comorbidities and pain, physical function and quality of life following hip and knee arthroplasty. *Rheumatol Int* 2015;35:1233-41.

9. Smith E, Hoy DG, Cross M, Vos T, Naghavi M, Buchbinder R, Woolf AD, March L. PICO. The global burden of other musculoskeletal disorders: estimates from the Global Burden of Disease 2010 study. *Ann Rheum Dis* 2014;73:1462-9.

10. Sampson UK, Fowkes FG, McDermott MM, Criqui MH, Aboyans V, Norman PE, Forouzanfar MH, Naghavi M, Song Y, Harrell FE Jr, Denenberg JO, Mensah GA, Ezzati M, Murray C. Global and regional burden of death and disability from peripheral artery disease: 21 world regions, 1990 to 2010. *Glob Heart* 2014;9:145-58.e21.

11. Lee CD, Folsom AR, Blair SN. Physical activity and stroke risk: a meta-analysis. *Stroke* 2003;34:2475-81.

12. Laaksonen DE, Lindström J, Lakka TA, Eriksson JG, Niskanen L, Wikström K, Aunola S, Keinänen-Kiukaanniemi S, Laakso M, Valle TT, Ilanne-Parikka P, Louheranta A, Hämäläinen H, Rastas M, Salminen V, Cepaitis Z, Hakumäki M, Kaikkonen H, Härkönen P, Sundvall J, Tuomilehto J, Uusitupa M; Finnish diabetes prevention study. Physical activity in the prevention of type 2 diabetes: the Finnish diabetes prevention study. *Diabetes* 2005;54:158-65.

For peer review only

13. Lee IM. Physical activity and cancer prevention--data from epidemiologic studies. *Med Sci Sports Exerc* 2003;35:1823-7.

14. Kersten RF, Stevens M, van Raay JJ, Bulstra SK, van den Akker-Scheek I. Habitual physical activity after total knee replacement. *Phys Ther* 2012;92:1109-16.

15. National Guideline Centre (UK). Evidence review for information needs: Joint replacement (primary): hip, knee and shoulder: Evidence review A. London: National Institute for Health and Care Excellence (UK); 2020.

16. French DP, Olander EK, Chisholm A, Mc Sharry J. Which behaviour change techniques are most effective at increasing older adults' self-efficacy and physical activity behaviour? A systematic review. *Ann Behav Med* 2014;48:225-34.

17. O'Dwyer T, Monaghan A, Moran J, O'Shea F, Wilson F. Behaviour change intervention increases physical activity, spinal mobility and quality of life in adults with ankylosing spondylitis: a randomised trial. *J Physiother* 2017;63:30-9.

18. Burkow TM, Vognild LK, Johnsen E, Bratvold A, Risberg MJ. Promoting exercise training and physical activity in daily life: a feasibility study of a virtual group intervention for behaviour change in COPD. *BMC Med Inform Decis Mak* 2018;18:136.

19. Boulton E, Hawley-Hague H, French DP, Mellone S, Zacchi A, Clemson L, Vereijken B, Todd C. Implementing behaviour change theory and techniques to increase physical activity and prevent functional decline among adults aged 61-70: The PreventIT project. *Prog Cardiovasc Dis* 2019;62:147-56.

20. Samdal GB, Eide GE, Barth T, Williams G, Meland E. Effective behaviour change techniques for physical activity and healthy eating in overweight and obese adults; systematic review and meta-regression analyses. *Int J Behav Nutr Phys Act* 2017;14:42.

21. Smith TO, Parsons S, Fordham B, Ooms A, Dutton S, Hing C, Barber VS, Png ME, Lamb S; PEP-TALK Trial Collaborators. Behaviour change physiotherapy intervention to increase physical activity following hip and knee replacement (PEP-TALK): study protocol for a pragmatic randomised controlled trial. *BMJ Open* 2020;10:e035014.

22. Department of Health. 2013. General Practice Physical Activity Questionnaire. Accessed on 21.02.2017. Available at: https://www.gov.uk/government/publications/general-practice-physical-activity-questionnaire-gppaq

23. Charlson ME, Pompei P, Ales KL, MacKenzie CR. A new method of classifying prognostic comorbidity in longitudinal studies: development and validation. *J Chronic Dis* 1987;40:373-83.

24. Charlson ME, Charlson RE, Briggs W, Hollenberg J. Can disease management target patients most likely to generate high costs? The impact of comorbidity. *J Gen Intern Med* 2007;22:464-9.

25. Hodkinson HM. Evaluation of a mental test score for assessment of mental impairment in the elderly. *Age Ageing* 1972;1: 233–8.

26. Bandura A. Social foundations of thought and action: A social cognitive theory. Prentice-Hall Inc, 1986.

For peer review only - http://bmjopen.bmj.com/site/about/guidelines.xhtml

27. Arnautovska U, O'Callaghan F, Hamilton K. Applying the integrated behavior change model to understanding physical activity among older adults: a qualitative study. *J Sport Exerc Psychol* 2017;39:43-55.

28. Olanrewaju O, Kelly S, Cowan A, Brayne C, Lafortune L. Physical activity in community dwelling older people: a systematic review of reviews of interventions and context. *PLoS One* 2016;11:e0168614.

29. Devereux-Fitzgerald A, Powell R, Dewhurst A, French DP. The acceptability of physical activity interventions to older adults: A systematic review and meta-synthesis. *Soc Sci Med* 2016;158:14-23.

30. Zahiri CA, Schmalzried TP, Szuszczewicz ES, Amstutz HC. Assessing activity in joint replacement patients. *J Arthroplasty* 1998;13:890–5.

31. Naal FD, Impellizzeri FM, Leunig M. Which is the best activity rating scale for patients undergoing total joint arthroplasty? *Clin Orthop Relat Res* 2009;467:958-65.

32. SooHoo NF, Li Z, Chenok KE, Bozic KJ. Responsiveness of patient reported outcome measures in total joint arthroplasty patients. *J Arthroplasty* 2015;30:176-91.

33. Binkley JM, Stratford OW, Lott SA, Riddle DL. The Lower Extremity Functional Scale (LEFS): Scale development, measurement properties, and clinical application. *Phys Ther* 1999;79:371-83.

34. Dawson J, Fitzpatrick R, Carr A, Murray D. Questionnaire on the perceptions of patients about total hip replacement. *J Bone Joint Surg Br.* 1996;78:185-90.

35. Dawson J, Fitzpatrick R, Murray D, Carr A. Questionnaire on the perceptions of patients about total knee replacement. *J Bone Joint Surg Br.* 1998;80:63-9.

36. Schwarzer R, Jerusalem M. Generalized Self-Efficacy scale. In J. Weinman, S. Wright, & M. Johnston, Measures in health psychology: A user's portfolio. Causal and control beliefs 1995, pp. 35-7.

37. Miller RP, Kori S, Todd D. The Tampa Scale: a measure of kinesiophobia. *Clin J Pain* 1991;7:51–2.

38. Zigmond AS, Snaith RP. The hospital anxiety and depression scale. *Acta Psychiatrica Scandinavica* 1983;67:361–70.

39. EuroQol Group E (1990) EuroQol—a new facility for the measurement of health-related quality of life. *Health Policy* 16:199–208.

40. Ooms A, Dutton S, Parsons S, Fordham B, Hing C, Lamb SE, Smith TO. Statistical analysis plan for a pragmatic phase III randomised controlled trial examining behaviour change physiotherapy intervention to increase physical activity following hip and knee replacements: the PEP-TALK Trial. *Trials* 2021;22:467.

41. Schulz KF , Altman DG , Moher D , et al. CONSORT 2010 statement: updated guidelines for reporting parallel group randomised trials. *BMC Med* 2010;8:18.

42. Fawaz WS, Masri BA. Allowed activities after primary total knee arthroplasty and total hip arthroplasty. *Orthop Clin North Am* 2020;51:441-52.

For peer review only

43. Luna IE, Kehlet H, Wede HR, Hoevsgaard SJ, Aasvang EK. Objectively measured early physical activity after total hip or knee arthroplasty. *J Clin Monit Comput* 2019;33:509-22.

44. Hawke LJ, Shields N, Dowsey MM, Choong PFM, Taylor NF. Physical activity levels after hip and knee joint replacement surgery: an observational study. *Clin Rheumatol* 2019;38:665-74.

45. Witjes S, Gouttebarge V, Kuijer PP, van Geenen RC, Poolman RW, Kerkhoffs GM. Return to sports and physical activity after total and unicondylar knee arthroplasty: a systematic review and meta-analysis. *Sports Med* 2016;46:269-92.

46. Hamel MB, Toth M, Legedza A, Rosen MP. Joint replacement surgery in elderly patients with severe osteoarthritis of the hip or knee: decision making, postoperative recovery, and clinical outcomes. *Arch Intern Med* 2008;168:1430-40.

47. Ethgen O, Bruyère O, Richy F, Dardennes C, Reginster JY. Health-related quality of life in total hip and total knee arthroplasty. A qualitative and systematic review of the literature. *J Bone Joint Surg Am* 2004;86:963-74.

48. Smith TO, Dainty JR, Clark EM, Whitehouse MR, Price AJ, MacGregor AJ. Demographic and geographical variability in physiotherapy provision following hip and knee replacement. An analysis from the National Joint Registry for England, Wales, Northern Ireland and the Isle of Man. *Physiotherapy* 2020;106:1-11.

49. Garriga C, Leal J, Sánchez-Santos MT, Arden N, Price A, Prieto-Alhambra D, Carr A, Rangan A, Cooper C, Peat G, Fitzpatrick R, Barker K, Judge A. Geographical variation in outcomes of primary hip and knee replacement. *JAMA Netw Open* 2019;2:e1914325.

50. Fleischman AN, Crizer MP, Tarabichi M, Smith S, Rothman RH, Lonner JH, Chen AF. 2018 John N. Insall Award: Recovery of knee flexion with unsupervised home exercise is not inferior to outpatient physical therapy after TKA: a randomized trial. *Clin Orthop Relat Res* 2019;477:60-9.

51. Hamilton DF, Beard DJ, Barker KL, Macfarlane GJ, Tuck CE, Stoddart A, Wilton T, Hutchinson JD, Murray GD, Simpson AHRW; TRIO investigators. Targeting rehabilitation to improve outcomes after total knee arthroplasty in patients at risk of poor outcomes: randomised controlled trial. *BMJ* 2020;371:m3576.

52. Barker KL, Room J, Knight R, Dutton S, Toye F, Leal J, Kenealy N, Maia Schlüssel M, Collins G, Beard D, Price AJ, Underwood M, Drummond A, Lamb S; CORKA Trial group. Home-based rehabilitation programme compared with traditional physiotherapy for patients at risk of poor outcome after knee arthroplasty: the CORKA randomised controlled trial. *BMJ Open* 2021;11:e052598.

53. Smith TO, Kamper SJ, Williams CM, Lee H. Reporting of social deprivation in musculoskeletal trials: An analysis of 402 randomised controlled trials. *Musculoskeletal Care* 2021;19:180-5.

54. Smith TO, Belderson P, Dainty JR, et al. Impact of COVID-19 pandemic social restriction measures on people with rheumatic and musculoskeletal diseases in the UK: a mixed-methods study. *BMJ Open* 2021;11:e048772.

55. Brady SM, Fenton SAM, Metsios GS, Bosworth A, Duda JL, Kitas GD, Veldhuijzen van Zanten JJCS. Different types of physical activity are positively associated with indicators of mental health and psychological wellbeing in rheumatoid arthritis during COVID-19. *Rheumatol Int* 2021;41:335-44.

Figure 1: CONSORT Flow-Chart

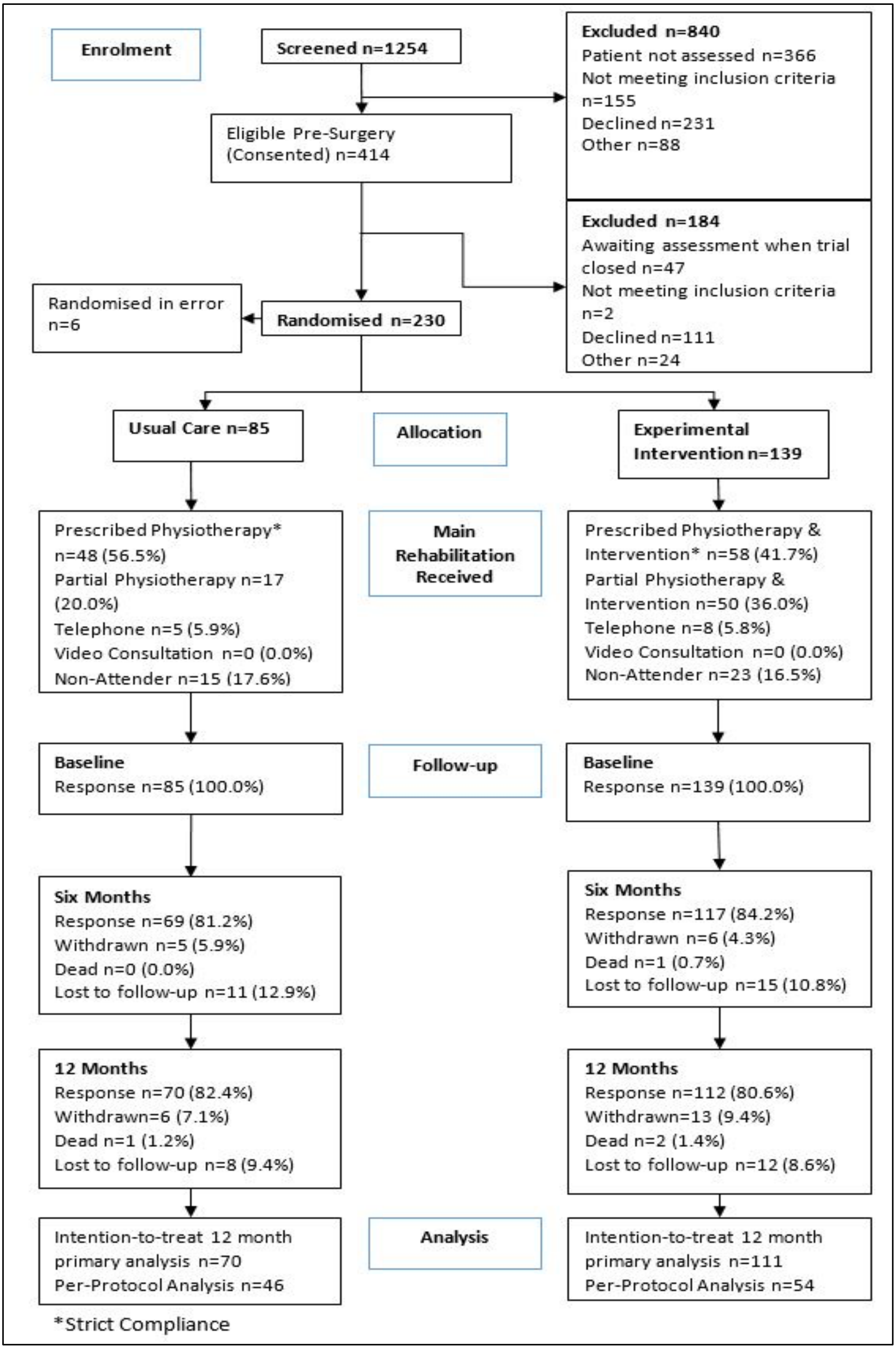

Figure 2: UCLA Activity Score boxplots

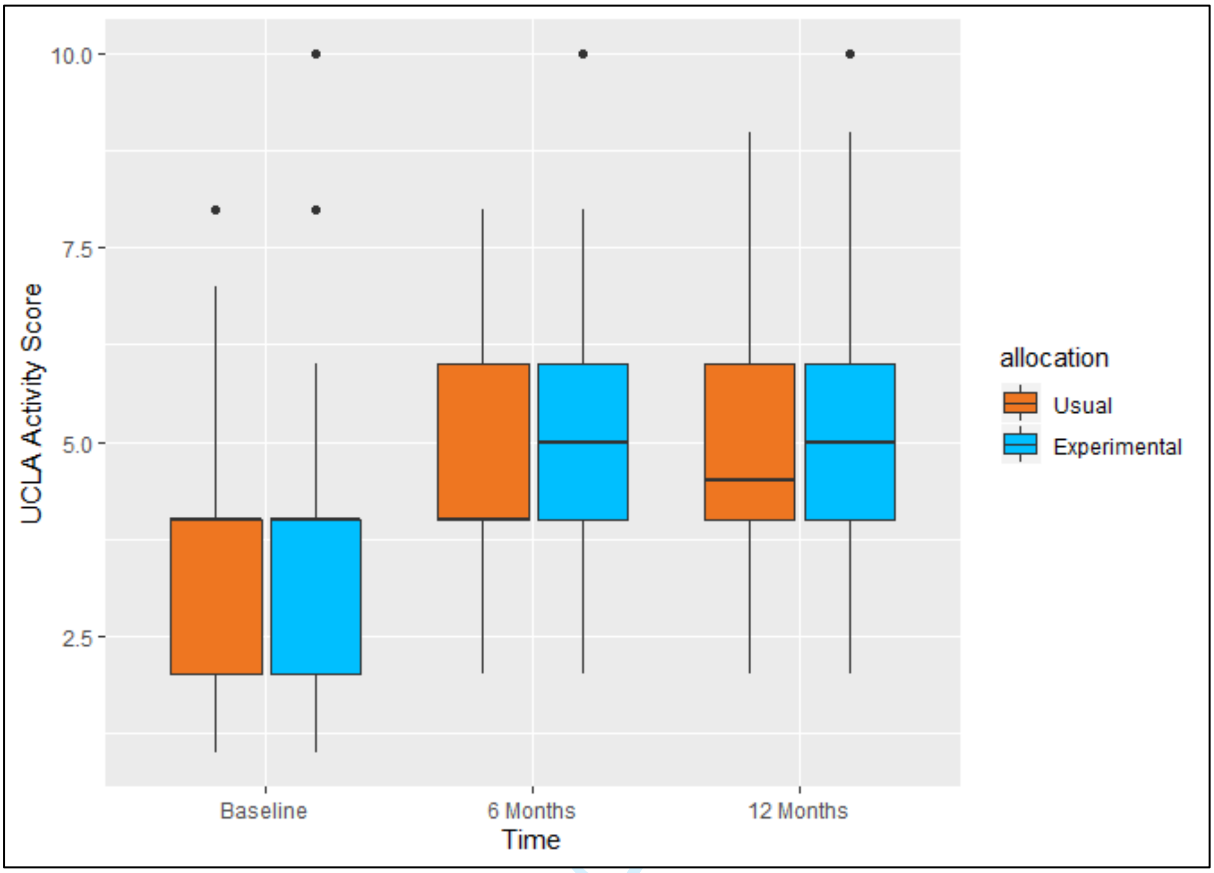

Supplementary File 1: PEP-TALK programme intervention outline and development

Background

Total hip (THR) and knee replacement (TKR) are two highly successful orthopaedic procedures which reduce pain for people with osteoarthritis (1-2). Over 206,000 THRs and TKRs were performed in the UK in 2018 (1). Approximately 90% of patients report significant improvements in pain and physical function after three to 12 months (2-3). However medical co-morbidities are common in this population. These include hypertension (56%) (4) and cardiovascular disease (20%) (5), diabetes (16%) (5) and multi-joint pain (57%) (4). Twenty-seven percent of people who undergo joint replacement have three or four comorbidities (5). These have a significant negative impact on both health-related quality of life and societal burden (6-7).

Historically, it has been assumed that people are more active following TKR and THR through the amelioration of their joint pain (8). However physical activity, for most patients, remains the same from pre- to post-operatively, and in some instances declines (8-9). Physical activity can significantly reduce the symptoms associated with common comorbidities (10). Participating in regular physical activity can decrease the risk of cardiovascular disease by 52% (11), diabetes by 65% (12) and some cancers by 40% (13). It can reduce all-cause mortality by 33% and cardiovascular mortality by 35% (14). Supporting people to be more physically active can improve patient health and decrease economic burden on health services.

A systematic review identified several barriers and facilitators associated with physical activity following TKR and THR (9). From this, four key mechanisms of action were identified for targeting. These were:

(1) Psychoeducation (knowledge/information) to increase self-efficacy.
(2) Reducing fear-avoidant behaviours in response to unhelpful beliefs about activity jeopardising recovery or damaging the implant.
(3) Providing opportunities for personal enjoyment of the physical activity.
(4) Enabling social contact, peer-support and advice from previous patients (encouraging positive coping behaviours).

Systematic reviews of behaviour change interventions have identified that those with a theoretical basis are more effective than those without (15-16). The Social Cognitive Theory (SCT) (17) has been commonly used to understand physical activity behaviour in older adults. The theory targets self-efficacy, goals, outcome expectations and socio-structural factors. Bandura (17) hypothesises that behaviour (physical activity level) is influenced by bi-directional relationships with personal factors (cognitive, emotional and physical) and environment. The cognitive behavioural approach uses techniques to identify and target unhelpful thoughts and behaviours in order to produce adaptive thoughts, behaviours, emotions and physiological responses.

Using the SCT framework, we reviewed evidence on the effectiveness of behaviour change techniques for older adults to improve physical activity. These were then compared to the systematic review regarding patients' perspectives post-TKR/THR (9) to for the four key SCT targets outlined below.

1. *Self-Efficacy: A person's belief in their own ability to perform a behaviour*

General self-efficacy: Quantitative and qualitative systematic reviews examining barriers and facilitators for older adults to increase physical activity have identified specific beliefs which could reduce an individual's general self-efficacy (9, 18-21). These include: stigma, body image (20) and ageing stereotypes (19). Unhelpful beliefs can be identified and explored using cognitive behavioural techniques to increase self-efficacy. The evidence also identified tools to increase general self-efficacy

For peer review only

which include the credibility of instructors and the information/physical activity tasks they provide (19-20, 22).

Self-efficacy to cope with barriers: Barrier identification and problem-solving are two key behaviour change techniques previously identified from the literature. Barriers can be socio-structural such as lack of access/convenience of facilities (20). Whilst these types of barriers cannot be changed by the PEP-TALK intervention, we can facilitate problem-solving strategies to help overcome such barriers.

The intervention programme will be a group-based rolling programme consisting of people in different stages of their behaviour change process. Peers may suggest ideas to other members in addition to ideas from instructors (20). Barriers may also be cognitive beliefs such as a fear of increasing physical activity in case of damaging the implant (9). These beliefs can be targeted with cognitive behavioural strategies.

Task efficacy: Previous literature has consistently reported that if someone has struggled with performing physical activity in the past, they will understandably have poor self-efficacy for performing physical activity tasks in the future (9, 23-24). We will target this by encouraging supportive environments to try exercises with physiotherapists (22), vicariously learning from other patients following THR or TKR (23) and tailored exercises to meet their individual needs (19). This should theoretically increase self-efficacy and the likelihood of greater physical activity engagement (17).

Somatic and emotional states influence self-efficacy (17). Experiencing stress/tension (emotional), fatigue and pain (somatic) can be interpreted by individuals as an indication that they cannot or should not be active. This consequently lowers their self-efficacy. This will be targeted with psychoeducation regarding relationships between mood and pain to physical activity. Conversely positive mood often increases self-efficacy. French et al (23) identified rewards contingent on attempts to perform the behaviour to be a key behaviour change technique for older adults in increasing physical activity. In our intervention, we will ensure participants are praised or rewarded for attempting to achieve their behavioural goal.

2. Goals

The SCT suggests that identifying proximal and distal goals are key to behaviour change (17). While this may be the case for younger adults, in older adults and individuals following THR or TKR specifically, goal-setting has consistently shown not to be a useful technique and not acceptable (9, 22-23). French et al (23) proposes two explanations regarding this change. Firstly, with age, cognitive process of executive functioning (planning, attentional capacity, inhibition of responses or novel actions) decreases to reduce abilities to self-regulate with goal-setting. Secondly, at this life stage, achieving set goals and normative comparison is not as pertinent as it is in earlier life. Therefore, we shall not include goal-setting in this intervention.

3. Outcome Expectation

While the motivation for this intervention may be to increase physical activity for improved health, evidence suggests that health improvement is not the salient outcome for older adults following THR or TKR. This population appear more interested in the social aspect and the enjoyment through physical activity (9). The Socioemotional Selectivity Theory (25) is a life-span theory of motivation which suggests that as people age, motivation is influenced more by positive, emotionally meaningful goals and activities and less so by normatively defined goals of health. This is extended by Devereux-Fitzgerald's (22) model of the interplay of factors of acceptability to physical activity interventions for older adults. They identified that interventions which provide the most enjoyment and meaningful value (e.g. social interactions) are the most acceptable (22). Our intervention aims to identify what is meaningful and valuable to participants by consistently asking them to reflect on open questions such as "what do you want to gain from attending this group? What are you enjoying most?" then tailoring

For peer review only

why and how to perform physical activity to meet these needs. We will also consider these factors when discussing maintenance and continuation of increased physical activity, identifying activities which are fun and enjoyable for each person. This can be aided by ideas generated from group members who may be at different stages of the behaviour change process.

4. Socio-Structural Factors

Although socio-structural factors are key to the SCT, these are aspects which we cannot change from an intervention perspective. However, we can identify modifiable factors and use problem-solving techniques to overcome barriers or find alternatives options. For example, a patient explains there is no safe pavement to walk along from their house to the shops and consequently the patient always drives. The group could offer local knowledge solutions, perhaps there is a nearby bus which can take the patient into a part of the town with good walkways. If the patient does not want to catch the bus then this belief could be explored to further understand the perceived barrier (lack of knowledge of the bus routes, perceived financial cost). This technique was identified as a key behaviour change technique for older adults in increasing physical activity (23).

In summary, while there are four key constructs in the SCT, we anticipate that self-efficacy is the key construct to target for change. A key barrier, specific to this population, to improve self-efficacy could be targeting the personal beliefs regarding fear of damaging the implant or re-injury (9). We prioritise targeting self-efficacy and fear avoidance as they are two key constructs that will change as a result of our behaviour change techniques to mediate and improve physical activity within this population.

Intervention development

The SCT provides an in-depth psychological model of why people do or do not perform behaviours. These psychological models of behaviour have been successfully synthesised into a pragmatic framework called the Capability, Opportunity, Motivation – Behaviour (COM-B) model (26). To produce the most effective behaviour change intervention, the evidence has been mapped on biopsychosocial determinants of physical activity levels post-THR/TKR from the SCT onto the COM-B model for behaviour change (*as presented in figure below*). This activity is summarised in the table below.

Capability Opportunity Motivation model of Behaviour (COM-B; Michie et al, 2014)

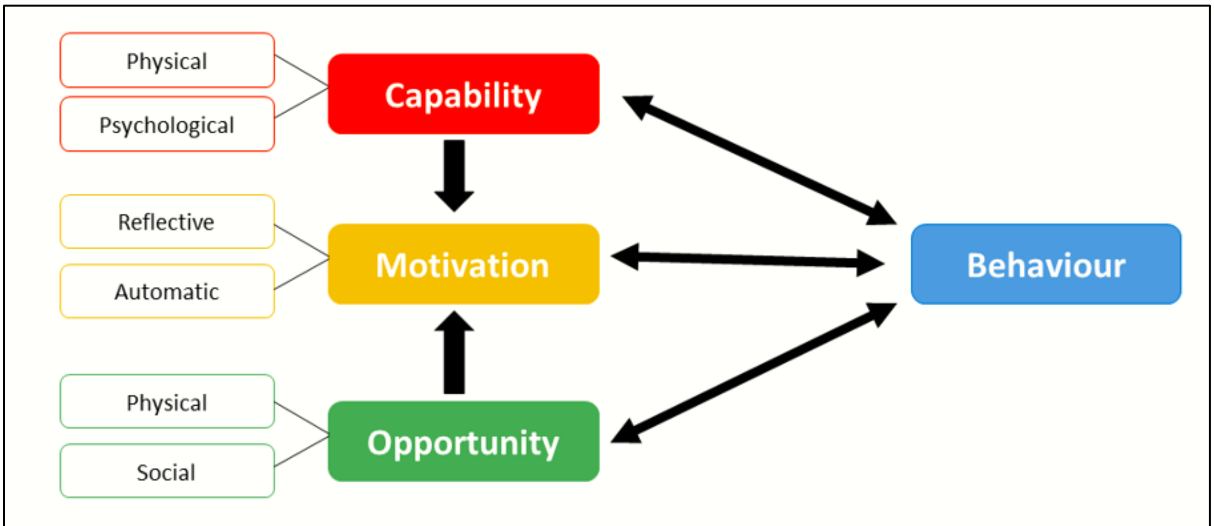

Figure 1: The COM-B model of behaviour change (Michie et al, 2014)

Mapping of the COM-B domains against the PEP-TALK SCT targets.

COM-B Model Component	Domain	Activity
Capability	Physical capability	Physiotherapeutic rehabilitation to increase the patient's capability to perform physical activities i.e. specific exercises to reduce stiffness and pain
	Psychological capability	Using cognitive behavioural techniques to increase self and task efficacy beliefs.
Opportunity	Physical opportunity	Identifying and developing problem solving techniques to overcome physical barriers to physical activity i.e. walking to a bus stop further away from the house.
	Social opportunity	Fostering solutions of how to perform physical activities in a social context i.e. communal gardening.
Motivation	Reflective	Using the PEP-TALK discussions to consciously weigh up the individual's pros and cons to performing more physical activity.
	Automatic	Developing active participation from the PEP-TALK participants to encourage linking physical activity into their daily life routine behaviours. Repetition of physically active behaviours can then become linked to everyday activities and will hopefully form into healthy habits which consistently remind, prompt and foster long-term motivation to increase physical activity.

A large proportion of the research into behaviour change techniques to increase physical activity in older adults is based on short-term (less than 12-month follow-up) data. By combining this well-developed model of intervention development, with the SCT model, and specific cognitive behavioural techniques which we have used successfully in previous interventions to increase physical activity (27-28), we hope to produce a sustained behaviour change.

Acceptability of the intervention

The evidence repeatedly recommends listening to what participants want from the intervention (20, 22-23). We aim to learn from participants what their motivations are and what will make the intervention acceptable (22).

We aim to integrate the four analytical themes from the systematic review (9) into the intervention development:

(1) Psychoeducation
(2) Reducing fear-avoidant behaviours in response to unhelpful beliefs i.e. "physical activity will damage my joint replacement"
(3) Providing opportunities for personal enjoyment of the physical activity.
(4) Enabling social contact, peer-support and advice from previous patients

To enhance the acceptability of the intervention, the social enjoyment of the group will be encouraged for making friends, as this is highly valued in older adults. Another aspect is the individual variation in the intervention exercises. This will be overcome by providing one-to-one attention, going at the participant's own pace and making the credibility of the physiotherapist and the intervention content explicit to meet the expectations and needs of older adults.

Hypothesised mediation pathway

From the literature and from our previous models of behaviour change to increase physical activity combined with physiotherapy interventions (27-28), we have developed a model of mediation. We propose that our intervention will increase physical activity levels by increasing self-efficacy and reducing fear avoidance. The pathway of mediation is outlined in the figure below. We are not specifically targeting mental health or pain experience with our intervention, but we are sensitive to monitor if increasing physical activity has a positive effect on these variables.

<u>Proposed pathway of mediation for the PEP-TALK programme</u>

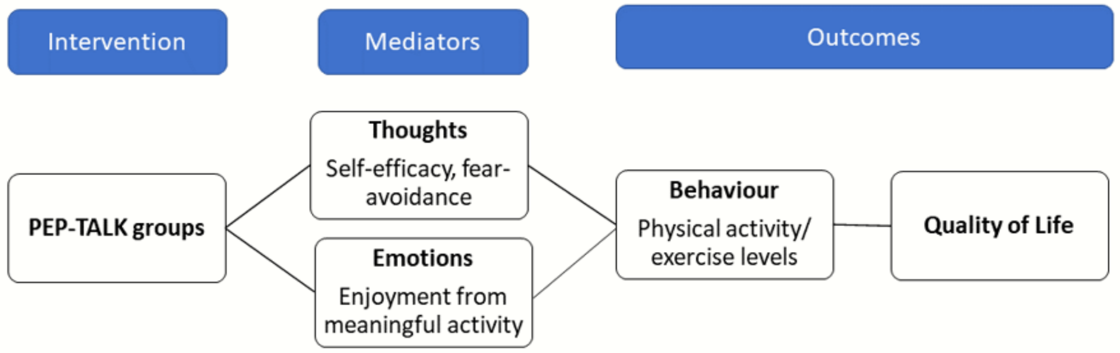

Figure 2: Proposed mediation pathway for PEP-TALK

The PEP-TALK intervention

The PEP-TALK behaviour change group will be delivered face-to-face by one physiotherapist to a group for 30 minutes. Immediately after finishing the 'talking' session the participants will begin their THR/TKR rehabilitation exercises for another 30 minutes. During the exercise session the physiotherapist will continue to talk to the participants. Asking them what they are thinking/feeling when they perform the exercises; encouraging them to reflect on their experience of pain if they encounter this. Using reflective questions to help the participants solve any barriers they encounter whilst performing the exercises. These informal encounters are used to put the theory discussed in the 'talking' group into real life practice.

At the beginning of the PRP-TALK course, intervention participants receive a printed workbook which includes information summarising the techniques, sharing examples and includes homework tasks. The homework tasks are essential for participants to practice translating the behaviour change techniques discussed in the groups, into their real lives, Reflecting on their experiences, thoughts, feelings and behaviours.

The PEP-TALK intervention, in total, lasts for one hour. The control participants only attend the THR/TKR rehabilitation exercise class, which lasts 30 minutes. The control THR/TKR exercise class includes the same physical exercises as prescribed in the intervention group's exercise class but without any of the behaviour change discussion.

<u>Methods of Delivery</u>

The PEP-TALK sessions will be delivered by a physiotherapist trained in the PEP-TALK intervention. The training consists of the PEP-TALK manual outlining the theories of behaviour change, principles of the cognitive behavioural approach, the identified barriers and facilitators to physical activity and exercises. Following this, physiotherapists will attend a one-day training session delivered by a member of the PEP-TALK programme development team (BF, ZH, TS). In this, physiotherapists will discuss the theoretical underpinning of the programme and be provided with case studies and

examples of how the PEP-TALK intervention is designed to be prescribed, and discussion on potential threats to fidelity. We will role play some patient-physiotherapist interactions to provide practical experiences of the intervention in a supportive environment. The trainers will assess how well physiotherapists follow the intervention and will acknowledge any deviations to correct practice.

The PEP-TALK intervention is delivered immediately prior to an exercise group. By timing the interventions with the group discussion first, participants will immediately action and re-enforce the encouragement for physical activity participations through exercising. We have stipulated a maximum PEP-Talk group size of 12 participants to prevent participants from becoming lost in the group and to parallel the standard usual care group size.

A group rather than a one-to-one approach has the advantage of enabling collaborative and vicarious learning, which can improve self-efficacy regarding their goal behaviour (i.e. increased physical activity), whilst also providing lower unit-costs of delivery (29). The principles underpinning this derive from Bandura et al's (17) SCT regarding vicarious learning where learning is proposed to not be acquired through direct experience but by observing other people's actions and consequences (modelling). Secondly, the principles of social cognitive development theory (30) are adopted where knowledge is acquired through guided collaboration with people who already have the knowledge. Collaborative learning with 'peers' and expert people (facilitators) helps bridge distance between an individual's level of skill and their potential, the 'zone of proximal development' (30).

Participants and physiotherapists will be encouraged to develop a positive therapeutic alliance where the physiotherapist will generate an environment of trust and belief around the individual challenges the patient has and to support them to overcome these for sustained physical activity adoption. Evidence has highlighted the beneficial impact of a positive therapeutic alliance on outcomes within physiotherapy practice (31). Due to the nature of identifying individual's helpful and unhelpful thoughts, barriers and facilitators and strategies, the intervention has flexibility in the intention to support this approach. Therefore, whilst the intervention described below has key set-elements which form the content of sessions, there will be opportunity for individuals to express meaningful thoughts and experiences to them, thereby personalising the intervention.

Where Delivered

The PEP-TALK behaviour change group and subsequent exercise sessions will be delivered in an out-patient physiotherapy gym environment. Participants will be sat in a circle to facilitate dialogue. Following the 'talking' intervention, participants begin their THR/TKR exercise session. They will perform exercises in exercise stations, monitored by a trained physiotherapist.

The PEP-TALK behaviour change programme consists of six sessions (A-F) delivered as a rolling programme. Once a new participant has been randomised they can join the groups in any session: A, B, C, D, E or F. Consequently, in every session delivered there will be a mixture of participants who have attended 5,4,3,2,1 or 0 previous PEP-TALK sessions. This necessitates a large amount of repetition of the aims and techniques in every session to ensure all members of the group understand the core behaviour change messages. The rolling programme also enables groups to run continuously, minimising a participant's waiting time to join a group.

A treatment log will be completed by the physiotherapists to record the component of what is discussed across the participants group in each of the session.

Group session will be re-enforced with a participant workbook. This provides participants with salient information from each session, and provides them with exercise progressions, an exercise diary, a guide and space to complete homework tasks/record.

Content of PEP-TALK Sessions

For peer review only

Each of the six PEP-TALK sessions (A - F) will follow this structure:

(1) agenda setting – what will be covered in the session
(2) today's session – covering topics which have been demonstrated to impact on physical activity following joint replacement (content listed below)
(3) conclusion – provision of homework and summarising topics covered today and what will be covered in the next session
(4) break - before commencing exercises group session

There is a degree of overlap between sessions to aid reinforcement of ideas and beliefs. This overlap is largely on identification of barriers and discussion of progress for individuals to share. The principles around the six sessions are presented below:

1. <u>"Being Physically Active": Individual's meaning of physical activity and barriers and problem-solving</u>
    a. Exploring what physical activity means to each participant. For example: active living, transport, sports and exercise. Consideration by participants of what proportion of their lives are engaged with each aspect of physical activity and what the harms and benefits are of being inactive and active. Participants consider what potential barriers exists to activity and whether they want to address these barriers.

2. <u>"Gradually increasing physical activity": Under/Over-Activity, Pacing, Graded Activities</u>
    a. In this session individuals will be taught the principles of pacing and graded-activity. Discussion will be centred on an example e.g. cleaning the car and how pacing and graded-activity could be implemented. The concept of determining a 'baseline' of activity will be established. Individuals will be asked to consider what challenges they have to implementing a graded-activity programme in everyday activities. To facilitate this, individuals will be asked to consider another activity and work through how that activity may be paced in the following week.

3. <u>"Should I be doing this?" : Fear-avoidance</u>
    a. This session will focus on education on avoidance of activity and why individuals avoid activities in relation to their recovery and protection of a joint replacement. Consideration will be focused on thoughts which could be challenged particularly in relation to functional tasks such as washing and dressing, walking, sports or home activities. Individuals will consider how fear avoidance is a circular behaviour in relation to 'thoughts', 'feelings', 'actions', 'results' which can reinforce health beliefs around activity avoidance but acknowledging that such a cycle is a normal response given their previous pain. Discussion will be made for individuals to consider how they may overcome these beliefs.

4. <u>"Physical activity benefits" : Emotion and Sleep, Exercise, Social links</u>
    a. Exploration on the benefits of physical activity on emotional health and sleep will form the basis of this session. Individuals will be asked to consider how being less depressed, stressed and sleep deprived and happier with greater social contact can affect their lives. They will consider how these factors inhibit their ability to be more physically active. Discussion will be made on how worry may relate to pain and what strategies they must address this. Individuals will also think about challenging beliefs around failure to be able to complete certain activities and what their own fears are regarding being more or less active.

5. <u>"Can I change how I think?": Worry, Distraction, Unhelpful Thoughts</u>

For peer review only

   a. Fears and worries about jeopardizing recovery and long-term joint health will be explored in this session. Individuals will identify and challenge beliefs around physical activity and harm or damage which are unhelpful thoughts. They will explore a 'vicious cycle' notion where unhelpful thinking leads to feeling low, leading to feeling unmotivated, leading to reduced physical activity leading to atrophy which reinforces the unhelpful thought. Individuals will be asked to consider 'answer back thoughts' and strategies to address such unhelpful thoughts and distractions.

6. "Staying active and having fun" : Social and Rewarding
   a. The benefits of physical activity as a reward will be explored in this session. They will be asked to consider what activities they do alone, and which could be done with others, to increase social contact and increase motivation and pleasure from participating in an activity. Individuals will consider potential barriers and strategies to promote and adopt such an approach to everyday activities' which interest them.

Homework Activities

Participants will be supported with skills developed in the group, to work at home on challenges, barriers and facilitators to physical activity behaviour. The 'home-work' after each session will include pacing and behaviour modification, goal-setting to the individual's health and social needs, and techniques to challenge fear avoidant behaviours.

Follow-up Telephone Calls

Three follow-up telephone calls (maximum 20-minute duration) will be undertaken at two, four and six weeks following the last group session. Follow-up telephone calls are an important element of the behaviour change intervention. They will review participant's goals, identifying any barriers to the completion of these goals, and review any 'helpful' and 'unhelpful' thoughts or feelings towards physical activity which may have arisen since the last consultation. Each telephone call will close with the development of longer-term physical activity plans and promotion of empowerment towards physical activity participation using these behavioural principles instilled during the group intervention.

Adherence and Fidelity

The PEP-TALK team phone the physiotherapist delivering the intervention group after their first session has been delivered. The aim of this call is to address any problems the physiotherapist may have encountered and for the PEP-TALK team to offer solutions and tips. After the third session has been delivered, a member of the PEP-TALK team visit the site and observe a PEP-TALK behaviour change and exercise session to perform a quality assessment (QA). If there are quality concerns, then the site will receive additional training and another QA visit will be undertaken.

At a participant level, compliance to the PEP-TALK intervention will be arbitrarily met with participants required to attend 70% of the behaviour-change and exercise groups and 66% of the telephone calls.

Access to the Intervention

The PEP-TALK intervention manual and work-book will be available on completion of the trial. This can be accessed through the corresponding author.

Conclusions

The development and content of the PEP-TALK intervention has been presented. This addresses key modifiable risk factors to physical inactivity following hip and knee replacement. The effectiveness of

For peer review only

this intervention will now be assessed in the multi-centre, pragmatic, randomised controlled trial (PEP-TALK Trial).

SUPPLEMENTARY FILE 1: REFERENCES

1. National Joint Registry. 2018. Reports Online 2018. National Joint Registry UK. Accessed: 22 July 2019. Available at: http://www.njrcentre.org.uk/njrcentre/Healthcare-providers/Accessing-the-data/ReportsOnline
2. Dailiana ZH, Papakostidou I, Varitimidis S, Liaropoulos L, Zintzaras E, Karachalios T, Michelinakis E, Malizos KN. Patient-reported quality of life after primary major joint arthroplasty: a prospective comparison of hip and knee arthroplasty. BMC Musculoskelet Disord. 2015;16:366.
3. Papakostidou I, Dailiana ZH, Papapolychroniou T, Liaropoulos L, Zintzaras E, Karachalios TS, Malizos KN. Factors affecting the quality of life after total knee arthroplasties: a prospective study. BMC Musculoskelet Disord. 2012;13:116.
4. Inacio MC, Pratt NL, Roughead EE, Graves SE. Comparing co-morbidities in total joint arthroplasty patients using the RxRisk-V, Elixhauser, and Charlson Measures: a cross-sectional evaluation. BMC Musculoskelet Disord. 2015;16:385.
5. Peter WF, Dekker J, Tilbury C, Tordoir RL, Verdegaal SH, Onstenk R, Bénard MR, Vehmeijer SB, Fiocco M, Vermeulen HM, van der Linden-van der Zwaag HM, Nelissen RG, Vliet Vlieland TP. The association between comorbidities and pain, physical function and quality of life following hip and knee arthroplasty. Rheumatol Int. 2015;35:1233-41.
6. Martinez-Cano JP, Herrera-Escobar JP, Arango Gutierrez AS, Sanchez Vergel A, Martinez-Rondanelli A. Prospective quality of life assessment after hip and knee arthroplasty: short- and mid-term follow-up results. Arthroplast Today. 2016;3:125-130.
7. Sampson UK, Fowkes FG, McDermott MM, Criqui MH, Aboyans V, Norman PE, Forouzanfar MH, Naghavi M, Song Y, Harrell FE Jr, Denenberg JO, Mensah GA, Ezzati M, Murray C. Smith E, Hoy DG, Cross M, Vos T, Naghavi M, Buchbinder R, Woolf AD, March L. PICO. The global burden of other musculoskeletal disorders: estimates from the Global Burden of Disease 2010 study. Ann Rheum Dis. 2014;73:1462-9.
8. Withers TM, Lister S, Sackley C, Clark A, Smith TO. Is there a difference in physical activity levels in patients before and up to one year after unilateral total hip replacement? A systematic review and meta-analysis. Clin Rehabil. 2017;31:639-650.
9. Smith TO, Latham S, Maskrey V, Blyth A. Patients' perceptions of physical activity before and after joint replacement: a systematic review with meta-ethnographic analysis. Postgrad Med J. 2015;91:483-91.
10. Global Burden of Disease Study 2013 Collaborators. Global, regional, and national incidence, prevalence, and years lived with disability for 301 acute and chronic diseases and injuries in 188 countries, 1990–2013: a systematic analysis for the Global Burden of Disease Study 2013. Lancet. 2015;386:743–800
11. Lee CD, Folsom AR, Blair SN. Physical activity and stroke risk: a meta-analysis. Stroke. 2003;34:2475-81.
12. Laaksonen DE, Lindström J, Lakka TA, Eriksson JG, Niskanen L, Wikström K, et al; Finnish diabetes prevention study. Physical activity in the prevention of type 2 diabetes: the Finnish diabetes prevention study. Diabetes. 2005;54:158-65.
13. Lee IM. Physical activity and cancer prevention--data from epidemiologic studies. Med Sci Sports Exerc. 2003;35:1823-7.
14. Nocon M, Hiemann T, Müller-Riemenschneider F, Thalau F, Roll S, Willich SN. Association of physical activity with all-cause and cardiovascular mortality: a systematic review and meta-analysis. Eur J Cardiovasc Prev Rehabil. 2008;15:239-46.

For peer review only

15. Abraham C and Michie, S. A taxonomy of behaviour change techniques used in interventions. Health Psychology, 2008;27:379-87.
16. Chase J. A. Interventions to increase physical activity among older adults: a meta-analysis. The Gerontologist, 2015;55:706-18.
17. Bandura A. Social foundations of thought and action: A social cognitive theory. Prentice-Hall, Inc, 1986.
18. Baranowski T, Anderson C and Carmack C. Mediating variable framework in physical activity intervetnions: How are we doing? how might we do better? Am J Prevent Med. 1998;15;266-97.
19. Arnautovska U, O'Callaghan F, Hamilton K. Applying the Integrated Behavior Change Model to Understanding Physical Activity Among Older Adults: A Qualitative Study. J Sport Exerc Psychol. 2017;39:43-55.
20. Olanrewaju O, Kelly S, Cowan A, Brayne C, Lafortune L. Physical activity in community dwelling older people: a systematic review of reviews of interventions and context. PLoS One. 2016;11:e0168614.
21. Withers TM, Lister S, Sackley C, Clark A, Smith TO. Is there a difference in physical activity levels in patients before and up to one year after unilateral total hip replacement? A systematic review and meta-analysis. Clin Rehabil. 2017;31:639-50.
22. Devereux-Fitzgerald A, Powell R, Dewhurst A, French DP. The acceptability of physical activity interventions to older adults: A systematic review and meta-synthesis. Soc Sci Med. 2016;158:14-23.
23. French DP, Olander EK, Chisholm A, Mc Sharry J. Which behaviour change techniques are most effective at increasing older adults' self-efficacy and physical activity behaviour? A systematic review. Ann Behav Med. 2014;48:225-34.
24. Pekmezi DW, Neighbors CJ, Lee CS, Gans KM, Bock BC, Morrow KM, Marquez B, Dunsiger S, Marcus BH. A culturally adapted physical activity intervention for Latinas: a randomized controlled trial. Am J Prev Med. 2009;37:495-500.
25. Carstensen LL. Motivation for social contact across the life span: a theory of socioemotional selectivity. Nebr Symp Motiv. 1992;40:209-54.
26. Michie S, Atkins L, West R. The Behaviour Change Wheel: A guide to designing Interventions. Silverback Publishing. 2014
27. Lamb SE, Lall R, Hansen Z, Castelnuovo E, Withers EJ, Nichols V, Griffiths F, Potter R, Szczepura A, Underwood M; BeST trial group. A multicentred randomised controlled trial of a primary care-based cognitive behavioural programme for low back pain. The Back Skills Training (BeST) trial. Health Technol Assess. 2010;14:1-253.
28. Lamb 2015. Better outcomes for older people with spinal trouble. Accessed: 24 May 2018. Available at: http://www.isrctn.com/ISRCTN12698674
29. Critchley DJ, Ratcliffe J, Noonan S, Jones RH, Hurley MV. Effectiveness and cost-effectiveness of three types of physiotherapy used to reduce chronic low back pain disability: a pragmatic randomized trial with economic evaluation. Spine 2007;32:1474–81.
30. Vygotsky L. Interaction Between. Learning and Development. In Gauvain and Cole (eds) Readings on the Development of Children. New York: Scientific. American Books. pp. 34-40, 1978.
31. Paulo H. Ferreira, Manuela L. Ferreira, Christopher G. Maher, Kathryn M. Refshauge, Jane Latimer, Roger D. Adams; The therapeutic alliance between clinicians and patients predicts outcome in chronic low back pain. Phys Ther 2013;93:470–8.

For peer review only

Supplementary File 2: Additional results

Pre-Specified Definition of Compliance

Compliance was defined in three nested levels for both randomised groups. These are:

Strict Compliance (as defined in the original Protocol):
Usual Care group
• Attends at least 4 out of 6 physiotherapy sessions

Experimental Intervention group
• Attends at least 4 out of 6 group intervention sessions with a minimum of 3 participants per session
• Received 2 out of 3 follow-up telephone calls

Compliance:
Usual Care group
• Attends at least 4 out of 6 physiotherapy sessions

Experimental Intervention group
• Attends at least 4 out of 6 group intervention sessions with a minimum of 3 participants per session

Attendance:
Usual Care group
• Attends at least 1 out of 6 physiotherapy sessions

Experimental Intervention group
- Attends at least 4 out of 6 group intervention sessions.

Additional Results

A summary of withdrawals is provided in **Supplementary Table 1**.

The primary analysis is performed assuming the data is missing at random (MAR). To assess the MAR assumption, varying scores of the UCLA Activity Score for all time points were imputed where data is missing and these "complete" datasets were reanalysed, using the same mixed effects as used in the primary analysis. For each missing data point, the median value of the group that participant belongs to is imputed and the imputed dataset analysed. The analysis is repeated on a population that has the 60th quantile imputed for one group's missing values and the 40th quantile for the other, then again using the 70th and 30th quantiles, up to 90th and 10th quantiles. The process was repeated but flipping the groups. In total nine sensitivity analyses were performed and the results displayed graphically in **Supplementary Figure 5**. This method used simple imputation of these quantiles, therefore the estimates of the variance will be effected, and so will all p-values and Confidence Intervals reported. **Supplementary Figure 5** shows that there would need to be an implausibility large departure from the missing at random assumption to see a statistically significant result in either direction with a result only being yielded if the 10th and 90th percentiles are imputed into each treatment group. This suggests the result from the primary analysis is robust to missing data and adds support to the findings from the primary analysis.

For peer review only

A sensitivity analysis on the per-protocol population has been performed to assess the internal validity of the trial's primary results. The analysis is based on the same mixed effects analysis model as used for the primary outcome but for the Per-Protocol population as described in the Statistical Analysis Plan.[35] To be considered per-protocol participants must have data on the UCLA Activity Score at 12 months, cannot be "Non-Compliant", cannot be part of the COVID-19 group (as these participants did not complete their intervention per-protocol), did not crossover randomised treatments and did not have any Important protocol deviations reported. Results from this analysis are reported in **Supplementary Table 3**. The per-protocol analysis reinforces the main trial result findings, there is no between group difference.

An analysis on the primary outcome using a reduced version of the primary analysis model, only using person as a random effect has been performed. The results are presented in **Supplementary Table 4**. The results from the reduced model in **Supplementary Table 4** are extremely similar the primary analysis results. The Akaike Information Criterion (AIC) for the primary analysis model was 1,372.47 whereas the AIC for the reduced model was 1,370.84 suggesting a marginally better model fit with centre removed.

All subgroup analyses are on the primary outcome only. Subgroup analyses of the two clinical stratifying variables (type of operation and (THR or TKR), Charlson Comorbidity Index Score (1–3 or ≥ 4)) were performed as well as a subgroup analysis on COVID-19 status (Pre-COVID-19 or COVID-19). These used an extended primary analysis model including an interaction term between treatment and each stratifying variable/COVID-19 status to define the subgroups. These analyses are exploratory, and results should be interpreted with due caution. The results will be presented in a **Supplementary Figure 6**.

Supplementary Figure 1 gives a plot of complication type.

Descriptive statistics for the Generalized Self-Efficacy Scale, Tampa Scale for Kinesiophobia, Hospital Anxiety and Depression Score, EQ-5D-5L Index, EQ-VAS and Numerical Rating Scale for Pain are given by COVID-19 status in **Supplementary Table 5**, no formal analysis is performed. The presentation of these results was pre-specified in the analysis plan and aid in assessing the impact of the COVID-19 pandemic on the trial participants. Results indicate potentially higher levels of anxiety, depression and kinesiophobia at six-months in the COVID-19 population, these apparent differences were not sustained to the 12-month follow-up. Observed self-efficacy scores were lower in the COVID-19 group across all follow-up time points. Other measures did not indicate any noticeable between group difference. These results should be interpreted with great caution due to small sample size, non-random groups, and the exploratory nature of the results.

For peer review only

Supplementary Table 1: Withdrawals summary

	Usual (n=13)	Experimental (n=24)	Total (n=37)
**Treatment Non-Compliance Reason**			
Complete withdrawal from the study and use of data	2	2	4
Withdrawal from intervention and completion of questionnaires	4	11	15
Withdrawal from intervention only	7	11	18
**Withdrawal Time Point**			
6 Months	12	17	29
12 Months	1	7	8

N - number of participants

For peer review only

Supplementary Table 2: Questionnaire returns by treatment group

Time Point	Usual	Experimental	Cumulative missing data	Total with data
Baseline	85 (100.0)	139 (100.0)	0 (0.0)	224 (100.0)
6 Months	69 (81.2)	117 (84.2)	38 (17.0)	186 (83.0)
12 Months	70 (82.4)	112 (80.6)	42 (18.8)	182 (81.2)

All data frequency and (%)

For peer review only

Supplementary Table 3: UCLA Activity Score per-protocol results

Time Point	Usual	Experimental	Mean Difference	
	n, Mean (SD)	n, Mean (SD)	Unadjusted	Adjusted (95% CI)
Baseline	n=46, 3.76 (1.51)	n=54, 3.67 (1.65)	-0.09	
6 Months	n=44, 4.91 (1.44)	n=50, 5.18 (1.86)	0.27	0.43 (-0.23,1.08)
12 Months	n=46, 5.04 (1.59)	n=54, 4.83 (1.79)	-0.21	-0.17 (-0.81,0.48)

CI - confidence intervals; N – number of participants; SD – standard deviation

For peer review only

Supplementary Table 4: UCLA Activity Score reduced model (no recruiting centre random effect) results

Time Point	Usual	Experimental	Mean Difference	
	n, Mean (SD)	n, Mean (SD)	Unadjusted	Adjusted (95% CI)
Baseline	n=85, 3.62 (1.52)	n=138, 3.57 (1.57)	-0.06	
6 Months	n=69, 4.77 (1.52)	n=117, 4.97 (1.68)	0.20	0.28 (-0.21,0.76)
12 Months	n=70, 4.87 (1.61)	n=111, 4.84 (1.91)	-0.03	-0.03 (-0.52,0.46)

CI - confidence intervals; N – number of participants; SD – standard deviation

For peer review only

Supplementary Table 5: Descriptive results for selected secondary outcomes by COVID-19 status

	Pre-COVID-19<br>n, Mean (SD)	COVID-19<br>n, Mean (SD)
**Generalized Self-Efficacy Scale**		
Baseline	n=153, 31.82 (5.49)	n=69, 30.90 (5.24)
6 Months	n=112, 33.04 (5.22)	n=44, 31.50 (5.29)
12 Months	n=112, 32.83 (6.27)	n=50, 30.74 (6.13)
**Tampa Scale for Kinesiophobia**		
Baseline	n=153, 40.09 (7.81)	n=68, 39.38 (7.20)
6 Months	n=103, 34.86 (7.79)	n=44, 35.82 (6.62)
12 Months	n=103, 35.57 (8.30)	n=44, 35.80 (6.50)
**Hospital Anxiety and Depression Scale (Overall)**		
Baseline	n=154, 11.99 (6.38)	n=69, 12.83 (7.46)
6 Months	n=110, 8.65 (6.20)	n=46, 9.39 (6.89)
12 Months	n=113, 9.46 (6.95)	n=47, 9.38 (6.60)
**Hospital Anxiety and Depression Scale (Anxiety)**		
Baseline	n=154, 6.19 (3.84)	n=69, 6.71 (4.24)
6 Months	n=112, 4.79 (3.55)	n=46, 5.33 (4.16)
12 Months	n=113, 5.11 (3.75)	n=48, 5.40 (3.95)
**Hospital Anxiety and Depression Scale (Depression)**		
Baseline	n=155, 5.83 (3.40)	n=69, 6.12 (3.95)
6 Months	n=113, 3.89 (3.31)	n=47, 4.09 (3.66)
12 Months	n=115, 4.30 (3.97)	n=48, 4.23 (3.44)
**EQ-5D-5L Index**		
Baseline	n=155, 0.40 (0.24)	n=69, 0.38 (0.28)
6 Months	n=129, 0.68 (0.25)	n=56, 0.69 (0.23)
12 Months	n=128, 0.67 (0.26)	n=55, 0.68 (0.29)
**EQ-VAS**		
Baseline	n=155, 62.34 (21.77)	n=69, 57.55 (23.07)
6 Months	n=130, 71.84 (20.74)	n=55, 75.02 (16.28)
12 Months	n=124, 73.19 (19.85)	n=55, 71.82 (17.62)
**Numerical Rating Scale for Pain**		
Baseline	n=155, 7.09 (1.87)	n=69, 7.10 (1.82)
6 Months	n=115, 3.55 (2.72)	n=47, 3.28 (2.59)
12 Months	n=112, 3.68 (2.88)	n=51, 3.47 (2.87)

N – number of participants; SD – standard deviation

Supplementary Figure 1: Complication type by randomised group

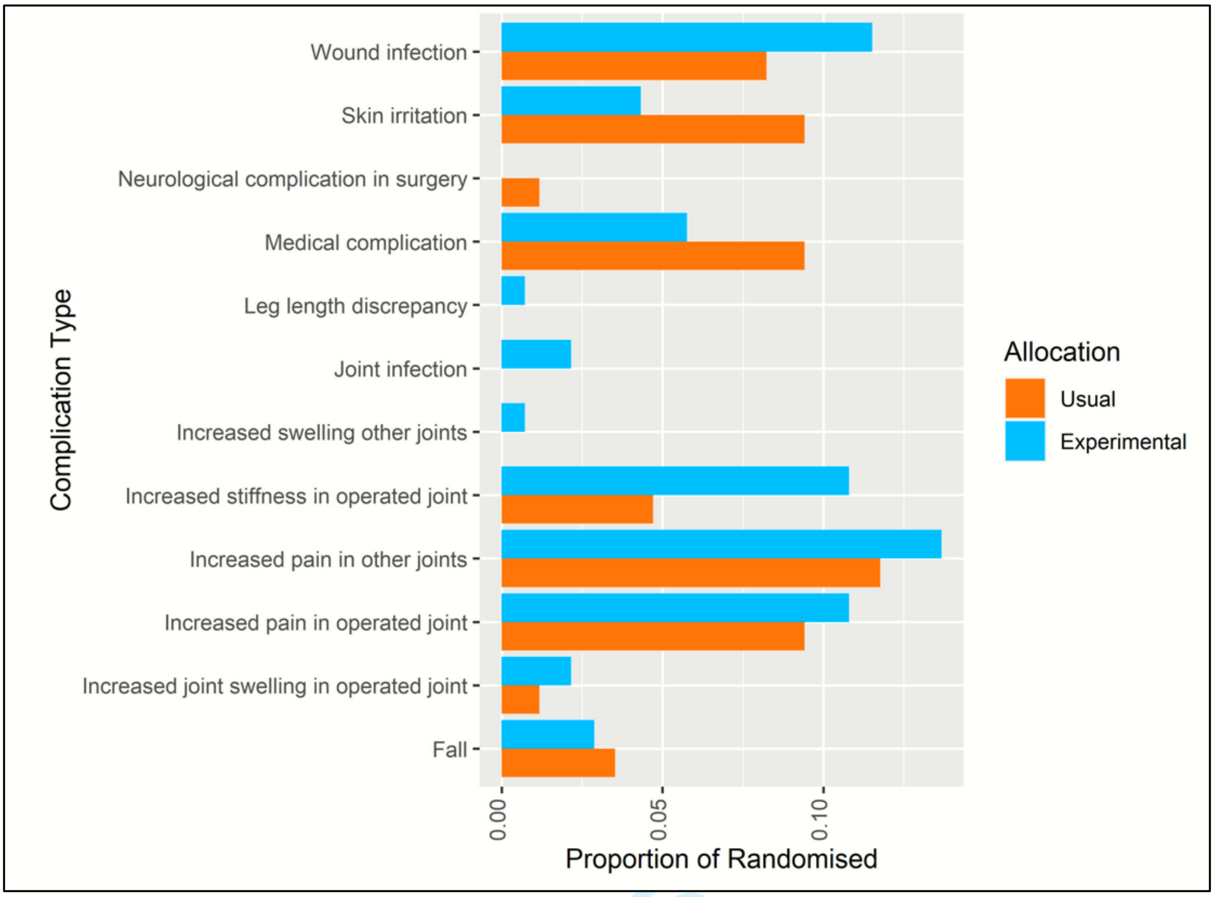

Supplementary Figure 2: Overall compliance by (a) raw frequencies and (b) percentage of randomised group

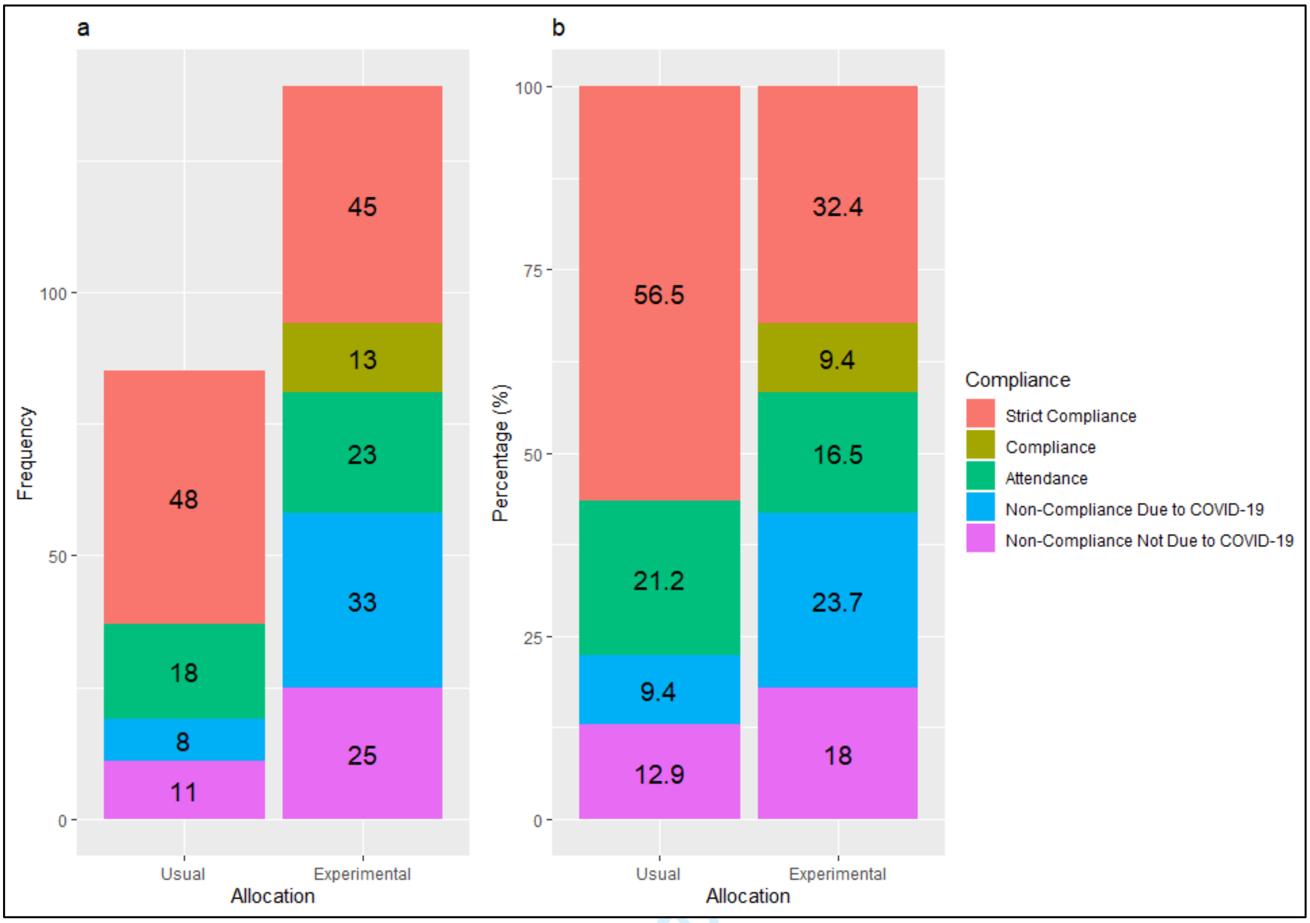

Supplementary Figure 3: Experimental intervention group sizes over time, including change from a randomisation ratio of 1:1 to 2:1

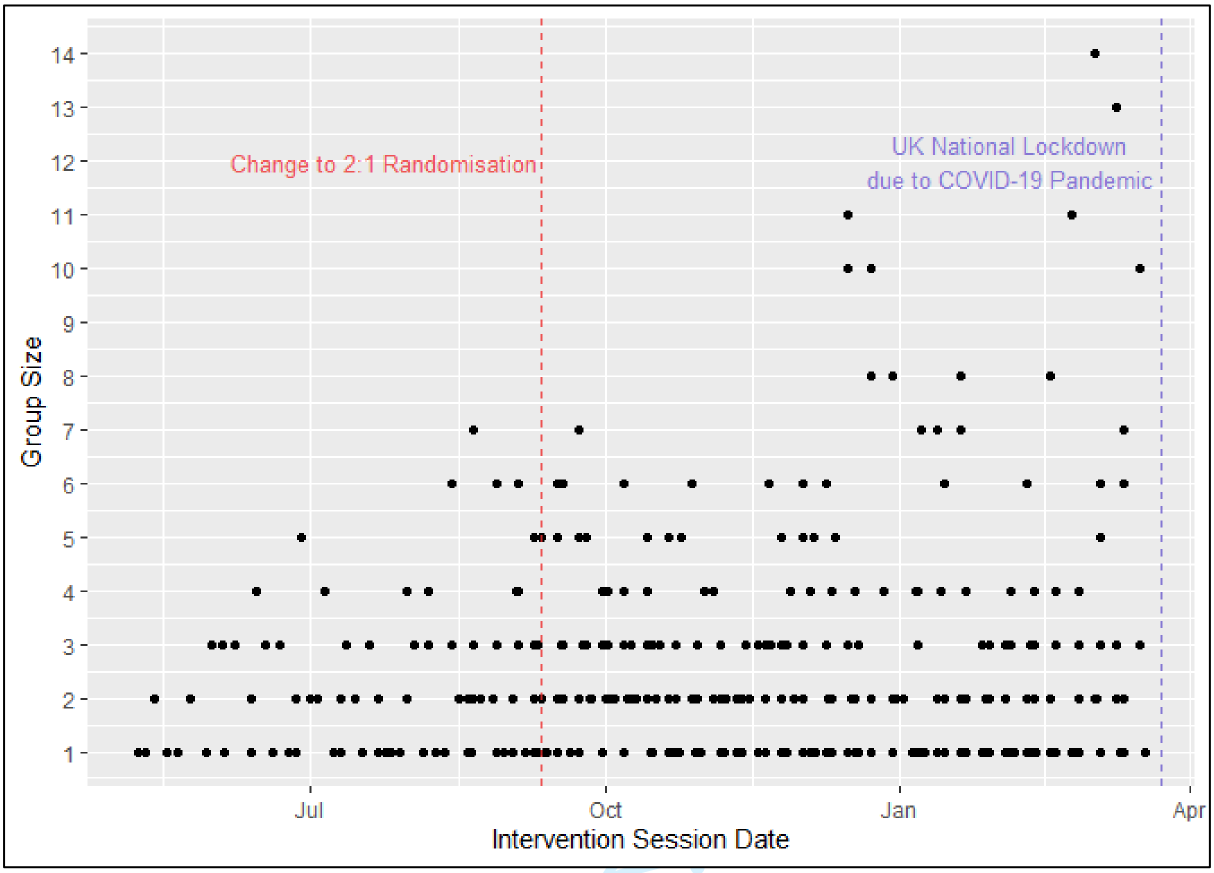

Supplementary Figure 4: Experimental intervention group compliance by COVID-19 group

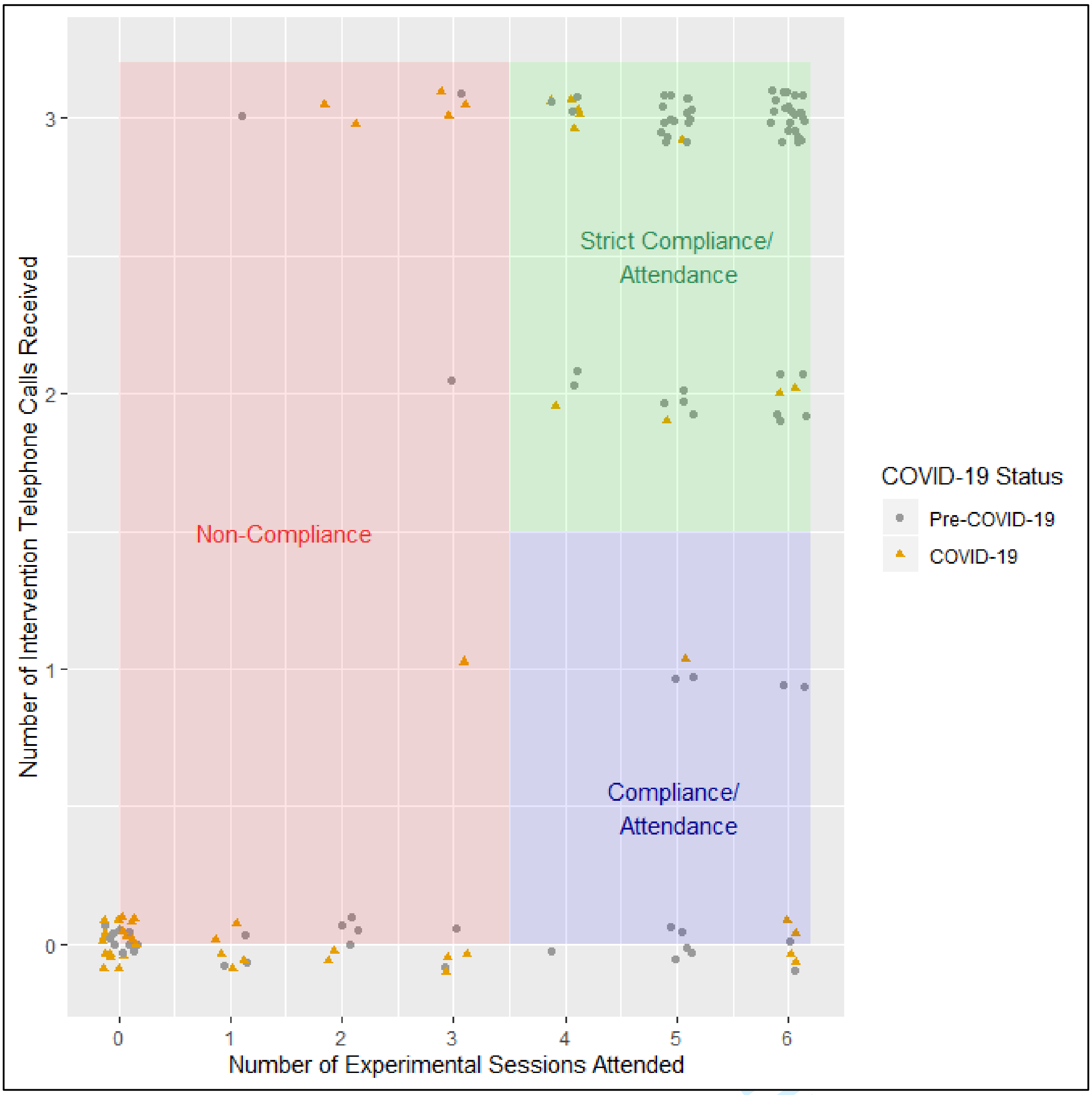

Supplementary Figure 5: 12 month adjusted mean difference UCLA Activity Score for varying imputed quantiles for missing data

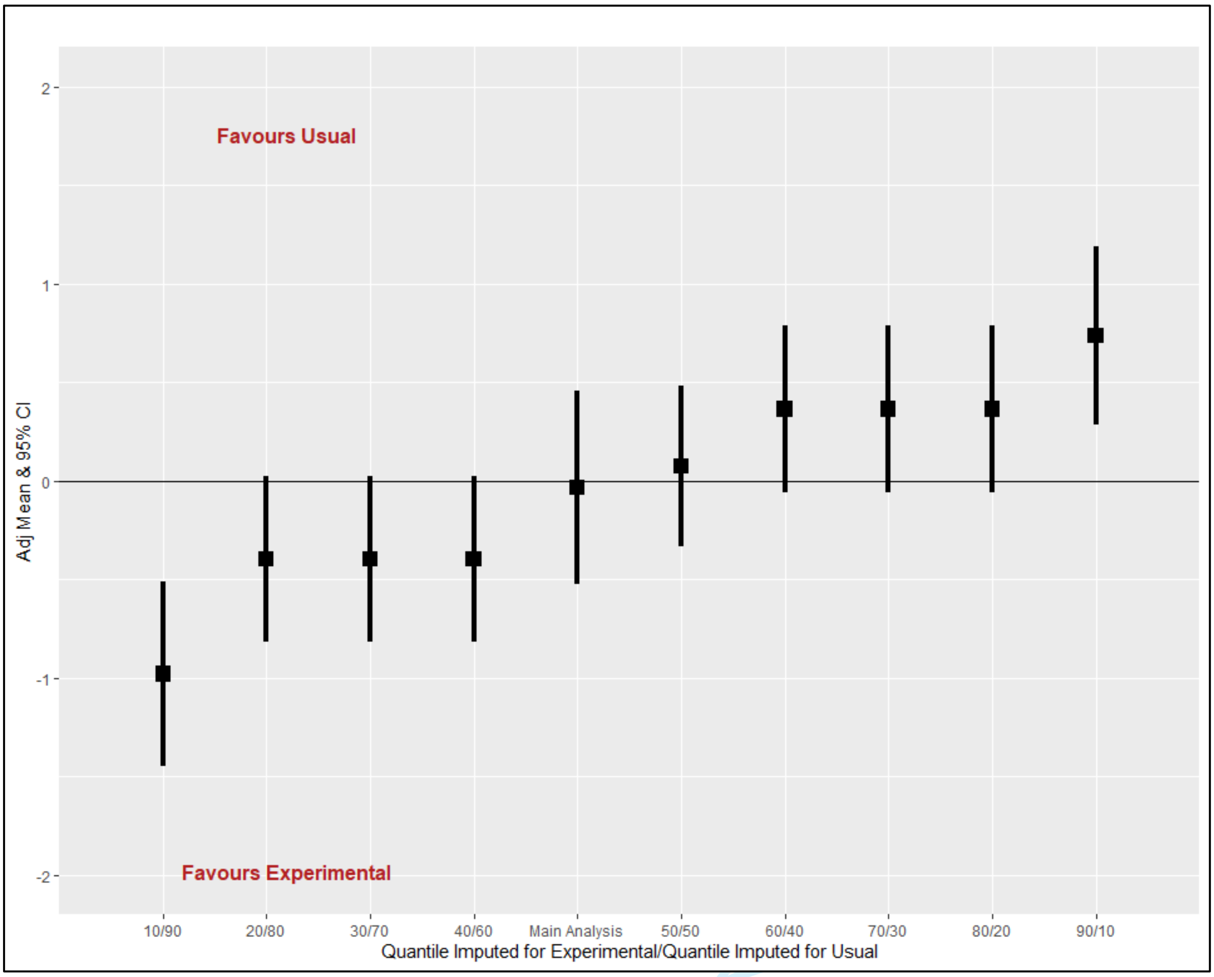

Supplementary Figure 6: Subgroup analyses results

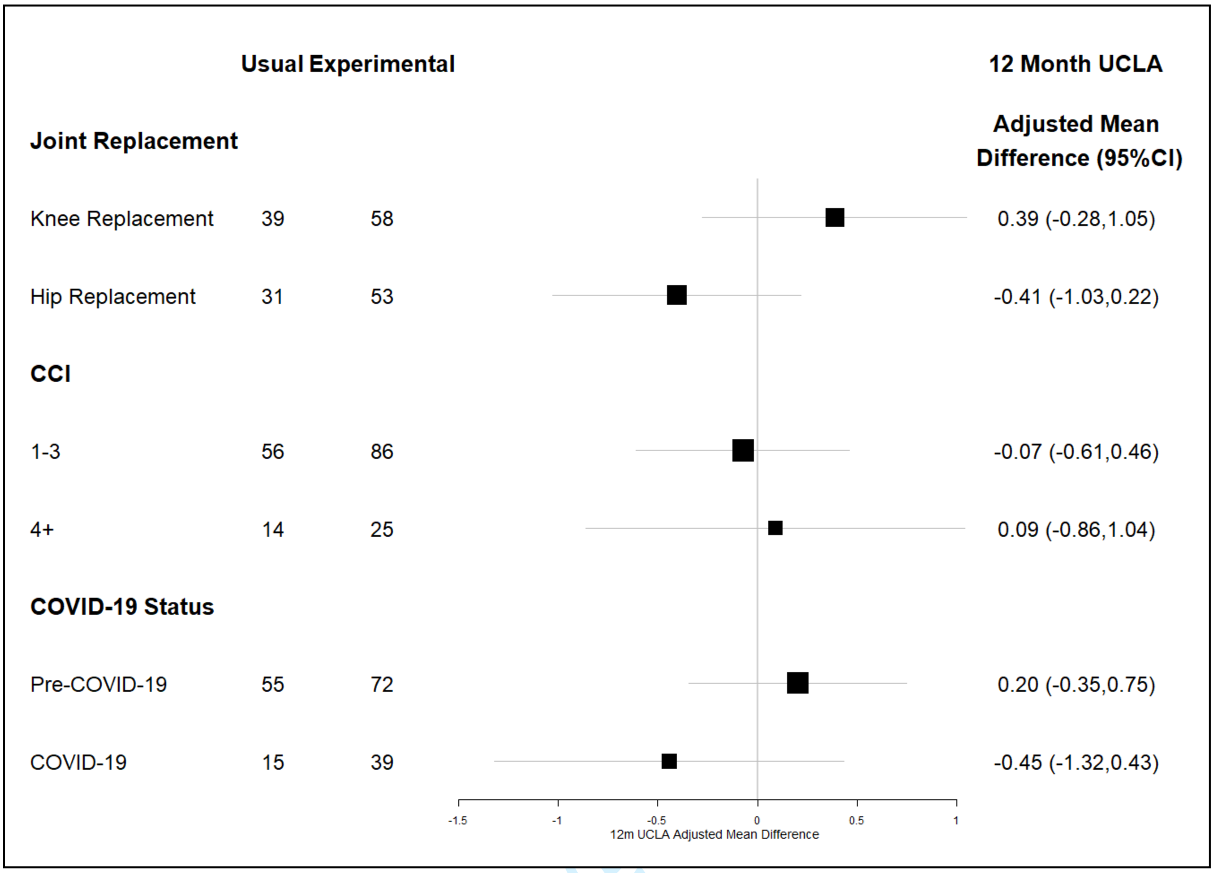

CCI – Charlson Comorbidity Index; CI – Confidence Intervals; UCLA – University for Los Angeles Activity Score

For peer review only

CONSERVE-CONSORT Checklists

CONSERVE-CONSORT Extension: 22Jan2022 (PEP-TALK Final Report)			
**Item**	**Item Title**	**Description**	**Page No.**
I.	Extenuating Circumstances	Describe the circumstances and how they constitute extenuating circumstances.	Methods, Randomisation and masking Para 1; Statistical Methods, Para 3; Results, Recruitment and participant flow, Para 2; Supplementary File 2
II.	Important Modifications	a. Describe how the modifications are important modifications.	Methods, Randomisation and masking Para 1; Statistical Methods, Para 3;
		b. Describe the impacts and mitigating strategies, including their rationale and implications for the trial.	Methods, Randomisation and masking Para 1; Statistical Methods, Para 3;
		c. Provide a modification timeline.	Results, Recruitment and participant flow, Para 2

PEP-TALK_FinalReport_ConserveConsortChecklist_V0.3_04Jan2022

For peer review only - http://bmjopen.bmj.com/site/about/guidelines.xhtml

III.	Responsible Parties	State who planned, reviewed and approved the modifications.			Methods, Randomisation and masking, Para 1
IV.	Interim data	If modifications were informed by trial data, describe how the interim data were used, including whether they were examined by study group, and whether the individuals reviewing the data were blinded to the treatment allocation.			No interim analysis performed.
**CONSORT Number and Item**		For each row, if important modifications occurred check "direct impact" and/or "mitigating strategy" and describe the changes in the trial manuscript or supplement. Check "no change" for items that are unaffected in the extenuating circumstance.			**Page No.**
		**No Change**	**Impact***	**Mitigating Strategy****	
1	Title and abstract		X	X	2
2	Introduction	X			4-5
3	Methods: Trial Design	X			5
4	Methods: Participants	X			5
5	Methods: Interventions	X			5-6
6	Methods: Outcomes	X			6
7	Methods: Sample Size	X			7
8-10	Methods: Randomisation	X			7
11	Methods: Blinding	X			7
12	Methods: Statistical methods		X		7-8
13	Results: Participant flow		X	X	8-9
14	Results: Recruitment		X	X	8-9

For peer review only

For peer review only - http://bmjopen.bmj.com/site/about/guidelines.xhtml

15	Results: Baseline data	X			9, Table 1
16	Results: Numbers analysed	X			9, Table 2
	Results: Outcomes and estimation		X	X	9-10 Tables 3, 4, 5, 6<br>Figures 3,4,5
18	Results: Ancillary analyses		X	X	10-11, Sup File 2
19	Results: Harms	X			10
20	Discussion: Limitations		X		11-13
21	Discussion: Generalisability		X		11-13
	Other information: Registration	X			2
24	Other information: Protocol	X			Published, ref pg 5
25	Other information: Funding	X			13

*Aspects of the trial that are directly affected or changed by the extenuating circumstance and are not under the control of investigators, sponsor or funder.
**Aspects of the trial that are modified by the study investigators, sponsor or funder to respond to the extenuating circumstance or manage the direct impacts on the trial.

For peer review only - http://bmjopen.bmj.com/site/about/guidelines.xhtml
